# International Society of Paediatric Surgical Oncology (IPSO) Minimally Invasive Surgery (MIS) Guidelines

**DOI:** 10.3332/ecancer.2026.2076

**Published:** 2026-02-18

**Authors:** Israel Fernandez-Pineda, Simone de Campos Vieira Abib, Tristan Boam, Diego Aspiazu Salinas, Samer Michael, Justin Ted Gerstle, Steven Warmann, Jorg Fuchs, Alyssa Stetson, Gloria Gonzalez, Greg Tiao, Timothy B. Lautz, Rodrigo Chaves Ribeiro, Roshni Dasgupta, Jaime Shalkow-Klincovstein, Cristian Puerta, Andrew M. Davidoff, Marianna Cornet, Julien Grosman, Aurore Pire, Sabine Sarnacki, Thomas Blanc, Abdelhafeez H Abdelhafeez

**Affiliations:** 1 Department of Paediatric Surgery, Children’s Health Ireland at Crumlin, Dublin D12 N512, Ireland; 2 Pediatric Oncology Institute, GRAACC, Federal University of São Paulo, São Paulo, SP 04021-001, Brazil; 3 Department of Paediatric Surgery, Clínica San Felipe, Clinica Angloamericana, Clinica Internacional, Lima 15073, Perú; 4 Department of Paediatric Surgery, Hadassah University Medical Center, Kiryat Hadassah, Jerusalem 91120, Israel; 5 Department of Surgery, Memorial Sloan Kettering Cancer Center, 1275 York Avenue, New York, NY 10065, USA; 6 Department of Pediatric Surgery and Pediatric Urology, Charité University Hospital Berlin, Augustenburger Platz 1, 13353 Berlin, Germany; 7 Department of Pediatric Surgery and Pediatric Urology, University of Tuebingen, Hoppe-Seyler-Str. 3, Tübingen 72076, Germany; 8 Division of Paediatric General and Thoracic Surgery, Cincinnati Children Hospital Medical Center, University of Cincinnati, Cincinnati OH 45229, USA; 9 Department of Paediatric General and Thoracic Surgery, Cleveland Clinic Children's Hospital, Cleveland OH 44106, USA; 10 Division of Paediatric Surgery, Ann & Robert H Lurie Children’s Hospital of Chicago, Chicago, IL 60611, USA; 11 Department of Paediatric Surgery. Children’s Cancer Hospital, Barretos Cancer Hospital, Barretos 14784058, Brazil; 12 ABC Cancer Center, Sur 136 No 116, Mexico City 01120, Mexico; 13 Division of Cardiothoracic Surgery, University of California, San Diego, 9300 Campus Point Drive, La Jolla, CA 92093, USA; 14 Department of Surgery, St Jude Research Hospital 262 Danny Thomas Place. MS133, Memphis, TN 38105, USA; 15 Department of pediatric surgery, urology and transplantation, Necker Enfants-Malades Hospital, AP-HP, University Paris Cité, Paris, 75015, France; 16 Division of Pediatric Surgery, Department of Surgery, Golisano Children's Hospital, University of Rochester Medical Center, Rochester, NY 14627, USA

**Keywords:** paediatric surgical oncology, minimally invasive surgery, children

## Abstract

Paediatric oncology surgeons play a crucial role in diagnosing, staging, and treating malignant solid tumors. In recent years, many solid tumour protocols have advocated for a more tailored surgical approach to both the primary tumour site and metastatic disease. The integration of Minimally Invasive Surgery (MIS) into paediatric oncology practice has gained popularity over the past few decades. While the benefits of MIS are well established in non-oncologic surgery, its role in paediatric solid tumours is still evolving and, in many cases, lacks high-quality evidence. These IPSO-MIS guidelines guidelines aim to provide practical surgical recommendations for diverse clinical scenarios, addressing the needs of both High-Income Countries (HICs) and Low- and Middle-Income Countries (LMICs). The contributing authors represent both settings, ensuring a comprehensive and inclusive perspective. We hope that these guidelines will contribute to improving outcomes for children with cancer worldwide.

Israel Fernandez-Pineda, IPSO Education Committee Chair

Abdelhafeez H Abdelhafeez, IPSO Education Committee Member

## Disclaimer

The document, IPSO-MIS Guidelines, and the information it contains are for authorised use by surgeons. IPSO cannot accept any liability and responsibility for any claims, loss or damage arising from the use of this document and its contents.

## Contents

### IPSO-MIS GUIDELINES

### Abstract

#### Disclaimer

### Contributing Authors

1. General principles of MIS in PSO
2. The role of MIS in Paediatric Cancer Diagnosis and Staging
3. Role of MIS in neurogenic tumors
4. MIS in Paediatric Nephroblastoma
5. The Role of MIS in the Management of Paediatric Liver Tumors
6. Role of MIS in Paediatric Pulmonary Metastatic Disease
7. Ovarian Neoplasms-The Role of MIS
8. The Role of MIS in Paediatric Mediastinal Masses and Thoracic Tumours
9. Role of MIS in Fertility Preservation
10. New technologies in MIS in Childhood Cancer Surgery

### General Principles of MIS in PSO 1

Introduction 1

Indications 2

Contraindications 3

Surgical Approach 4

Conclusions 4

### The Role of MIS in Paediatric Cancer Diagnosis and Staging 6

Introduction 6

MIS Indication for Diagnosis and Staging 7

Summary of MIS Indications 8

Summary of MIS Contraindications 8

Surgical Approach 8

Conclusions 10

### Role of MIS in Neurogenic Tumors 12

Introduction 12

Advantages and Disadvantages of MIS 13

Feasibility, safety and outcomes 13

Indications and contraindications 14

Robotic-assisted MIS 15

Surgical Approach 17

Conclusions 20

### MIS in Paediatric Nephroblastoma 23

Introduction 23

Indications-contraindications 24

Surgical Approach 24

### The Role of MIS in the Management of Paediatric Liver Tumors 27

Introduction 27

Minimally Invasive Procedures for Liver Biopsy 27

MIS for Liver Resection 28

MIS for Radiofrequency Ablation 32

Conclusions 34

### Role of MIS in Paediatric Pulmonary Metastatic Disease 38

Introduction 38

MIS Indications 39

MIS Contraindications 39

Surgical Approach 40

Conclusions 41

### Ovarian Neoplasms-The Role of MIS 43

Introduction 43

Indications for MIS 44

MIS Contraindications 45

Surgical Approach 45

Tips and Pitfalls 46

Conclusions 46

### The Role of MIS in Paediatric Mediastinal Masses and Thoracic Tumours 50

Introduction 50

MIS Indications in Paediatric Solid Tumours 51

Feasibility and Outcomes of MIS in Mediastinal Tumours in Children 51

Feasibility and Outcomes of MIS in Paediatric Patients with Lung Metastases 52

Open versus thoracoscopic approach for pulmonary lesions in Osteosarcoma 53

MIS Contraindications 55

Surgical Approach 55

Outcomes in LMICs 59

MIS in Global Paediatric Surgical Oncology 59

Conclusions 59

### Role of MIS in Fertility Preservation 62

Introduction 62

MIS Indications 64

MIS Contraindications 65

Surgical Approach 66

Ovarian Transposition 67

Ovarian Harvesting for Cryopreservation 67

Conclusions 68

### New Technologies in MIS in Childhood Cancer Surgery 71

Introduction 71

Robotic-Assisted Surgery 72

Discussion 74

Conclusions 76

## Contributing authors

## 1. General principles of MIS in PSO

1. Israel Fernandez-Pineda

Department of Paediatric Surgery, Children’s Health Ireland at Crumlin, Dublin D12 N512, Ireland

2. Simone de Campos Vieira Abib

Pediatric Oncology Institute, GRAACC, Federal University of São Paulo, São Paulo, SP 04021-001, Brazil

## 2. The role of MIS in Paediatric Cancer Diagnosis and Staging

1. Tristan Boam

Department of Paediatric Surgery, Children’s Health Ireland at Crumlin, Dublin D12 N512, Ireland

2. Diego Aspiazu Salinas

Department of Paediatric Surgery, Clínica San Felipe, Clinica Angloamericana, Clinica Internacional, Lima 15073, Perú

## 3. Role of MIS in Neurogenic Tumours

1. Samer Michael

Department of Paediatric Surgery, Hadassah University Medical Center, Kiryat Hadassah, Jerusalem 91120, Israel

2. Justin Ted Gerstle

Department of Surgery, Memorial Sloan Kettering Cancer Center, 1275 York Avenue, New York, NY 10065, USA

## 4. MIS in Paediatric Nephroblastoma

1. Steven Warmann

Department of Pediatric Surgery and Pediatric Urology, Charité University Hospital Berlin, 13353 Berlin, Germany

2. Jorg Fuchs

Department of Pediatric Surgery and Pediatric Urology, University of Tuebingen, Tübingen 72076, Germany

## 5. The Role of MIS in the Management of Paediatric Liver Tumours

1. Alyssa Stetson

Division of Paediatric General and Thoracic Surgery, Cincinnati Children Hospital Medical Center, Cincinnati OH 45229, USA

2. Gloria Gonzalez

Department of Paediatric General and Thoracic Surgery, Cleveland Clinic Children's Hospital, Cleveland OH 44106, USA

3. Greg Tiao

Division of Paediatric General and Thoracic Surgery, Cincinnati Children Hospital Medical Center, Cincinnati OH 45229, USA

## 6. Role of MIS in Paediatric Pulmonary Disease

1. Timothy B. Lautz

Division of Paediatric Surgery, Ann & Robert H Lurie Children’s Hospital of Chicago, Chicago, IL 60611, USA

2. Rodrigo Chaves Ribeiro

Department of Paediatric Surgery. Children’s Cancer Hospital, Barretos Cancer Hospital, Barretos 14784058, Brazil

## 7. Ovarian Neoplasms-The Role of MIS

1. Alyssa Stetson

Division of Paediatric General and Thoracic Surgery, Cincinnati Children Hospital Medical Center, Cincinnati OH 45229, USA

2. Roshni Dasgupta

Division of Paediatric General and Thoracic Surgery, Cincinnati Children Hospital Medical Center, Cincinnati OH 45229, USA

## 8. The Role of MIS in Paediatric Mediastinal Masses and Thoracic Tumours

1. Jaime Shalkow-Klincovstein

ABC Cancer Center, Sur 136 No 116, Mexico City 01120, Mexico

2. Cristian Puerta

Division of Cardiothoracic Surgery, University of California, San Diego, 9300 Campus Point Drive, La Jolla, CA 92093, USA

3. Andrew M. Davidoff

Department of Surgery, St Jude Research Hospital 262 Danny Thomas Place. MS133, Memphis, TN 38105, USA

## 9. Role of MIS in Fertility Preservation

1. Marianna Cornet

Department of pediatric surgery, urology and transplantation, Necker Enfants-Malades Hospital, AP-HP, Paris, 75015, France

2. Julien Grosman

Department of pediatric surgery, urology and transplantation, Necker Enfants-Malades Hospital, AP-HP, Paris, 75015, France

3. Aurore Pire

Department of pediatric surgery, urology and transplantation, Necker Enfants-Malades Hospital, AP-HP, Paris, 75015, France

4. Sabine Sarnacki

Department of pediatric surgery, urology and transplantation, Necker Enfants-Malades Hospital, AP-HP, Paris, 75015, France

## 10. New Technologies in MIS in Childhood Cancer Surgery

1. Thomas Blanc

Department of pediatric surgery, urology and transplantation, Necker Enfants-Malades Hospital, AP-HP, Paris, 75015, France

2. Abdelhafeez H. Abdelhafeez

Division of Pediatric Surgery, Department of Surgery, Golisano Children's Hospital, Rochester, NY 14627, USA

## Figures and Tables

**Table 1.** MIS indications for PSO according to anatomic location.

Laparoscopic	Neurogenic tumour (mainly adrenalectomy and resection of tumours without IDRFs). Avoid for ACC.
	Wilms tumour after neoadjuvant chemotherapy (SIOP protocol_certain cases)
	Liver tumour (diagnostic or therapeutic)
	Splenectomy for splenic malignant infiltrate and splenomegaly
	Oophoropexy prior to pelvic irradiation
	Pelvic mass
	Pancreatic tumour
Thoracoscopic	Lung nodule
	Neurogenic tumour without IDRFs
	Mediastinal mass (diagnostic or therapeutic)

ACC: adrenocortical carcinoma; IDRFs: image-defined risk factors; SIOP: International Society of Paediatric Oncology
