# Supplementary material for: International Society of Paediatric Surgical Oncology (IPSO) Minimally Invasive Surgery (MIS) Guidelines
Source: Ecancermedicalscience. 2026 Feb 18;20:2076. doi: 10.3332/ecancer.2026.2076 (PMC13006547; doi:10.3332/ecancer.2026.2076)
Supplement: Related texts

# Other: General principles of minimally invasive surgery in paediatric surgical oncology

**DOI:** 10.3332/ecancer.2023

**Published:** 2025-10-23

**Authors:** Israel Fernandez-Pineda, Simone Abib

**Affiliations:** 1 Department of Paediatric Surgery, Children's Health Ireland at Our Lady's Children's Hospital, Dublin D12 N512, Ireland; 2 Department of Paediatric Surgery, Paediatric Oncology Institute – GRAACC, Federal University of São Paulo, São Paulo 04039-001, Brazil

**Keywords:** paediatric surgical oncology, minimally invasive surgery

## Abstract

Minimally invasive surgery (MIS) has become increasingly integrated into Paediatric Surgical Oncology (PSO), offering benefits such as faster recovery, reduced postoperative pain, earlier resumption of adjuvant therapy, lower blood loss and improved cosmetic outcomes. Despite these advantages, the safe application of MIS in oncology requires strict adherence to oncological principles to avoid complications such as tumour spillage, incomplete resections and staging errors, which may compromise survival outcomes. This article reviews the general principles, indications and contraindications for MIS in paediatric oncology, highlighting tumour- and histology-specific considerations. Commonly accepted MIS applications include selected cases of neuroblastoma, Wilms tumour following neoadjuvant therapy under SIOP protocols, thoracoscopic lung metastasectomy and resection of certain mediastinal, hepatic and adnexal masses. Contraindications include large or fragile tumours, high-risk neuroblastomas with vascular encasement and situations where surgeon experience or resources are insufficient. Technical aspects, patient selection and multidisciplinary coordination are emphasised as key to ensuring safety and efficacy. Establishing MIS guidelines in PSO may aid surgeons in decision-making and promote consistent standards of care.

## Introduction

Minimally invasive surgery (MIS) has evolved over the last decades, resulting in the preferred approach for certain conditions in children such as appendicitis, cholecystitis, pyloric stenosis, splenectomy and so on. The relatively low volume of Paediatric Surgical Oncology (PSO) cases encountered by general paediatric surgeons can negatively impact their decisions or confidence to use an MIS approach. MIS approach should be considered when the surgeon has proficiency in both open oncological procedures and MIS techniques; otherwise, important oncological principles violations may occur and impact the chances of cure and survival of children with cancer.

Historically, cancer surgery has been associated with big incisions and aggressive resections, which is still nowadays of great value for certain histologies, including high-risk neuroblastomas, sarcomas, locally advanced Wilms tumours, pulmonary metastatic disease from osteosarcoma and so on.

MIS in paediatric oncology remains an evolving field that has grown over the past 40 years. For MIS to gain wider acceptance, it must be able to at least replicate or exceed outcomes that are achieved with the current standard of care [1, 2]. With the advancement of MIS, paediatric surgical oncologists have turned their efforts to utilise these techniques for the benefit of children with cancer. Advantages of MIS for the paediatric cancer patients include early postoperative recovery, decreased postoperative pain, early re-initiation of adjuvant therapy (chemotherapy, radiation therapy, immunotherapy and stem cell transplantation), decreased intraoperative blood loss and improved cosmetic result [3]. From a technical standpoint, MIS provides magnification of the local anatomy, which refines the assessment of the limits of resection and a better visualisation for certain anatomic locations such as thoracic outlet, mediastinum and pelvis. Limitations of MIS in PSO include loss of tactile feedback, risk of vascular injury, limited working space when resecting large tumours, risk of tumour spillage and challenges related to removal of the resected specimen.

As a rule, if a MIS procedure is performed in a safe manner and following the same oncological principles used in open surgery, this should be promoted [4]. The reality is that MIS is associated with longer learning curves, which exposes patients to long procedures and is occasionally performed by paediatric surgeons who are not familiarised with the surgical guidelines of the cancer protocols. This may result in incomplete surgical resections, failures in local staging, tumour spillage and eventually local and systemic recurrences that may impact the long-term survival rates. These MIS guidelines may represent a helpful tool to clearly establish the indications and contraindications of a less invasive surgical approach for paediatric cancer patients. The guidelines also provide practical surgical tips to avoid complications and guide the surgeon through the procedure.

## Indications

The prognostic significance of radical resection and inherent technical complexity to achieve complete resection are both dependent on tumour and histology. There are certain MIS procedures in PSO that have gained popularity over the last years including low and intermediate risk neuroblastoma depending on the presence and nature of image-defined risk factors, radical nephrectomy for some renal tumours after neoadjuvant chemotherapy in the International Society of Paediatric Oncology (SIOP) protocol, thoracoscopic lung metastasectomy, biopsy and/or resection of mediastinal masses, liver masses, certain pancreatic tumours with a favourable anatomic location and resection of adnexal masses (Table 1) [5–8].

Thoracoscopic resection of lung nodules or mediastinal masses for both diagnostic and therapeutic purposes represents a good approach to avoid a thoracotomy [9]. The use of thoracoscopy is influenced by the tumour type. Patients with non-chemosensitive tumours such as osteosarcoma and nonrhabdomyosarcoma soft tissue sarcomas may benefit from aggressive attempts to remove even a single nodule and thoracotomy is usually recommended. Nevertheless, patients with oligometastatic pulmonary metastatic may be good candidates for the MIS approach.

Patients with lung metastatic disease in the context of chemosensitive tumours such as Wilms tumour, hepatoblastoma, germ cell tumours and rhabdomyosarcoma may benefit from thoracoscopic resections.

Localisation of lung lesions is usually required for subpleural nodules that are not visible on the lung surface. Although percutaneous biopsies may be performed with the assistance of image-guided technology, there remain situations in which a surgical biopsy is required either via thoracoscopic or open surgical technique. A limited thoracotomy incision or potentially a traditional posterolateral thoracotomy, which requires a large incision, rib retraction and possible division of the latissimus dorsi may be required to obtain adequate specimens. This morbid incision has led to long-term complications in children such as shoulder elevation, winged scapula, chest wall asymmetry and scoliosis; therefore, consideration for a thoracoscopic approach is certainly deserving in this special population [10, 11]. Thoracoscopic resection of neuroblastoma has been associated with a decreased hospital stay, less intraoperative blood loss and less requirements for chest drains.

A localised <5 cm adrenal mass is usually a good case for MIS, but if a suspicion for adrenocortical carcinoma (ACC) is raised, MIS should be discouraged. ACC is a poor chemo/radiation sensitive tumour; therefore, complete resection with negative margins is critical for long-term survival. ACC is generally a fragile tumour and at significantly high risk of rupture, which negatively affects survival and so an open approach is recommended to minimise this risk [12, 13].

Surgical management of Wilms tumour in North America is based on the Children’s Oncology Group (COG) guidelines and upfront resection is recommended. These tumours are generally large, fragile and at risk of rupture; therefore, MIS does not play a role in COG guidelines. On the other hand, the SIOP protocol recommends neoadjuvant chemotherapy for suspected Wilms tumours and MIS tumour nephrectomy may be feasible after chemotherapy-induced shrinkage. SIOP 2001 trial outcomes for patients with unilateral Wilms tumour who underwent MIS resection were comparable to open surgery; however, lymph node sampling was deficient in this study, which may impact the recurrence rate. Adequate lymph node sampling is mandatory for adequate local staging [7]. Nephron-sparing surgery (NSS) is frequently required in bilateral Wilms tumours. Laparoscopic NSS for malignant renal tumours remains controversial. There are potential challenges such as obtaining a negative resection margin, avoidance of tumour spillage and potentially increased chance of recurrence. It is technically very demanding and it should be performed by a very experienced team.

MIS for adnexal masses has an important diagnostic and staging role in malignant ovarian tumours; however, the risk of spillage limits the appropriateness of laparoscopic resection. For benign ovarian germ cell tumours such as teratoma, ovarian sparing surgery is recommended, but MIS tools are less able to delineate the interface between tumour and normal ovarian tissue; therefore, an open approach should be favoured over MIS if lack of experience [14].

## Contraindications

Large masses can potentially impede safe accessibility and specimen delivery, and contribute to the potential risk of intra-operative tumour spillage; therefore, tumour size should be considered when selecting the optimal surgical approach.

For certain thoracoscopic procedures, single lung ventilation is critical to obtain lung collapse and this is sometimes difficult to achieve in small patients. This can hamper visualisation of pulmonary nodules, decrease working space in the thorax and increase risk of lung injury. Therefore, patient selection plays a very important role in the success of the MIS procedure. Deep and small pulmonary nodules not amenable to localisation are difficult to visualise and since the tactile ability is lost in thoracoscopy, these pulmonary lesions may be missed and an open approach may be preferred. Osteosarcoma patients with multiple serial pulmonary metastasectomies are expected to have firm lung adhesions to the chest wall, which may limit the ability for a thoracoscopic approach.

High-risk neuroblastoma usually presents with midline involvement and encasement of major vascular structures, including aorta, cava, celiac axis, superior mesenteric artery and renal vessels. The extent of resection may affect the outcome of high-risk neuroblastoma patients; therefore, an open approach is generally recommended.

Other contraindications to perform MIS in paediatric oncology include inadequate equipment, insufficient training, lack of experience and patient-related factors such as tumour size, abnormalities in cardiac output, patient instability and coagulopathy [15].

## Surgical approach

Recent publications have shown that low complication and conversion rates of MIS tumour resection may be achieved with careful patient selection [16]. Other factors influencing the outcomes include the surgeon’s experience, patient size, location and proximity to vital structures. All these factors impact the final decision of whether or not to pursue this minimally invasive approach. Close communication with anaesthesia team is critical to maximise the safety of the procedure. MIS requires the ability to create enough working space to safely visualise and perform the operative procedure, which is obtained by means of carbon dioxide insufflation.

This may lead to difficulty with anesthesia in infants and young children. Other factors influencing the anesthetic procedure include single lung ventilation, hypothermia and the effect of lateral decubitus positioning for thoracoscopic procedures.

Technical factors influencing the success of MIS in PSO include right trocar placement, camera with zoom magnification, meticulous haemostasis, avoidance of heat dispersion, use of Endo-catch bags for tumour extraction and enlargement of trocar site.

## Conclusion

MIS in PSO is considered a safe diagnostic and therapeutic modality. Careful patient selection and correct surgical indications are critical to ensure oncologic principles are not violated and to minimise the potential for complications. It is important for the paediatric surgeon, as a member of the multidisciplinary team involved in the care of children with cancer, to understand the indications and contraindications of MIS in the treatment of paediatric solid tumours. We are optimistic that the creation of these guidelines for the use of MIS in PSO will help paediatric surgeons in the decision-making process for the best possible surgical approach.

## Conflicts of interest

The authors have no conflicts of interest to declare.

## Funding

This research received no specific grant from any funding agency in the public, commercial or not-for-profit sectors.

## References

[1] Spurbeck WW, Davidoff AM, Lobe TE (2004). Minimally invasive surgery in pediatric cancer patients. Ann Surg Oncol.

[2] Abdelhafeez A, Ortega-Laureano L, Murphy AJ (2019). Minimally invasive surgery in pediatric surgical oncology: practice evolution at a contemporary single-center institution and a guideline proposal for a randomized controlled study. J Laparoendosc Adv Surg Tech A.

[3] Iwanaka T, Arai M, Kawashima H (2004). Endosurgical procedures for pediatric solid tumors. Pediatr Surg Int.

[4] de Lijster MS, Bergevoet RM, Dalen EC (2012). Minimally invasive surgery versus open surgery for the treatment of solid abdominal and thoracic neoplasms in children. Cochrane Database Syst Rev.

[5] Malek MM, Mollen KP, Kane TD (2010). Thoracic neuroblastoma: a retrospective review of our institutional experience with comparison of the thoracoscopic and open approaches to resection. J Pediatr Surg.

[6] Leclair MD, de Lagausie P, Becmeur F Laparoscopic resection of abdominal neuroblastoma. Ann Surg Oncol.

[7] Warmann SW, Godzinski J, Tinteren H (2014). Surgical panel of the SIOP renal tumor strategy group. Minimally invasive nephrectomy for Wilms tumors in children - data from SIOP 2001. J Pediatr Surg.

[8] Rescorla FJ (2012). Pediatric germ cell tumors. Semin Pediatr Surg.

[9] Castagnetti M, Delarue A, Gentet JC (2004). Optimizing the surgical management of lung nodules in children with osteosarcoma: thoracoscopy for biopsies, thoracotomy for resections. Surg Endosc.

[10] Daw NC, Chou AJ, Jaffe N (2015). Recurrent osteosarcoma with a single pulmonary metastasis: a multi-institutional review. Br J Cancer.

[11] Fernandez-Pineda I, Daw NC, McCarville B (2012). Patients with osteosarcoma with a single pulmonary nodule on computed tomography: a single-institution experience. J Pediatr Surg.

[12] Berruti A, Baudin E, Gelderblom H (2012). Adrenal cancer: ESMO clinical practice guidelines for diagnosis, treatment and follow-up. Ann Oncol.

[13] Abib SCV, Weldon CB (2021). Management of adrenal tumors in pediatric patients. Surg Oncol Clin N Am.

[14] Mayer JP, Bettolli M, Kolberg-Schwerdt A (2009). Laparoscopic approach to ovarian mass in children and adolescents: already a standard in therapy. J Laparoendosc Adv Surg Tech A.

[15] Irtan S, Brisse HJ, Minard-Colin V (2015). Minimally invasive surgery of neuroblastic tumors in children: indications depend on anatomical location and image-defined risk factors. Pediatr Blood Cancer.

[16] Acker SN, Bruny JL, Garrington TP (2015). Minimally invasive surgical techniques are safe in the diagnosis and treatment of pediatric malignancies. Surg Endosc.

# Other: The role of minimally invasive surgery for diagnosis and staging in paediatric surgical oncology

**DOI:** 10.3332/ecancer.2025.2032

**Published:** 2025-10-23

**Authors:** Tristan Boam, Diego Aspiazu Salinas

**Affiliations:** 1 Department of Paediatric Surgery, Children’s Health Ireland at Crumlin Hospital, Dublin, D12 N512 Ireland; 2 Department of Paediatric Surgery, Clínica San Felipe, Clinica Angloamericana, Clinica Internacional, Lima 15073, Perú

**Keywords:** minimally invasive surgery, paediatric oncology, diagnosis, staging, biopsy, indocyanine green

## Abstract

Minimally invasive surgery (MIS) has become increasingly important in paediatric surgical oncology for the diagnosis and staging of solid tumours, due to its advantages in reducing morbidity, pain and hospitalisation times compared to traditional open surgery. While ultrasound-guided core needle biopsy (USCNB) typically remains the primary method for tissue sampling, MIS becomes essential in cases where USCNB is impractical or ineffective, such as with inaccessible tumour locations or where detailed staging information is required. Recent studies highlight the effectiveness of MIS in obtaining high-quality biopsy samples in neuroblastoma, thoracic tumours, hepatoblastoma and rhabdomyosarcoma, frequently outperforming open surgical methods regarding sample adequacy and complication rates. Video-assisted thoracoscopic surgery has demonstrated particular efficacy with minimal complications across various thoracic malignancies. Additionally, laparoscopic and robotic approaches for retroperitoneal lymph node dissection in rhabdomyosarcoma have proven beneficial by significantly reducing postoperative complications and hospital stays compared to open methods. Innovative adjunct technologies such as indocyanine green (ICG) fluorescence imaging have further advanced MIS by providing superior visualisation of tumour margins, metastases and lymphatic structures, enhancing the precision and safety of procedures. Overall, the integration of MIS techniques, supported by advanced imaging methods like ICG, represents a significant advancement in paediatric oncology, offering reliable diagnostic and staging options with reduced patient morbidity. These approaches provide critical clinical advantages, positioning MIS as an essential component of contemporary paediatric surgical oncology practice.

## Introduction

Minimally invasive surgery (MIS) is increasingly integrated into paediatric surgical oncology, providing significant benefits such as reduced pain, shorter hospital stays and quicker recovery compared to traditional open surgery. Ultrasound-guided core needle biopsy (USCNB) is often the procedure of choice for tissue diagnosis; however, MIS is an important option if the tumour is not accessible with this technique. This guideline reviews the role of MIS specifically for biopsy and staging, addressing its utility in various paediatric solid tumours.

## MIS indications for diagnosis and staging

### Neuroblastoma

A European retrospective study compared four biopsy methods for neuroblastoma: USCNB (most frequently used, 37.5%), laparoscopic-assisted core needle biopsy (39.6%), open incisional biopsy (12.5%) and minimally invasive thoracoscopic/laparoscopic incisional biopsy (10.4%). Perioperative complications were rare overall, with one major complication (duodenal perforation) reported in the open biopsy group and minor complications conservatively managed in the USCNB and laparoscopic-assisted core needle biopsy. MIS techniques demonstrated effectiveness in providing adequate tissue for histopathological and molecular analyses, achieving a 100% success rate in all modalities. In contrast, open incisional biopsies had the highest inadequacy rate at 16.7%, followed by USCNB at 11%, although these differences were not statistically significant [1].

Another case report highlighted a potential role for MIS staging, describing the use of robotic-assisted lymph-node dissection for an L1 neuroblastoma after adrenalectomy [2].

### Thoracic tumours

In a single institution series, video-assisted thoracoscopic surgery (VATS) demonstrated high effectiveness and low morbidity in obtaining biopsies for various thoracic tumours, including neuroblastoma, ganglioneuroma, germ cell tumours, lymphoma and thymic tumours. There were low complication (7%) and conversion rates (2.3%), supporting its feasibility for biopsy [3].

### Wilms tumour

A Children’s Cancer and Leukaemia Group and the International Society of Paediatric Oncology (SIOP) report on Wilms tumour biopsy experience from the SIOP 2001 trial showed that the majority of biopsies are performed by USCNB, with a 6.5% rate of non-diagnostic sampling. No biopsies were performed by MIS; 10 were open incisional biopsies. In the majority of cases, biopsy for Wilms tumour is generally unnecessary and is typically reserved for cases with imaging features and clinical factors that raise suspicion of other malignancies such as clear cell sarcoma, rhabdoid tumour or renal cell carcinoma [4]. USCNB is preferred in these scenarios, as the tumours are often readily accessible and it is likely to be associated with less risk of spillage, upstaging and local recurrence compared to open and MIS; notably, the UKW3 trial did not associate USCNB with increased local recurrence and its use should not mandate upstaging of the disease [5].

### Hepatoblastoma

MIS may have a potential role for staging in hepatoblastoma, in scenarios where cross-sectional imaging is equivocal or unavailable; laparoscopy may have utility in confirming involved liver segments and extra-hepatic metastases.

A multicentre retrospective study examining biopsy methods for hepatoblastoma concluded that USCNB was associated with significantly reduced bleeding complications compared to MIS or open methods, supporting USCNB as the preferred initial diagnostic modality [6].

### Rhabdomyosarcoma

In a small case series of laparoscopic retroperitoneal lymph node dissection (RPLND) for paratesticular rhabdomyosarcoma, the authors found, while technically challenging, the technique is associated with reduced morbidity compared to open approaches [7].

A further comparative retrospective study demonstrated single-site retroperitoneoscopic RPLND as equally effective with significantly reduced postoperative analgesia hospital stay compared to traditional laparoscopic techniques [8].

Robotic-assisted RPLND was evaluated in an adolescent series, demonstrating comparable lymph node yield and minimal complications [9].

### Lung metastases

In a 2023 report, VATS showed efficacy for identification and excisional biopsy of pulmonary metastases, especially when combined with pre-operative computed tomography-guided coil localisation. This combination significantly improved surgical accuracy, ensuring complete resection with maximum sparing of normal lung tissue [10]. Another study indicated that VATS pulmonary metastasectomy had comparable outcomes to open thoracotomy in cases of oligometastatic osteosarcoma [11].

## Summary of MIS indications

- For tumour biopsy where USCNB is not suitable or unavailable.
- Lymph node sampling and dissection.
- VATS excision biopsy of lung nodules/metastases.
- MIS staging if cross-sectional imaging is equivocal or unavailable.

## MIS contraindications for diagnosis and staging

Contraindications to MIS are generally few, especially when it is used primarily for diagnostic and staging purposes. However, the role of MIS for biopsy does have important considerations for a number of scenarios in paediatric surgical oncology. Biopsies should only be taken when necessary, if the diagnosis cannot be reached by clinical or non-invasive means. When they are necessary, biopsies should be performed by the least invasive route. With modern imaging techniques and high-resolution ultrasound, USCNB is often the procedure of choice. If USCNB is unavailable, for example, in resource-poor environments and developing nations, MIS is likely the next-best option for staging, diagnosis and biopsy, as it makes use of transferable skills and widely available equipment.

MIS should also be avoided in cases where port access is made unsafe by factors such as dense abdominal adhesions, distended bowel loops or in patients where pneumoperitoneum or pneumothorax induction could lead to respiratory compromise.

## Summary of MIS contraindications

- If an alternative to biopsy is available.
- If USCNB is available.
- In patients with significant cardiac/respiratory compromise.
- In patients where port access is unsafe, e.g., dense adhesions.

## Surgical approach

### Patient position

Patient positioning depends on tumour location; abdominal tumours usually require a supine or slightly lateral position, while VATS procedures use lateral decubitus or modified prone positions.

### Trocar sites

Trocar placement also depends on the tumour location. Optimal placement for biopsies should focus on minimising the risk of tumour spillage and safe extraction of the tumour sample.

### Surgical technique, tips and pitfalls

For MIS biopsy, several key techniques are necessary to ensure sample quality and procedural safety. Cold endoscopic scissors are recommended to prevent thermal damage to biopsy samples that can occur with diathermy use. After sampling, placing the biopsy specimen into an endoscopic retrieval bag reduces the risk of spillage and tumour seeding.

Once the sample is taken, diathermy should remain readily available to manage potential bleeding. Additional haemostatic adjuncts such as Tisseal™ (fibrin sealant) and TachoSil™ (fibrin-coated collagen patch) are beneficial for achieving rapid and effective haemostasis.

In certain scenarios, a laparoscopic-assisted core needle biopsy may be utilised; MIS is beneficial for moving obstacles to biopsy (e.g., overlying bowel loops) and the needle can be guided under direct vision.

During VATS lung/pleural biopsy procedures, energy devices such as LigaSure™ facilitate efficient vessel sealing and tissue dissection. Additionally, the authors favour Endo GIA™ staples to effectively manage lung parenchyma.

## Use of indocyanine green (ICG) fluorescence for diagnosis and staging

ICG has emerged as a valuable tool in paediatric surgical oncology; its ability to fluoresce under near-infrared light allows it to accurately delineate tumour margins, detect hidden metastases and facilitate lymphatic mapping intraoperatively. The technology complements conventional imaging techniques and contributes to a more precise and conservative surgery, with the potential to improve oncological and functional results in children and adolescents with solid neoplasms.

### ICG in hepatoblastoma

ICG has been used in many instances for assisting both primary tumour resection and the identification of metastases, aiding minimally invasive pulmonary metastasectomy [12].

A South Korean study involving 17 patients undergoing ICG fluorescence-guided radical hepatoblastoma resection demonstrated its precision in delineating tumour margins and its potential feasibility for detecting tumour spread and lesions not visible on conventional imaging [13].

### ICG for pulmonary metastases

A Japanese study showed that ICG facilitated intraoperative detection of pulmonary micro-metastases that were not identified by palpation or preoperative imaging. A total of 250 fluorescent nodules were resected, some as small as 62 µm, suggesting a significant improvement in detection thresholds [14].

### Identification of occult lesions

A study from China involving 16 patients revealed that the use of ICG enabled the identification of and resection of additional lesions that were not detected in preoperative imaging, achieving negative surgical margins in all cases [15].

### Sentinel lymph node mapping with ICG

A prospective study evaluated the use of ICG in combination with technetium-99m for sentinel lymph node biopsies in paediatric and adolescent patients. ICG fluorescence demonstrated 100% sensitivity in identifying the sentinel lymph node, with no adverse events reported [16].

### Wilms tumour lymph node sampling with ICG

Lymph node sampling is a critical step in Wilms tumour nephrectomy. Recent publications have highlighted the utility of intraparenchymal injection of ICG to aid lymph node identification during MIS nephrectomy [17, 18]. A recent single-centre study of MIS nephrectomies comparing 7 patients that received ICG-guided lymph node sampling to 18 controls suggested that the technique is safe, feasible and may improve the total lymph node yield [19].

## Conclusion

MIS remains an essential tool for the diagnosis and staging of paediatric solid tumours. Its role will likely evolve for procedures that once mandated an open approach, such as RPLND, enhanced by newer robotic and image-guided techniques, such as ICG fluorescence. Its current mainstay is that of a valuable option should the more common USCNB be unavailable or impossible in challenging scenarios.

## Conflicts of interest

None to declare.

## Funding

No specific funding received.

## References

[1] Paraboschi I, Bolognesi E, Giannettoni A (2021). The role of biopsy in the workup of patients with neuroblastoma: comparison of the incidence of surgical complications and the diagnostic reliability of diverse techniques. Children.

[2] Uwaydah NI, Jones A, Elkaissi M (2014). Pediatric robot-assisted laparoscopic radical adrenalectomy and lymph-node dissection for neuroblastoma in a 15-month-old. J Robot Surg.

[3] Riccipetitoni G, Bertozzi M, Gazzaneo M (2021). The role of video-assisted thoracoscopic surgery in pediatric oncology: single-center experience and review of the literature. Front Pediatr.

[4] Jackson TJ, Williams RD, Brok J (2019). The diagnostic accuracy and clinical utility of pediatric renal tumor biopsy: report of the UK experience in the SIOP UK WT 2001 trial. Pediatr Blood Cancer.

[5] Irtan S, Jitlal M, Bate J (2015). Risk factors for local recurrence in Wilms tumour and the potential influence of biopsy - the United Kingdom experience. Eur J Cancer.

[6] Weldon CB, Madenci AL, Tiao GM (2020). Evaluation of the diagnostic biopsy approach for children with hepatoblastoma: a report from the children’s oncology group AHEP 0731 liver tumor committee. J Pediatr Surg.

[7] Tomaszewski JJ, Sweeney DD, Kavoussi LR (2010). Laparoscopic retroperitoneal lymph node dissection for high-risk pediatric patients with paratesticular rhabdomyosarcoma. J Endourol.

[8] El-Gohary Y, Mansfield S, Talbot L (2020). Single-site retroperitoneoscopy in pediatric metastatic lymphadenopathy. J Pediatr Surg.

[9] Cost NG, DaJusta DG, Granberg CF (2012). Robot-assisted laparoscopic retroperitoneal lymph node dissection in an adolescent population. J Endourol.

[10] Warmann SW, Lieber J, Schaefer JF (2023). Thoracoscopic resection of lung nodules following CT-guided labeling in children and adolescents with solid tumors. Children.

[11] Lautz TB, Farooqui Z, Jenkins T (2021). Thoracoscopy vs thoracotomy for the management of metastatic osteosarcoma: a Pediatric Surgical Oncology Research Collaborative Study. Int J Cancer.

[12] Hiyama E (2021). Fluorescence image-guided navigation surgery using indocyanine green for hepatoblastoma. Child (Basel, Switzerland).

[13] Cho YJ, Namgoong JM, Kwon HH (2021). The advantages of indocyanine green fluorescence imaging in detecting and treating pediatric hepatoblastoma: a preliminary experience. Front Pediatr.

[14] Yamada Y, Ohno M, Fujino A (2019). Fluorescence-guided surgery for hepatoblastoma with indocyanine green. Cancers (Basel).

[15] Shen Y, Zheng M, Li J (2022). Clinical application of indocyanine green fluorescence imaging in the resection of hepatoblastoma: a single institution’s experiences. Front Surg.

[16] Pio L, Richard C, Zaghloul T (2024). Sentinel lymph node mapping with Indocyanine green fluorescence (ICG) for pediatric and adolescent tumors: a prospective observational study. Sci Rep.

[17] Abdelhafeez AH, Davidoff AM, Murphy AJ (2022). Fluorescence-guided lymph node sampling is feasible during up-front or delayed nephrectomy for Wilms tumor. J Pediatr Surg.

[18] Pachl MJ (2021). Fluorescent guided lymph node harvest in laparoscopic Wilms nephroureterectomy. Urology.

[19] Roberts R, Pachl M (2024). Intraparenchymal indocyanine green use improves nodal yield during minimally invasive tumor nephrectomy in children. J Laparoendosc Adv Surg Tech A.

# Other: Minimally invasive surgery in neurogenic tumours

**DOI:** 10.3332/ecancer.2031

**Published:** 2025-10-23

**Authors:** Samer Michael, J Ted Gerstle

**Affiliations:** 1 Department of Paediatric Surgery, Hadassah University Medical Center, Kiryat Hadassah, Jerusalem, Israel; 2 Paediatric Surgery Service, Memorial Sloan Kettering Cancer Center, 1275 York Avenue, New York, NY, USA; 3 Faculty of Medicine, The Hebrew University of Jerusalem, Jerusalem, Israel

**Keywords:** neurogenic, neuroblastoma, minimally invasive surgery

## Abstract

The use of minimally invasive surgery (MIS) for the diagnosis and treatment of neurogenic tumours has markedly increased over the past decade, evolving from a diagnostic and staging tool to a therapeutic option in carefully selected cases. The advantages of MIS—reduced postoperative pain, shorter hospital stay, and faster recovery—must be weighed against its technical challenges, including limited operative space, loss of tactile feedback and increased risk when image-defined risk factors are present. This chapter reviews current evidence, outlines practical indications and contraindications and proposes structured guidelines for MIS in paediatric neurogenic tumours to assist surgeons in safe adoption while maintaining oncologic integrity.

## Introduction

The use of Minimally Invasive Surgery (MIS) for the diagnosis and treatment of neurogenic tumors has markedly increased over the past decade, ranging from biopsy and staging to definitive resection. [1, 2].

Although there are no prospective studies nor randomised trials [3, 4], several retrospective studies and systematic reviews were conducted to compare the safety and outcomes of MIS and open surgery.

MIS in paediatric surgery in general and in oncologic surgery in particular has unique challenges. Patients have smaller bodies, restricting space for large tumours, the need for a larger incision for extraction, difficulty in navigation or limited room for stapler, use of adequate instrumentation and anaesthesia difficulties. Additionally, due to the lower number of cases, it is more difficult to gain experience and the learning curve might be an issue [5]. Moreover, the larger size and vascular encasement in neurogenic tumours pose an additional challenge for MIS.

### Advantages and disadvantages of MIS

The advantages of MIS under the correct indications are similar to other fields and include reduced postoperative pain, lower incidence of ileus, shorter hospital stays and earlier return to activity [1].

Although not statistically significant, Phelps *et al* [6] found a trend toward earlier initiation of adjuvant chemotherapy after MIS resection of embryonal tumours, including neuroblastoma.

The use of MIS for tumour biopsy is supported by several studies to be safe and feasible [7–13].

Despite the potential advantages of MIS, neurogenic tumours are technically and ontologically more challenging to resect than other diseases. From a technical standpoint, the tumours are often large and difficult to mobilise in a small space with reduced visibility and lacking tactile feedback. In addition, the ability to control injury to encased vessels is reduced [1, 6].

From an oncological standpoint, the ability to achieve complete macroscopic resection in locally advanced tumours is difficult, risky and requires exceptional MIS skills and experience.

### Feasibility, safety and outcomes

In a study of oncologic integrity and safety of MIS versus open surgery [6], 101 patients (50%) had neuroblastoma, of which, 20 patients underwent MIS.

MIS was correlated with older age, greater body surface area, stage I and stage II tumours, smaller tumour volume and no image-defined risk factors (IDRFs).

Almost all patients undergoing MIS resection had ≥98% tumour resection based on measurement of residual tumour on postoperative imaging, which may be attributed to the lower number of IDRFs and the greater proportion of upfront resections in this group

Relapse-free survival and overall survival (OS) did not differ significantly between the two approaches after stratification by stage and risk category.

In a study of the National Cancer Data Base from 2010 to 2012 [9], 17% (98 of 579) of children with neuroblastoma underwent MIS: patients were more likely to have a thoracic tumor, smaller size, and no metastatic disease.

After propensity score matching, there were 196 children undergoing open surgery compared with 98 children undergoing MIS. There was no difference between open and MIS surgery for 30-day mortality, readmissions, surgical margin status and 1- and 3-year survival. Median hospital length of stay was shorter in MIS versus open surgery (3 versus 4 days; *p* < 0.01). Among MIS cases, 7% were robotic assisted and 12.2% were converted to open.

In 2022, A SIOPEN multicentre study [12] reviewed 222 cases of MIS resection for neurogenic tumours. 54% were adrenal lesions, followed by 35% thoracic tumours. 73.4% had no IDRFs. A conventional laparoscopic approach was used in the majority of abdominal and pelvic tumours, 11 patients had retroperitoneoscopic resection and 1 had robotic surgery.

The number of ports for both thoracoscopic and laparoscopic procedures was 3–4, with no reported port site recurrences. The median duration for surgery was 120 minutes (range = 40–530 minutes). Resection of lymph nodes, in addition to the primary, was performed in 32 patients (14.4%).

The surgery was converted to open in 23 patients (10%) due to significant scarring, fibrosis, invasion of renal pedicle or tumour encasement of major vessels in the chest or the abdomen.

Reported complications included significant blood loss, Horner's syndrome, chylothorax and renal atrophy. The overall 30-day postoperative complication rate for all patients was 10% (*n* = 21) (9 Grade I, 6 Grade II and 6 Grade III according to Clavien-Dindo grading system).

Univariate analysis showed that thoracic procedures, presence of IDRF and tumour volume >60 mL were the main significant risk factors for open conversion.

Reasons for open conversion notably include close proximity of tumour to major blood vessels, intercostal disease infiltration and significant intra operative bleeding.

Volume >75 mL was significantly associated with complications. The tumour volume, however, did not modify the extent of resection or the recurrence rate.

Thoracic procedures were found to be associated with a higher rate of complications and increased risk of incomplete macroscopic resection compared to abdominal operations.

L2 staging and consecutively the presence of one or more than one IDRF were statistically significant factors for conversion to open surgery, incomplete resection, development of complications and recurrence of the tumour.

On further multivariate analysis, the presence of more than one IDRF remained a statistically significant risk to conversion to open. L2 stage remained a risk for complications but not for conversion, incomplete resection or recurrence. Tumour volume above 100 mL was a risk factor for conversion to open and development of complications, while MYCN amplification was a risk factor for incomplete resection.

### Indications and contraindications

MIS is typically used in smaller volume and earlier stage neuroblastic tumours without IDRFs [6, 7, 14, 15]. The emphasis on patient selection according to size and IDRFs is supported by several studies. Laparoscopic resection of abdominal and adrenal neuroblastomas was performed on tumours ≤60 mm in diameter with no IDRFs, with a low complication rate and outcomes comparable to open surgery [7, 15].

The surgeon should also consider the subtotal encasement and deformation of major vessels as a contraindication to MIS [16].

In a systematic review by the American Paediatric Surgical Association Cancer committee in 2020 [4], the authors concluded that abdominal MIS is feasible and can be safely performed for carefully selected tumours with acceptable morbidity. Conclusions could not be drawn regarding disease-free or OS outcomes. The absence of IDRFs appears to be a safe criterion for the utility of MIS. Complications and conversion to open procedures are more common in the presence of IDRFs, and for these patients, open resection may be preferable. They also concluded that optimal tumour size ranges from 4 to 6 cm without IDRF's, with a weak evidence level.

Regarding thoracic MIS, they concluded that it seems feasible, safe and effective. With reported complications comparable to thoracotomy. Thoracic neurogenic tumours may be more amenable to MIS resection in the absence of IDRF's.

Although the size of the primary tumour as a criterion for MIS resection of thoracic neuroblastic tumours has not been as extensively studied, in their review, they found that tumour size ranged between 2  and 18 cm and the most commonly reported sizes were between 3 and 5 cm.

They found comparable rates of complications compared to the open approach and are likely more attributable to tumour location rather than method of resection. However, it should be noted that tumours undergoing MIS resection in the reported studies were highly selected, which may explain improved morbidity.

The authors emphasised that the use of MIS techniques on inappropriate patients or by surgeons with limited MIS skills could have detrimental outcomes. Patients should be highly selected, mainly based on size and the presence of IDRF's.

In 2010, The International Paediatric Endosurgery Group published guidelines (class III evidence) for the surgical management of adrenal masses in children [17].

The consensus suggested that for advanced neuroblastoma, laparoscopic biopsy may be performed, and that laparoscopic resection of small, localised tumours may be performed as long as the principles of oncologic surgery are maintained. MIS can also be considered for higher risk disease if the tumour responds favourably to neoadjuvant chemotherapy. A strong recommendation was made by this group to remove all tumours using an endoscopic retrieval bag.

The use of ICG during MIS for neuroblastic tumours has been described; however, further research is required to establish its benefits [18].

In our experience, the use of a number of IDRFs to predict surgical complexity is limited and should be used with caution. Alternatively, we recommend using the following terms and definitions to describe the relationship of the tumour to any adjacent critical structure [19]:

- Contact: no visible layer is present between the tumour and adjacent structure <50% of the vessel's circumference (not an IDRF).
- The term flattened is used to describe veins with reduced diameter that still have a partially visible lumen (not an IDRF).
- Encasement for a vessel means >50% of the vessel circumference is in contact with tumour (IDRF).
- Total encasement means that a vital structure is completely surrounded by tumour (IDRF).
- A flattened vein with no visible lumen is considered to be encased (IDRF).
- Compression is used only when referring to airways. When tumour contacts an airway and causes the short axis to be reduced, this is considered an IDRF.
- For other vital structures, a contact may cause displacement (abnormal anatomic location) or distortion (abnormal anatomic shape) (not IDRF).

In addition to the circumference of contact with the vessel, the length of contact should also be noted, for longer contact increases the risk for vessel injury, which is more difficult to manage in MIS.

In retroperitoneal and pelvic tumours, the contact and encasement of the ureter should also be taken into consideration in the decision on MIS suitability.

We highly recommend using intraoperative neurophysiological monitoring (IONM) in thoracic tumours with involvement of intervertebral foramina or tumour with proximity to pelvic nerves.

A summary of suggested guidelines and practical recommendations for patient selection and surgical approach is provided in Table 1.

### Robotic-asssisted MIS (RA-MIS)

Although it potentially has technical advantages in precision and maneuvering, it is not surprising that robotic MIS faces more challenges in paediatric oncology. A multicentre review of RA-MIS in paediatric cancer included 22 thoracic and abdominal neuroblastic tumours [20]. They showed that it is technically feasible and safe to perform in select cases. Providing the benefits of MIS, such as reduced length of stay and improved cosmesis, robotic-assisted surgery also provides the added benefits of improved dexterity and enhanced visualisation, including three-dimensionality.

The high-volume Paediatric Robotic Program at Hôpital Necker-Enfants malades (Paris) reported robotic resection of 51 neurogenic tumours in 47 children; 57% adrenal and 19% posterior mediastinum. 6 (11%) patients had vascular encasement (3 renal pedicle, 2 iliac vessel and 1 vena cava encasement).

They reported one conversion and no emergency undocking. Resection was macroscopically complete in all cases (confirmed on post-operative imaging).

There were no cases of vascular injury or post-operative complications [10].

It is important to note that these results should be interpreted cautiously, since the surgeries were performed in a high-volume paediatric robotic surgery centre and 89% of patients had no IDRFs.

### Surgical approach

#### Thoracoscopic resection

##### 1. Patient positioning

- Use an appropriate size, use a bean bag or other supportive devices to stabilise the patient.
- Patient needs to lie on foam or gel pads to minimise pressure injury risks.
- Place the patient in a 45-degree semi-prone position with the tumour side up, for gravity to drop the lungs away from the spine.
- The operating table should be slightly flexed to widen the intercostal spaces and improve thoracic access.
- In younger patients, the beanbag can be used for flexion.
- The patient should be as close as possible to the edge of the table towards the operator.
- The operator should stand anterior to the patient.

Arms are positioned to allow access without obstruction while protecting from neuropraxia [9–13].

##### 2. Trocar sites

- Use a triangulation approach for optimal visualisation and instrument maneuverability:
  1. Camera port:
    - Typically placed in the midaxillary line in the 5th or 6th intercostal space.
  2. Working port 1:
    - Typically placed 2–3 cm anterior to the midaxillary line, in the 4th or 5th intercostal space.
  3. Working port 2:
    - Placed posterior to the midaxillary line above the costophrenic junction (to allow enough length for the stapler).
- Ports should be placed under vision to avoid damage to major vessels, lungs or diaphragm.
- Additional ports (should be used as needed).

##### 3. Surgical technique

- Use a lower insufflation pressure (6–8 mmHg) to minimise cardiovascular and respiratory issues.
- Tumour dissection should proceed from the peripheral to the central area and saving division of critical structures until the tumour has been isolated.
- Identify critical structures to avoid injury.
- Use Endo Catch bag for tumour retrieval.

##### 4. Tips and pitfalls

Tips:

- Memorise preoperative imaging and relative location of vascular and other vital structures.
- Display imaging during procedure for frequent reference.
- Go over the rescue plan for an intraoperative bleeding with the team before the start of procedure.
- Patient safety and oncologic principles should be prioritised with a low threshold to convert to open surgery.
- We highly recommend using IONM in thoracic tumours with involvement of intervertebral foramina.

Pitfalls:

- The use of MIS should not compromise the oncologic principles of resection, sampling and reporting of operative findings, according to other most recent oncology groups' guidelines.
- Resection extent should not be sacrificed in favour of a minimally invasive approach.
- Inappropriate patient selection.
- OR team members, including anaesthesia and surgery teams not experienced with paediatric MIS.
- Not positioning patient appropriately.
- Tissue planes are difficult after neoadjuvant therapy.
- Not recognising feeding and draining vessels.

#### Laparoscopic adrenalectomy/retroperitoneal tumour resection

##### 1. Patient position

- Place the patient in a lateral decubitus position, with the tumour side up.
- Use an appropriate size, use a bean bag or other supportive devices to stabilise the patient.
- The operating table should be slightly flexed at the lumbar region to open up the retroperitoneal space and improve access.
- Patient needs to lie on foam or gel pads to minimise pressure injury risks.
- In younger patients, the beanbag can be used for flexion.
- The patient should be as close as possible to the edge of the table towards the operator.
- The operator should stand anterior to the patient.
- Arms are positioned to allow access without obstruction while protecting from neuropraxia.

##### 2. Trocar sites

- Use a triangulation approach for optimal visualisation and instrument maneuverability:
  1. Camera port:
    - Typically placed at the umbilicus.
  2. Working port 1:
    - Mid-clavicular line, about 2–4 cm below the costal margin.
  3. Working port 2:
    - Anterior axillary line, about 2–4 cm below the costal margin.
  4. Accessory port (optional):
    - Positioned in the epigastric region or lower quadrant, as needed, for retraction or stapling.

##### 3. Surgical technique

- Maintain a low insufflation pressure (8–10 mmHg) to avoid compromising ventilation or venous return in small paediatric patients.
- Mobilise the tumour or adrenal gland working medially from the lateral border.
- Identify critical structures to avoid injury.
- Identify and isolate the adrenal vein early in the procedure.
- For the right adrenal gland, locate the adrenal vein draining into the inferior vena cava.
- For the left adrenal gland, identify the vein draining into the left renal vein.
- Secure the adrenal vein with clips or an energy-sealing device before transection.
- Use Endo Catch bag for tumour retrieval.

##### 4. Tips and pitfalls

Tips:

- Memorise preoperative imaging and relative location of vascular and other vital structures.
- Display imaging during procedure for frequent reference.
- Go over the rescue plan for an intraoperative bleeding with the team before the start of procedure.
- Patient safety and oncologic principles should be prioritised with low threshold to convert to open surgery.

Pitfalls:

- The use of MIS should not compromise the oncologic principles of resection, sampling and reporting of operative findings, according to other most recent oncology groups guidelines.
- Resection extent should not be sacrificed in favour of a minimally invasive approach.
- Inappropriate patient selection.
- OR team members including anaesthesia and surgery teams not experienced with paediatric MIS.
- Not positioning the patient appropriately.
- Tissue planes are difficult after neoadjuvant therapy.
- Not recognising feeding and draining vessels.
- Secure vessels early to prevent uncontrolled bleeding.
- Adrenal vein misidentification: Mistaking the adrenal vein for surrounding structures (e.g., renal vein or inferior vena cava) can lead to complications.
- Instrument crowding: Paediatric patients’ small abdominal cavities can limit space for instruments; plan trocar placement carefully.

#### Laparoscopic pelvic tumour resection

##### 1. Patient position

- Patient in supine position.
- Elevate the pelvis slightly using a gel roll to improve access to the presacral space.
- Adjust the table to a Trendelenburg tilt to allow the bowel to fall cranially, enhancing visualisation of the pelvis.
- Arms are secured and padded to prevent nerve injury.

##### 2. Trocar sites

- Use a triangulation approach to provide optimal visualisation and maneuverability:
  - Camera port:
    Positioned at or just above the umbilicus.
  - Working port 1:
    Placed in the right lower quadrant, at the midclavicular line, about 2–3 cm above the anterior superior iliac spine.
  - Working port 2:
    Placed in the left lower quadrant, symmetric to the first working port.
  - Accessory port (optional):
    Positioned in the suprapubic region for retraction or additional instrumentation, if necessary.

##### 3. Surgical technique

1. Maintain a low insufflation pressure (8–10 mmHg) to avoid compromising ventilation or venous return in small paediatric patients.
2. Pelvic neurogenic tumours should have a clear plane separating them from the rectum and colon mesentery, which should be followed for mobilisation.
3. The ureter is usually pushed away by the tumour and should be dissected off bluntly with caution.
4. Identify critical structures such as the sacral plexus, iliac vessels and ureters to avoid injury.
5. Place the tumour in an Endo Catch bag for extraction through the largest port or a mini-incision.

##### 4. Tips and pitfalls

Tips:

- Memorise preoperative imaging and relative location of vascular and other vital structures.
- Display imaging during procedure for frequent reference.
- Go over the rescue plan for an intraoperative bleeding with the team before the start of procedure.
- Patient safety and oncologic principles should be prioritised with low threshold to convert to open surgery.
- Ensure the urinary bladder and rectum are empty before surgery to reduce risk of injury and improve visualisation.
- We highly recommend using IONM in tumours with proximity to pelvic nerves.

Pitfalls:

- The use of MIS should not compromise the oncologic principles of resection, sampling and reporting of operative findings, according to other most recent oncology groups guidelines.
- Resection extent should not be sacrificed in favour of a minimally invasive approach.
- Inappropriate patient selection.
- OR team members, including anaesthesia and surgery teams not experienced with paediatric MIS.
- Not positioning patient appropriately.
- Tissue planes are difficult after neoadjuvant therapy.
- Not recognising feeding and draining vessels.
- Secure vessels early to prevent uncontrolled bleeding.
- Instrument crowding: Paediatric patients’ small abdominal cavities can limit space for instruments; plan trocar placement carefully.

## Conclusion

MIS is a safe and effective option for selected paediatric neurogenic tumours, particularly smaller, localised cases without IDRFs. While it offers benefits like reduced pain and faster recovery, its success depends on careful patient selection, adherence to oncologic principles and surgical expertise. MIS should only be attempted in appropriate cases, with a low threshold for conversion to open surgery to ensure patient safety and optimal outcomes. Further research is needed to strengthen evidence and refine guidelines.

## List of abbreviations

IDRFs, Image-defined risk factors; IONM, Intra-operative neural monitoring; MIS, Minimally invasive surgery; RA-MIS, Robotic-assisted MIS; SMA, Superior mesenteric artery; SVC, Superior vena cava.

## Conflicts of interest

The authors declare that they have no conflicts of interest.

## Funding

The authors declare that they have no financial conflicts of interest.

## Figures and Tables

**Table 1.** Suggested Guidelines and Recommendations

	May consider MIS	Relative contraindications	Absolute contraindications	Notes
All sites	Diagnostic biopsy Small, localised tumour without IDRFs Upfront resection without neoadjuvant chemotherapy	Contact with large vessel Involvement of intervertebral foramina Tumour volume 60–75 mL s/p Neoadjuvant chemotherapy	One or more IDRF Significant Contact (length, % circumference flattening) with large vessel Intra spinal involvement >75 mL Advanced stage neuroblastoma Invasion to adjacent organ Tumour occupying most of the cavity or causing significant displacement or distortion of adjacent structures. Tumour size in relation to patient size expected to have limiting space impairing proper visualisation and safe dissection Lesion is a metastatic node	Consider observation only when clinically indicated. Multidisciplinary tumour board discussion is strongly advised to agree on suitability of MIS. Use Endo Catch bag for extraction in tumour resections. The use of MIS should not undermine adherence to oncologic principles of resection and reporting of operative findings. according to other most recent oncology groups guidelines The rescue plan for an intraoperative bleeding should be prepared by the operating surgeon. Low threshold to conversion to open surgery. Evaluate the risk of any anaesthetic complications caused by mass effect together with the anaesthesiology team prior to deciding the optimal procedure, which must be individualised to each clinical scenario.
Thoracic	Paraspinal tumour Tumour <6 cm or 60 mL		Involving thoracic inlet or apex Significant contact with aorta, subclavian vessels, superior vena cava, main bronchus, trachea, esophagus and pericardium Tumour crossing midline. Mass is more than one third of the thorax. Left sided tumour located in T9–T12 (risk for the Adamkiewicz artery)	Single lung ventilation is often required to provide adequate operating space
Adrenal	Small, localised tumour ‘simple adrenalectomy’		Suspected adrenocortical carcinoma Tumour crossing midline. Pancreatic involvement Significant contact with IVC, renal pedicle, aorta, porta hepatis, superior mesenteric artery (SMA) and Celiac trunk Lymph node involvement	Either transperitoneal or retroperitoneal laparoscopy can be employed depending on surgeon’s experience
Abdominal non-adrenal	Small, localised tumour		Significant contact with IVC, renal pedicle, aorta, porta hepatis, SMA and celiac trunk Lymph node involvement Tumour crossing midline. Any renal hilum or pancreatic involvement Significant contact with the ureter	Either transperitoneal or retroperitoneal laparoscopy can be employed depending on surgeon’s experience
Pelvis	Pre sacral		Extension into intervertebral foramina Significant contact with Iliac vessels or nerve plexus Significant contact with the ureter	IONM recommended in tumours with lateral extension

## References

[1] Phelps H, Lovvorn I (2018). Minimally invasive surgery in pediatric surgical oncology. Children.

[2] Spencer B, Patel A, Cilley R (2022). Surgical management in pediatric neuroblastoma diagnosis and treatment: a 20-year, single-center experience. World J Pediatr WJP.

[3] Van Dalen EC, De Lijster MS, Leijssen LG (2015). Minimally invasive surgery versus open surgery for the treatment of solid abdominal and thoracic neoplasms in children. Cochrane Database Syst Rev.

[4] Gurria JP, Malek MM, Heaton TE (2020). Minimally invasive surgery for abdominal and thoracic neuroblastic tumors: a systematic review by the APSA cancer committee. J Pediatr Surg.

[5] Galazka P, Czyzewski K, Marjanska A (2019). Minimally invasive surgery in pediatric oncology: proposal of guidelines. Anticancer Res.

[6] Phelps HM, Ayers GD, Ndolo JM (2018). Maintaining oncologic integrity with minimally invasive resection of pediatric embryonal tumors. Surgery.

[7] Zenitani M, Yoshida M, Matsumoto S (2023). Feasibility and safety of laparoscopic tumor resection in children with abdominal neuroblastomas. Pediatr Surg Int.

[8] Kawano T, Souzaki R, Sumida W (2022). Laparoscopic approach for abdominal neuroblastoma in Japan: results from nationwide multicenter survey. Surg Endosc.

[9] Ezekian B, Englum BR, Gulack BC (2018). Comparing oncologic outcomes after minimally invasive and open surgery for pediatric neuroblastoma and Wilms tumor. Pediatr Blood Cancer.

[10] Blanc T, Taghavi K, Glenisson M (2024). Robotic surgery in paediatric oncology: expanding boundaries and defining relevant indications. J Pediatr Surg.

[11] Abib SDCV, Chui CH, Cox S (2022). International Society of Paediatric Surgical Oncology (IPSO) surgical practice guidelines. ecancermedicalscience.

[12] HO G (2022). Minimally invasive surgery for neuroblastic tumours: a SIOPEN multicentre study: proposal for guidelines. Eur J Surg Oncol.

[13] Pachl M, Lautz TB, Aldrink JH (2024). Minimally invasive and robotic‐assisted approaches applied to pediatric surgical oncology. Pediatr Blood Cancer.

[14] Shirota C, Tainaka T, Uchida H (2017). Laparoscopic resection of neuroblastomas in low- to high-risk patients without image-defined risk factors is safe and feasible. BMC Pediatr.

[15] Oesterreich R, Varela MF, Moldes J (2022). Laparoscopic approach of pediatric adrenal tumors. Pediatr Surg Int.

[16] Tanaka Y, Kawashima H, Mori M (2016). Contraindications and image-defined risk factors in laparoscopic resection of abdominal neuroblastoma. Pediatr Surg Int.

[17] IPEG guidelines for the surgical treatment of adrenal masses in children. J Laparoendosc Adv Surg Tech A.

[18] AC H (2023). Early results of minimally invasive fluorescent guided pediatric oncology surgery with delivery of indocyanine green during induction of anesthesia. Photodiagnosis Photodyn Ther.

[19] Brisse HJ, McCarville MB, Granata C (2011). Guidelines for imaging and staging of neuroblastic tumors: consensus report from the International Neuroblastoma Risk Group Project. Radiology.

[20] Svetanoff WJ, Carter M, Diefenbach KA (2024). Robotic-assisted pediatric thoracic and abdominal tumor resection: an initial multi-center review. J Pediatr Surg.

# Other: Minimally invasive surgery in paediatric nephroblastoma

**DOI:** 10.3332/ecancer.2024

**Published:** 2025-10-23

**Authors:** Steven W Warmann, Jörg Fuchs

**Affiliations:** 1 Department of Paediatric Surgery and Paediatric Urology, Charité University Hospital, Berlin, Germany; 2 Department of Paediatric Surgery and Paediatric Urology, University Children’s Hospital, Tübingen, Germany

**Keywords:** nephroblastoma, minimally invasive surgery, tumour nephrectomy

## Abstract

Surgery is a corner stone of treatment in children with nephroblastoma. Over recent years, the evolution of surgical guidelines has added to the improvement of treatment results in affected children. Minimally invasive surgery (MIS) has been described as part of this evolution. The present article summarises the current recommendations for MIS in nephroblastoma based on the relevant global treatment protocols.

## Introduction

Minimally invasive surgery (MIS) has gained increasing importance in all fields of paediatric surgery, including surgical oncology. More and more treatment protocols for paediatric solid tumours include guidelines on MIS [1–3]. MIS has been used in paediatric urology relatively early, consecutively, renal tumours came into the focus of surgeons in this regard. As a consequence, historical reports on MIS for paediatric renal tumours occurred fairly early [4, 5]. The surgical panel of the SIOP Renal Tumour Study Group (SIOP RTSG) has for the first time systematically evaluated the results of MIS in paediatric nephroblastoma and has formulated guidelines, which are part of the current SIOP RTSG Umbrella protocol [6]. Meanwhile, there is a growing experience with this subject [7–9]. Surgical results and oncological outcomes of MIS are similar to the results obtained by open surgery; however, lymph node sampling is often insufficient when MIS is used [8, 10].

The use of robotic surgery has recently been introduced in children with nephroblastoma. Preliminary results indicate its potentially favorable contribution; however, the definitive role of this technique and its possible addition to treatment protocols still have to be clarified. Specific respective surgical guidelines seem necessary [11–13].

Taken together, MIS has been established as a safe and successful technique for resection of nephroblastoma provided there is careful patient selection, and surgeons have sufficient expertise in the fields of MIS, oncologic surgery and paediatric urology. Irrespective of the specific technique that is applied, the oncological principles of Wilms Tumour surgery have to be strictly respected.

## Indications and contraindications

Based on the experiences of the SIOP 2001 study, the SIOP RTSG formulated the following indications and contraindications for MIS in nephroblastoma in the current Umbrella Protocol [6, 14].

### Indications:

1. Resection must adhere to oncological principles and include lymph node sampling.
2. Small, central tumours with a rim of ‘normal’ renal tissue.
3. The extraction of the specimen in a bag, without morcellation, through an adequate abdominal wall incision, is mandatory, not only to control the risk of dissemination, but also to ensure adequate histopathological staging.
4. If nephron-sparing surgery (NSS) is feasible, it should be preferred even if an open approach is needed.

### Contraindications:

1. Tumour infiltrating extra-renal structures or extending beyond the ipsilateral border of the spinal column.
2. Thrombus in the renal vein or vena cava.
3. Peripheral location if NSS is not deemed feasible.
4. Tumour without any response to chemotherapy due to the risk of tumour rupture.
5. Little or no experience in laparoscopic nephrectomy (consider transfer to another unit or obtain more experienced help).

According to the SIOP Umbrella protocol, neoadjuvant chemotherapy is a precondition for MIS. Provided there is a careful patient selection, MIS for nephroblastoma is associated with comparable results as the open approach regarding complications, surgical results, and oncological outcome. Current analyses imply that not every child with Wilms Tumour is per se a candidate for MIS. This holds even more true if upfront surgery is recommended as the primary approach, as is, for example, the case for certain conditions in the protocols of the US-based Children’s Oncology Group.

The SIOP RTSG guidelines for MIS in children were introduced in a rather conservative manner, with the main focus on their applicability and safety. Since their introduction, some authors have reported that experienced surgeons may address some of these aspects with a broader view concerning indications [15].

Minimally invasive NSS for nephroblastoma has not been systematically analysed so far. There exist some reports on singular cases. Authors in this regard regularly formulate the necessity for caution and the need to further analyse this approach before its advocation on a larger scale [16, 17].

## Surgical approach – minimally invasive tumour nephrectomy

Currently, the surgical approach of MIS in nephroblastoma can be divided into three groups: transperitoneal laparoscopic, retroperitoneal or robotic. To date, there exists no systematical analyses comparing these three MIS approaches. The largest number of authors reported on the usage of the transperitoneal approach. According to the Umbrella protocol of the SIOP RTSG, children with nephroblastoma have to receive neoadjuvant chemotherapy before undergoing MIS. Concerning patients' age, MIS can be executed safely after chemotherapy, irrespective of the age and size of the children.

For the laparoscopic approach, usually a three- or four-trocar technique is used with the children being positioned in a supine or an angled decubitus position [8, 9]. Mobilisation of the colon on the affected side is followed by mobilising of the organ and exploration of the vessels. This step can relevantly be facilitated by using a transabdominal traction suture through the proximal ureter to elevate the medial plane of the kidney and the renal hilus. The rule of transsecting the renal artery before the vein applies to MIS identically. Lymph node sampling should be performed in accordance with the guidelines of open surgery. In some cases, it can be preferable to perform lymph node sampling before dividing the renal vessels. The reason for this is the potential retraction of midline structures after vascular transection, through which identification of lymph nodes and regions of their origin might become more difficult. The ureter should be divided as closely as possible to the urinary bladder. After complete mobilisation, the resected specimen should be removed from the situs without morcellation, preferably using a retrieval bag. Most authors advocate a Pfannenstiel incision for the removal of the specimen.

Slight alterations of the approach have been reported when using the retroperitoneal approach. These alterations mainly concern a larger angle for patient positioning, positioning of trocars, establishment of a retroperitoneal space, and exploration of the targeted organs [18, 19].

In conclusion, MIS for paediatric nephroblastoma is an established technique with a distinct role in the surgical treatment of affected patients. Patient selection and surgical expertise are the most important factors to grant results that are comparable with those of open surgical procedures. The role of minimally invasive NSS and robotic surgery yet needs to be further clarified.

## Conflicts of interest

The authors have no conflicts of interest to report.

## Funding

This manuscript has been written with no funding involved.

## References

[1] Fuchs J, Schafbuch L, Ebinger M (2014). Minimally invasive surgery for pediatric tumors - current state of the art. Front Pediatr.

[2] Pachl M, Lautz TB, Aldrink JH (2024). Minimally invasive and robotic-assisted approaches applied to pediatric surgical oncology. Pediatr Blood Cancer.

[3] Gabra HO, Irtan S, Cross K (2022). Minimally invasive surgery for neuroblastic tumours: a SIOPEN multicentre study: proposal for guidelines. Eur J Surg Oncol.

[4] Duarte RJ, Dénes FT, Cristofani LM (2004). Laparoscopic nephrectomy for Wilms tumor after chemotherapy: initial experience. J Urol.

[5] Duarte RJ, Dénes FT, Cristofani LM (2006). Further experience with laparoscopic nephrectomy for Wilms' tumour after chemotherapy. BJU Int.

[6] Warmann SW, Godzinski J, Tinteren H (2014). Minimally invasive nephrectomy for Wilms tumors in children - data from SIOP 2001. Pediatr Blood Cancer.

[7] Schmidt A, Warmann SW, Urla C (2019). Patient selection and technical aspects for laparoscopic nephrectomy in Wilms tumor. Surg Oncol.

[8] Bouty A, Blanc T, Leclair MD (2020). Minimally invasive surgery for unilateral Wilms tumors: multicenter retrospective analysis of 50 transperitoneal laparoscopic total nephrectomies. Pediatr Blood Cancer.

[9] Varlet F, Petit T, Leclair MD (2014). Laparoscopic treatment of renal cancer in children: a multicentric study and review of oncologic and surgical complications. J Pediatr Urol.

[10] Mentessidou A, Djendov F, Long AM (2024). Systematic review and meta-analysis of laparoscopic versus open radical nephrectomy for paediatric renal tumors with focus on Wilms' tumor. Ann Surg.

[11] Blanc T, Pio L, Clermidi P (2019). Robotic-assisted laparoscopic management of renal tumors in children: preliminary results. Pediatr Blood Cancer.

[12] Li P, Tao Y, Zhao Y (2024). Robotic-assisted laparoscopic surgery for the treatment of Wilms' tumor in children: single-center experience and medium-term outcomes. J Robot Surg.

[13] Vinit N, Sarnacki S, Blanc T (2023). Robotic-assisted laparoscopy in pediatric surgical oncology: a narrative review. Transl Pediatr.

[14] Umbrella Protocol of the SIOP Renal Tumor Study Group (2016).

[15] Burnand K, Roberts A, Bouty A (2018). Laparoscopic nephrectomy for Wilms' tumor: can we expand on the current SIOP criteria?. J Pediatr Urol.

[16] Murawski M, Stefanowicz J (2023). Laparoscopic nephron-sparing surgery and laparoscopic nephrectomy for Wilms' tumor. Wideochir Inne Tech Maloinwazyjne.

[17] Chui CH, Lee AC (2011). Peritoneal metastases after laparoscopic nephron-sparing surgery for localized Wilms tumor. J Pediatr Surg.

[18] Luo Y, Zhang H, Wu Q (2024). Indocyanine green fluorescence imaging-assisted laparoscopy resection of retroperitoneal tumors in children: case report and literature review. Front Pediatr.

[19] Fuchs J (2015). The role of minimally invasive surgery in pediatric solid tumors. Pediatr Surg Int.

# Other: The role of the minimally invasive surgery in the management of paediatric liver tumours

**DOI:** 10.3332/ecancer.2025

**Published:** 2025-10-23

**Authors:** Alyssa Stetson, Gloria Gonzalez, Greg M Tiao

**Affiliations:** 1 Department of Paediatric General and Thoracic Surgery, Cincinnati Children’s Hospital Medical Center, USA; 2 Department of Paediatric General and Thoracic Surgery, Lerner College of Medicine and Case Western Reserve University Cleveland Clinic Children's Hospital, USA; 3 Department of Surgery, University of Chile

**Keywords:** paediatric, liver cancer, minimally invasive surgery

## Abstract

Minimally invasive surgical techniques are increasingly adopted for the management of hepatic masses in children. Laparoscopic liver biopsy can be used to obtain tissue diagnosis while avoiding the risks of open surgery and providing improved cosmesis. Laparoscopic or robotic liver resection has more gradually been adopted in children than in adults but can be utilized for appropriately located tumours as long as oncologic principles are maintained. Patient size is a factor when choosing whether to perform liver resection via a minimally invasive approach. Laparoscopic radiofrequency ablation offers an alternative strategy to surgery for paediatric patients with small masses or can serve as a bridge to transplant.

## Introduction

Minimally invasive surgery (MIS) has become a standard option in the treatment armamentarium for children who have conditions that require surgical intervention. Its role in paediatric oncologic conditions is evolving. In this review, we summarize how MIS is utilized in the management of paediatric hepatic tumours.

## Minimally invasive procedures for liver biopsy

### Introduction

Liver biopsy remains the gold standard for the diagnosis of hepatic pathologies, including masses [1]. Minimally invasive procedures allow for tissue diagnosis without the risks of an open approach.

### Indications

Because of the concern for malignancy, many children who present with a liver mass require biopsy [2, 3]. A biopsy is recommended for children with a liver mass that is not amenable to primary resection.

### Contraindications

Most cases of hepatocellular carcinoma (HCC) in children arise *de novo*, in which case biopsy is still warranted [2]. Nodules arising in a background of cirrhosis that are radiographically consistent with HCC should be treated as such, as biopsy carries up to a 5% risk of needle track seeding. Other possible contraindications to percutaneous biopsy include bleeding disorder, morbid obesity, vascular lesion, extrahepatic biliary obstruction and presence of cholangitis [2].

### Surgical approach

Minimally invasive modalities include percutaneous and laparoscopic approaches [3]. If feasible, percutaneous liver biopsy is the preferred technique due to its safety and simplicity, as well as reduced adhesions between the tumour and abdominal wall [4]. It can be performed using ultrasound or computed tomography guidance [5, 6]. While more invasive, laparoscopic-assisted core needle biopsies allow for visualization of the peritoneal cavity and lesion, increased sample volume and easier control of bleeding [2, 7]. Needles used for biopsy include ‘suction’ needles and ‘cutting’ needles, with the need to balance the safety and smaller size of the suction needle with the superior preservation of tissue architecture using the cutting needle [7].

### Tips and pitfalls

Recent literature indicates that open biopsy and laparoscopic biopsy have higher rates of post-procedural bleeding requiring a transfusion than percutaneous biopsy, but all three approaches carried similar rates of relapse [4]. Risk of bleeding after percutaneous biopsy ranges from 0% to 7.1% and appears to be higher in patients undergoing biopsy for oncologic purposes [8–11].

Core needle biopsy should be performed using a 15-gauge sheath through which a 16-gauge needle can be passed at least seven times and repositioned to biopsy various areas of the mass as well as normal liver parenchyma [12]. Tract embolization should be performed at the conclusion of the procedure. The biopsy tract should be included in the eventual resection specimen to minimize the risk of recurrence due to seeding of the tract [2, 12].

## MIS for liver resection

### Introduction

Laparoscopic liver resection (LLR) was first established in the adult population. It was initially utilized for benign diseases in which the liver lesion was easily amenable to resection, i.e., a wedge resection or left lateral sectionectomy [13]. The results from these early efforts reported decreased morbidity and pain, shorter length of stay (LOS), less estimated blood loss and improved cosmesis [14, 15]. As such, LLR became a viable option for benign lesions [15]. As experience with hepatic MIS grew and laparoscopic devices for transecting parenchyma improved, LLR was trialed for malignant disease [16, 17]. Subsequent studies have confirmed that, in appropriately selected patients, there are equivalent or superior oncologic outcomes between LLR and open hepatic resection [15, 18–20]. It is now recognized as an acceptable approach in select adult patients to treat HCC and colorectal liver metastases with benefits that include the avoidance of a large incision and lower rates of postoperative liver failure and ascites [21].

In the paediatric patient population, MIS hepatic surgery has been more gradually adopted. This can be attributed to the infrequent incidence of paediatric hepatic neoplasms, size limitations of paediatric patients with the attendant need for smaller instruments, learning curve for MIS procedures and start-up costs associated with introducing a MIS program [17, 22–24].

Despite these challenges, LLR is now performed at select paediatric centers for both benign and malignant lesions with studies reporting a similar postoperative complication rate to open liver resection, infrequent need to convert to open for bleeding control and overall oncologic success (Table 1) [14, 22, 23–27]. Widespread adoption remains challenging [28].

### Indications

Indications for LLR in children parallel those for open hepatectomy. Type of resection and adequacy of post-operative functional liver parenchyma overarch decision-making [25, 29].

### Contraindications

*Patient size -* Patient size is a concern due in large part to the dimensions of the current instrumentation [17, 23, 30]. Literature is limited and conflicted regarding how patient size should be incorporated into decision-making, with small series defining weight range and age [23, 25]. A critical determinant are the instruments that make parenchymal transection safer, like the laparoscopic cavitron ultrasonic surgical aspirator (CUSA) device, ultrasound and 12 mm staplers, all of which require larger ports placed with adequate distance to allow the head of the device to be used within the smaller abdominal domain of a child [23].

*Technical safety -* An important consideration for LLR is the management of bleeding [25]. In adults, approximately 6% of LLR are converted to open for bleeding with varying conversion rates based on the type and extent of resection [31–34]. The best way to manage this risk is with preoperative patient selection – the tumour anatomy needs to be favourable such that resection is possible without damaging any major structures [35]. Techniques to control bleeding laparoscopically are discussed below.

*Oncologic resection –* A LLR should be aborted if a clear margin cannot be achieved. In contrast to the common adult hepatic tumours of colorectal metastases and HCC arising in the context of chronic liver disease, hepatoblastoma and *de novo* HCC in children are large tumours, often occupying a hemi-liver, making an R0 resection challenging and reducing the number of paediatric liver tumours that are amenable to LLR [24, 25, 36, 37].

### Surgical approach

*Patient position -* Ideal patient positioning depends on the size of the child. In older children, positioning in lithotomy favourable [3, 23, 38]. The surgeon stands between the patient’s legs and the assistant stands on the patient’s left [23, 38]. Left lateral decubitus is also an option if the tumour is in the right posterior section [38]. The patient may be kept level or placed in reverse Trendelenburg to allow viscera to fall away [38].

*Trocar sites-* One 10–12 mm trocar should be placed at the umbilicus [14, 23]. Generally, in smaller patients we will place 3 additional 5 mm trocars in line or just below the level of the umbilicus, while in older patients the working ports are placed above the umbilicus [25]. Ideal placement can be assessed after visualization of the field [14, 39]. An additional port can be added for retraction of the liver [25]. Specimen extraction is typically through the larger umbilical port or through an inguinal or suprapubic incision [25, 40].

For hand-assisted or laparoscopic-assisted procedures (hybrid), an additional incision is made, which can be used to palpate and explore the liver and lyse adhesions. It should be made below the xiphoid and kept as small as possible while allowing for specimen extraction or an inguinal incision can be utilized [14, 40].

In patients with portal hypertension, the umbilical port should be placed inferior to the umbilicus or superior to the umbilicus and lateral to the linea alba to avoid a recanalized umbilical vein [38]. Alternatively, the abdomen can be entered via the Hasson technique and the recanalized umbilical vein ligated.

### Surgical technique

#### Types

Laparoscopic options include the conventional 3–5 ports, laparoscopic hand-assisted and hybrid. The hand-assisted and hybrid options allow additional access for mobilization, retraction, rapid hemostasis and extraction [14]. However, unless operating on a large child or adolescent, the hand-assisted technique is unlikely to significantly advance the technical approach to LLR in children due to the limited surgical field [17]. In adults, a complete hepatectomy via a single incision has been performed, but this has not yet been reported in children [41].

Robotic-assisted hepatic surgery using the Da Vinci robot in adults is increasingly utilized [42]. While robotic surgery offers better visualization and separation of the tumour, its high cost, need for specialized instruments and limited training opportunities for paediatric surgeons have restricted its use in children [17, 26]. In paediatrics, the robot has been used for hepatic surgery more commonly with choledochal cyst excision and hepaticojejunostomies [43, 44]. There are a few case reports of robotic hepatectomies in children [26, 27, 45].

#### Steps

After trocar placement, intraoperative ultrasound can be used to demarcate resection margins as well as vascular structures [14]. The lesser sac should be accessed and the porta hepatis encircled; the remnant of the divided umbilical vein can be used to elevate the liver during exposure of the porta hepatis [22, 38].

There are different techniques for how to proceed with dissection. Once the liver is mobilized, the right or left artery, portal vein and hepatic duct can be exposed and divided [38]. Debate exists as to the best way to approach the hepatic vein, with different techniques described below. The line of resection should be decided based on either the line of demarcation or ultrasound [38]. The first 2–3 cm of Glisson’s capsule contain no major structures, so can be incised, then the parenchyma divided using either gross stapling technique or visualized dissection technique [38]. Options for transection include stapling, the CUSA, the harmonic scalpel and LigaSure^TM^ [14, 23, 25]. Glissonean pedicles should be ligated and divided with a knot-pusher or Hem-o-lock clip [39].

Alternatively, the Glissonean pedicle approach (Takasaki maneuver) can be utilized. This method involves ligation and division of the vessels at the hepatic hilum prior to segmentectomy. The Glissonean pedicle is dissected free and the extrahepatic segmental branch of the corresponding liver segment is divided. The tertiary branches supplying the segment can then be transected through a hilar or parenchymal approach [46].

Malignant lesions should be removed through a port in a bag with the lesion extended if needed, while benign lesions can be morcellated [14]. Drains are typically placed at the conclusion of the procedure [22].

### Tips and pitfalls

*General approach -* Case volume is central to governing the integration of LLR into a practice. Beginning with anatomically favourable cases allows for mastery of the technique. Living donor resections in adults can provide an opportunity for paediatric surgeons to perform LLR before transitioning to smaller patients with more friable livers [23]. While a recommendation is to complete 45–75 cases before performing a major hepatectomy, this number depends on prior experience. Additionally, the distinction between major and minor hepatectomy does not necessarily correlate with the difficulty of the procedure [36].

Instead of differentiating between major and minor, tumour location is key to choosing which procedure is suitable to perform via an MIS approach. Starting with a left lateral sectionectomy can lay the framework for success [22, 36]. Surgeons performing LLR should have experience with both hepatobiliary surgery and laparoscopic surgery [24].

While aspects of laparoscopic hepatectomy are similar to open hepatectomy, it is critical to adjust the perspective from which the liver is viewed. There are several recommendations for this. First, while in open liver resection, the view is anteroposterior, in LLR, the liver should be viewed from a caudal to cranial approach. This provides a better perspective of the liver hilum and roots of the hepatic veins and allows for upward dissection and transection of the liver parenchyma [37]. Second, the liver should be viewed as an open door in LLR versus an open book in open surgery [37]. Finally, major veins should be approached from the root rather than anteriorly [37].

### Step-specific

The traditional approach to transection of the parenchyma involves dividing the vasculature structures prior to dividing the lobe, with the resection then made at the zone of ischemia. Alternatively, the vessels can be divided as they are encountered, as is done with a wedge resection. The traditional approach has better vascular control but risks injury to hilar and retrohepatic structures during their dissection [38].

Using the gross stapling technique appears to decrease the risk of bleeding and lead to shorter operative time [22]. If utilizing the CUSA, the dissection can be done on a lower setting than in adults, given the healthy parenchyma of children (50% for aspiration, 3 mL/min for irrigation, 30% for amplitude, cavitation ++ in tissue select mode) [25]. In addition to avoiding damage to healthy parenchyma, a lower setting also causes less change in intrabdominal pressure, which preserves field visualization and less inflow of CO_2_ gas, thereby limiting tissue desiccation [25].

Parenchymal transection can also be completed with individual ligation of Glissonean structures [25]. However, this must be carefully performed given the shorter distance between pedicles as compared to adults [25]. Target vessels should have a distance of at least 7 mm between them to ensure the stump is long enough to avoid stricture [25]. If unable to create enough space between tumour and Glissonean structures or tumour and hepatic vein one technique involves applying a clip to the proximal side and manually clamping the distal side, then using one hand to transect the vessel and ligate the distal side with suture or coagulation [25]. This will prevent the use of force, which can lead to vascular injuries or bleeding from clips slipping off a short stump [25]. Lowering the intrabdominal pressure to 8 mmHg prior to dissecting the parenchyma decreases the risk of air embolism [22].

Indocyanine green (ICG) can be used to help define margins for oncologic resection [37]. It can also be used to evaluate the biliary tract, which is important for centrally located and hilar tumours and for delineation of liver segments [47]. Finally, the use of ICG during complex cases can be used for visualization of flow and to evaluate patency, kinking and stenosis of vessels [48]. A recommended dose is 0.3 mg/kg administered 48 hours before surgery [25].

### Achieving hemostasis and biliostasis

Direct pressure is the best method to control bleeding, which can be done manually if using the hand-assisted technique or with gauze pads [25, 49]. Small bleeders can be addressed with cautery, clips, stapler or hemostatic agents. Larger vessels should be stapled or suture ligated. Severe bleeding can be addressed through increased pneumoperitoneum and decreased airway pressure [25, 36]. If possible, the Pringle maneuver should be avoided as, in the more fragile paediatric liver, it can cause ischemic changes, portal vein thrombosis and damage to the hepatic artery [25]. If necessary, a temporary Pringle can be applied via an external tourniquet positioned through a 5 mm trocar [23]. Low central venous pressure anesthesia may also be used to help control bleeding [29, 38].

The argon cannot be used for biliostasis; this should be addressed with fibrin, cautery, saline-enhanced radiofrequency energy, clip or suture [25].

## MIS for radiofrequency ablation (RFA)

RFA is a management option for patients who cannot be rendered tumour free by resection alone. RFA is most often used in adults for HCC with metastases ≤3 cm as an alternative to resection or as a bridge to transplant [50, 51]. Although rarely used in children, several case series have been published that describe deploying this technique for paediatric patients with both benign and malignant liver lesions, including hepatoblastoma, HCC, adenoma, myofibroblastic tumour, metastases and infiltrative hepatic cysts [51–53]. The majority of patients experienced minimal complications and equivalent long-term survival compared to resection, suggesting that RFA should be considered as an option for the management of paediatric hepatic lesions in select patients [51–53]. RFA can be performed percutaneously, but on occasion may need laparoscopic assistance to allow access to lesions in the dome of the liver, where a percutaneous approach would traverse the diaphragm and potentially injure the lung.

### Indications

Hepatic RFA is indicated as either primary therapy for patients with small neoplasms, to shrink a mass in order to become amenable to resection or as a bridge to transplant [53, 54]. It has been successfully utilized in both the paediatric and adult population for primary and metastatic liver masses [51].

### Contraindications

Absolute contraindications to RFA include intravascular invasion, tumour location within 1 cm of the biliary duct, intrahepatic biliary tree dilation, exophytic location of the tumour or uncorrectable coagulopathy [55]. Although rare in children, end-stage cirrhosis (Child-Pugh C) is also an absolute contraindication [56]. Relative contraindications include extrahepatic metastases, bilioenteric fistula, lesions that are superficial or adjacent to the small intestine or gallbladder and platelets <50,000 /mm^3^ [55]. Additionally, RFA is unable to reliably destroy tumours ≥5 cm, so should not serve as primary therapy for larger tumours [57].

### Surgical approach

Tumour destruction with RFA is achieved by converting electrical current in the radiofrequency range into heat. This heat is passed through a closed-loop circuit consisting of the patient, needle electrodes, a generator and a grounding pad [56]. Success depends on maintaining the correct temperature and accurate targeting of the tumour [56].

RFA can be performed via a percutaneous or laparoscopic approach. A systemic review found that a laparoscopic approach has a higher rate of ablation success and fewer recurrences than a percutaneous approach, but also carries a higher rate of complications [58].

### Tips and pitfalls

Successful RFA is multifactorial, but a key component is accurate assessment of the hepatic tumour prior to the procedure [59]. This allows for optimizing the approach path and for appropriate caution when ablating subcapsular masses, which carry a higher risk of damage to surrounding organs [59]. Tumours that are small or recurrent isoechoic tumours also present a challenge, as they can be difficult to see on ultrasonography. These can be managed through follow-up imaging, use of contrast-enhanced ultrasonography or with transarterial chemoembolization in addition to RFA [59]. Additionally, tumours that are in close proximity to vasculature are at risk of losing heat to the vessels (‘heat-sink’) [57].

## Conclusion

MIS is an evolving technique in the toolbox of paediatric surgeons for the management of hepatic masses. Biopsy performed via MIS carries the standard advantages of a less invasive approach, as well as reducing adhesions and decreasing bleeding complications. Paediatric LLR in appropriate cases is technically feasible with the potential for shorter LOS, decreased blood loss, less pain and improved cosmesis. However, these factors should not take precedence over the need to perform a safe procedure that adheres to oncologic principles. Finally, RFA offers a chance at local disease control for patients who are otherwise not upfront surgical candidates due to tumour characteristics or patient comorbidities.

## Conflicts of interest

We have no conflicts of interest to disclose.

## Funding

We have no funding to declare.

## Figures and Tables

**Figure 1.** Images on the left display the laparoscopic ‘open-door’ view, while images on the right display the ‘open-book’ view utilized in open liver resection [34].

**Table 1.** Case reports describing minimally invasive liver resections for benign and malignant masses in paediatric patients.

Author	Published	Total number	Benign number	Malignant number	Age	Weight	Type of resection (*N*)	Outcomes	Follow-up time	Conversion to open	Complications
Yoon *et al* [22]	2006	1	1	0	5 years	NR	LLS	All alive in good clinical condition	10 months	0	0
Veenstra and Koffron [14]	2016	36	15	21	Med. 2.7 years	NR	Seg.* (10) Sec. (5) HH* (16)	No mortalities, recurrences, or reoperations	Med. 12 months	NR	5
Kwon *et al* [25]	2019	19	5	14	Med. 26 months	Med. 11 kg	PH (12) LLS (2) LL (1) RL (3) RPS (1)	Recurrence (1) Deceased, not from disease (1)	Med. 64 months	1	0
Chen *et al* [26]	2019	1	0	1	3 years	NR	S5 Sec.**	NR	NR	0	0
Murawski *et al* [3]	2021	6	6	0	4 months - 16 years	NR	BS (1) Wedge (5)	NR	NR	1	1
Sandlas *et al* [27]	2021	1	0	1	3 years	NR	RH	NR	NR	0	1
Larghi Laureiro *et al* [23]	2022	10	9	1	Med. 12 years	Med. 49.5 kg	Seg.(4) LLS (4) BS (1) PLS (1)	All alive in good clinical condition	24 months	2	3

(*) Proportion of cases performed via hand-assisted or hybrid laparoscopic approach. (**) Case performed robotically Abbreviations: BS: Bisegmentectomy: HH: Hemihepatectomy; Kg: Kilograms; LL: Left lobectomy; LLS: Left lateral sectionectomy; Med.: Median; NR: Not reported; PH: Partial hepatectomy; RH: Right hepatectomy; RL: Right lobectomy; RPS: Right posterior sectionectomy; Sec: Sectionectomy; Seg: Segmentectomy; PLS: Posterolateral sectionectomy

## References

[1] Campbell MS, Reddy KR (2004). Review article: the evolving role of liver biopsy. Aliment Pharmacol Ther.

[2] Ovchinsky N, Moreira RK, Lefkowitch JH (2012). Liver biopsy in modern clinical practice: a pediatric point-of-view. Adv Anat Pathol.

[3] Murawski M, Weeda VB, Czauderna P (2023). Surgical management in hepatoblastoma: points to take. Pediatr Surg Int.

[4] Weldon CB, Madenci AL, Tiao GM (2020). Evaluation of the diagnostic biopsy approach for children with hepatoblastoma: a report from the children's oncology group AHEP 0731 liver tumor committee. J Pediatr Surg.

[5] Takano Y, Noda J, Yamawaki M (2021). Comparative study of an ultrasound-guided percutaneous biopsy and endoscopic ultrasound-guided fine-needle aspiration for liver tumors. Intern Med.

[6] Shaw C, Shamimi-Noori S (2014). Ultrasound and CT-directed liver biopsy. Clin Liver Dis (Hoboken).

[7] Dezsőfi A, Baumann U, Dhawan A (2015). Liver biopsy in children: position paper of the ESPGHAN Hepatology Committee. J Pediatr Gastroenterol Nutr.

[8] Cohen MB, A-Kader HH, Lambers D (1992). Complications of percutaneous liver biopsy in children. Gastroenterology.

[9] Westheim BH, D8;stensen AB, Aagenæs I (2012). Evaluation of risk factors for bleeding after liver biopsy in children. J Pediatr Gastroenterol Nutr.

[10] Kurakawa KI, Okada A, Bessho K (2022). Major complications after percutaneous biopsy of native or transplanted liver in pediatric patients: a nationwide inpatient database study in Japan. BMC Gastroenterol.

[11] Midia M, Odedra D, Shuster A (2019). Predictors of bleeding complications following percutaneous image-guided liver biopsy: a scoping review. Diagn Interv Radiol.

[12] Kastenberg ZJ, Baertschiger RM, Cuenca AG (2023). Critical elements of pediatric liver cancer surgery. Semin Pediatr Surg.

[13] Kokudo N, Takemura N, Ito K (2020). The history of liver surgery: achievements over the past 50 years. Ann Gastroenterol Surg.

[14] Veenstra MA, Koffron AJ (2016). Minimally-invasive liver resection in pediatric patients: initial experience and outcomes. HPB (Oxford).

[15] Schmelzle M, Krenzien F, Schöning W (2020). Laparoscopic liver resection: indications, limitations, and economic aspects. Langenbecks Arch Surg.

[16] Gigot JF, Glineur D, Santiago Azagra J (2002). Laparoscopic liver resection for malignant liver tumors: preliminary results of a multicenter European study. Ann Surg.

[17] Cortes-Cerisuelo M, Berger M (2019). Minimally-invasive liver resection for liver tumors in children: a snapshot of the current landscape. Mini-Invasive Surg.

[18] Ivanics T, Claasen MP, Patel MS (2022). Long-term outcomes of laparoscopic liver resection for hepatocellular carcinoma: a propensity score matched analysis of a high-volume North American center. Surgery.

[19] Mithany RH, Gerges F, Shahid MH (2023). Operative and hepatic function outcomes of laparoscopic vs. open liver resection: a systematic review and meta-analysis. Cureus.

[20] Tian F, Leng S, Chen J (2023). Long-term outcomes of laparoscopic liver resection versus open liver resection for hepatocellular carcinoma: a single-center 10-year experience. Front Oncol.

[21] European Association for the Study of the Liver (2018). EASL clinical practice guidelines: management of hepatocellular carcinoma. J Hepatol.

[22] Yoon YS, Han HS, Choi YS (2006). Total laparoscopic left lateral sectionectomy performed in a child with benign liver mass. J Pediatr Surg.

[23] Larghi Laureiro Z, Angelico R, Rigamonti A (2022). Minimally invasive hepatopancreatic and biliary surgery in children: a large centre experience and review of the literature. HPB (Oxford).

[24] Murawski M, Gołębiewski A (2021). Laparoscopic resection of liver tumors in children. J Pediatr Surg.

[25] Kwon H, Lee JY, Cho YJ (2019). How to safely perform laparoscopic liver resection for children: a case series of 19 patients. J Pediatr Surg.

[26] Chen DX, Wang SJ, Jiang YN (2019). Robot-assisted gallbladder-preserving hepatectomy for treating S5 hepatoblastoma in a child: a case report and review of the literature. World J Clin Cases.

[27] Sandlas G, Takrouney MH, Jahhav B (2021). Robot-assisted laparoscopic pediatric right hepatectomy. J Pediatr Surg Case Rep.

[28] Viganò L, Tayar C, Laurent A (2009). Laparoscopic liver resection: a systematic review. J Hepatobiliary Pancreat Surg.

[29] Honda M, Uchida K, Irie T (2023). Recent advances in surgical strategies and liver transplantation for hepatoblastoma. Cancer Med.

[30] Pachl M, Lautz TB, Aldrink JH (2024). Minimally invasive and robotic-assisted approaches applied to pediatric surgical oncology. Pediatr Blood Cancer.

[31] Jo SJ, Rhu J, Kim JM (2023). Near-zero open conversion rate of laparoscopic liver resection: a high-volume single-center experience of the past 5 years. Surg Endosc.

[32] Troisi RI, Montalti R, Van Limmen JG (2014). Risk factors and management of conversions to an open approach in laparoscopic liver resection: analysis of 265 consecutive cases. HPB (Oxford).

[33] Cauchy F, Fuks D, Nomi T (2015). Risk factors and consequences of conversion in laparoscopic major liver resection. Br J Surg.

[34] Ai JH, Li JW, Chen J (2013). Feasibility and safety of laparoscopic liver resection for hepatocellular carcinoma with a tumor size of 5–10 cm. PLoS One.

[35] Berardi G, Muttillo EM, Colasanti M (2023). Challenging scenarios and debated indications for laparoscopic liver resections for hepatocellular carcinoma. Cancers (Basel).

[36] Cho JY, Han HS, Wakabayashi G (2018). Practical guidelines for performing laparoscopic liver resection based on the second international laparoscopic liver consensus conference. Surg Oncol.

[37] Wei Chieh AK, Chan A, Rotellar F (2020). Laparoscopic major liver resections: current standards. Int J Surg.

[38] Hunter JG, Spight DH, Sandone C (2018). Atlas of Minimally Invasive Surgical Operations.

[39] Lee S, Park H, Moon SB (2013). Long-term results of biliary atresia in the era of liver transplantation. Pediatr Surg Int.

[40] Vibert E, Kouider A, Gayet B (2004). Laparoscopic anatomic liver resection. HPB (Oxford).

[41] Struecker B, Haber P, D6;llinger R (2018). Comparison of single-port versus standard multiport left lateral liver sectionectomy. Surg Innov.

[42] Liu R, Abu Hilal M, Wakabayashi G (2023). International experts consensus guidelines on robotic liver resection in 2023. World J Gastroenterol.

[43] Zhang K, Zhao D, Xie X (2022). Laparoscopic surgery versus robot-assisted surgery for choledochal cyst excision: a systematic review and meta-analysis. Front Pediatr.

[44] Zhang R, Liu S, Li T (2022). Efficacy of robot-assisted hepaticojejunostomy and laparoscopic-assisted hepaticojejunostomy in pediatric congenital choledochal dilatation: a system review and meta-analysis. Pediatr Surg Int.

[45] (2024). Robot-assisted hepatectomy in pediatrics: two case reports of single enter. Adv Pediatr Surg.

[46] Takasaki K (1998). Glissonean pedicle transection method for hepatic resection: a new concept of liver segmentation. J Hepatobiliary Pancreat Surg.

[47] Rossi G, Tarasconi A, Baiocchi G (2018). Fluorescence guided surgery in liver tumors: applications and advantages. Acta Biomed.

[48] Majlesara A, Golriz M, Hafezi M (2017). Indocyanine green fluorescence imaging in hepatobiliary surgery. Photodiagnosis Photodyn Ther.

[49] Kim HB, Cho HY, Park SH (2015). Laparoscopic ovarian surgery in children and adolescents. JSLS.

[50] Tanaka T, Takata K, Miyayama T (2023). Long-term outcome and eligibility of radiofrequency ablation for hepatocellular carcinoma over 3.0 cm in diameter. Sci Rep.

[51] van Laarhoven S, Baren R, Tamminga RY (2012). Radiofrequency ablation in the treatment of liver tumors in children. J Pediatr Surg.

[52] Hoffer FA, Daw NC, Xiong X (2009). A phase 1/pilot study of radiofrequency ablation for the treatment of recurrent pediatric solid tumors. Cancer.

[53] Hetzer B, Vogel GF, Entenmann A (2020). Stereotactic radiofrequency ablation of a variety of liver masses in children. Int J Hyperthermia.

[54] Kim GA, Shim JH, Kim MJ (2016). Radiofrequency ablation as an alternative to hepatic resection for single small hepatocellular carcinomas. Br J Surg.

[55] Foltz G (2014). Image-guided percutaneous ablation of hepatic malignancies. Semin Intervent Radiol.

[56] Venkatesan AM, Gervais DA, Mueller PR (2006). Percutaneous radiofrequency thermal ablation of primary and metastatic hepatic tumors: current concepts and review of the literature. Semin Intervent Radiol.

[57] Gennaro N, Schiaffino S, Mauri G (2021). The what, the why, and the how of liver ablations: a practical guide for the medical oncologist. Oncology.

[58] Musick JR, Gaskins JT, Martin RCG (2023). A meta-analysis and systematic review of the comparison of laparoscopic ablation to percutaneous ablation for hepatic malignancies. Int J Clin Oncol.

[59] Kim SK, Rhim H, Kim YS (2005). Radiofrequency thermal ablation of hepatic tumors: pitfalls and challenges. Abdom Imaging.

# Other: Role of minimally invasive surgery in paediatric pulmonary metastatic disease

**DOI:** 10.3332/ecancer.2025.2026

**Published:** 2025-10-23

**Authors:** Timothy B Lautz, Rodrigo Chaves Ribeiro

**Affiliations:** 1 Division of Paediatric Surgery, Ann & Robert H Lurie Children’s Hospital of Chicago, Chicago, IL 60611, USA; 2 Department of Paediatric Surgery, Children’s Cancer Hospital, Barretos Cancer Hospital, Barretos 14784058, Brazil

**Keywords:** children, thoracoscopy, thoracotomy, pulmonary metastasectomy

## Abstract

The role of minimally invasive surgery (MIS) in paediatric pulmonary metastasectomy is evolving, reflecting advances in imaging, localisation and instrumentation. Compared with thoracotomy, thoracoscopy offers benefits of reduced postoperative pain, shorter recovery and easier reoperation. However, limitations include anaesthetic challenges in smaller children and lack of manual palpation, which may miss subpleural nodules, which is particularly important in chemoresistant tumours such as osteosarcoma and nonrhabdomyosarcoma soft tissue sarcoma. MIS is most suitable for limited disease, peripheral nodules and histologies where complete manual exploration is unnecessary. Indications depend on tumour type, number and location of lesions, as well as patient stability and institutional expertise. Advances in nodule localization—such as wire or coil marking, fluorescence imaging and radiotracers—have improved thoracoscopic precision. Wedge resection remains preferred for peripheral nodules, with anatomic resection reserved for central or larger lesions. MIS contraindications include extensive disease, inability to tolerate single-lung ventilation or lack of required resources. Optimal outcomes depend on experienced multidisciplinary teams and readiness to convert to open surgery when needed. Overall, thoracoscopy is a safe, effective option in selected paediatric patients, providing therapeutic benefit while minimising morbidity when applied judiciously to tumour biology and disease extent.

## Introduction

The role of a minimally invasive approach to lung metastases in children is in evolution. Minimally invasive surgery (MIS) can offer significant benefits, including reduced postoperative pain and quicker recovery when compared to open surgery with thoracotomy. In addition, reoperation after thoracoscopy is significantly less complicated than after thoracotomy. After thoracotomy, there may be a fusion of the intercostal space, which complicates repeat access, often increases adhesions, and can contribute to future scoliosis. However, thoracoscopy also has its limitations, particularly in smaller children, including anaesthetic challenges, equipment requirements, and importantly, an inability to palpate the lung parenchyma. In current practice, thoracoscopy is the preferred approach when there is limited disease burden, all nodules are peripheral or a lobectomy is planned in patients with tumour types that do not require comprehensive palpation of all lung surfaces. Additional applications remain under investigation.

## MIS indications

Pulmonary metastasectomy (PM) is generally indicated for patients who have had successful local control and have residual pulmonary metastases after induction chemotherapy. Pulmonary metastatic response to chemotherapy is assessed with computed tomography (CT). For tumours that are generally responsive to chemotherapy and/or radiotherapy (RT), including Wilms tumour, Ewing sarcoma, germ cell tumour, rhabdomyosarcoma and hepatoblastoma, the role of PM is fairly limited. For these tumours, metastases will often resolve with initial chemotherapy. Whole lung RT is the first-line therapy for residual pulmonary disease in Wilms tumour, Ewing sarcoma, rhabdomyosarcoma and other radiosensitive tumours. However, for tumours that are poorly responsive to chemotherapy and RT, including osteosarcoma, nonrhabdomyosarcoma soft tissue sarcomas (NRSTS) and adrenocortical tumour, PM plays a much more critical therapeutic role.

For patients who will benefit from PM, the use of a MIS approach (i.e., thoracoscopy) is influenced by the tumour type, available resources, as well as the size, number and location of the nodules. Thoracoscopy is also dependent on the patient’s hemodynamic stability and ability to tolerate one-lung ventilation.

- Tumour type: For some tumours, including osteosarcoma and NRSTS, which are relatively resistant to chemotherapy and radiation, aggressive attempts to remove even tiny nodules below the detection sensitivity of CT may be important. Multiple studies have shown that thoracotomy with palpation of the lung can identify lesions not detected on pre-operative CT in 20%–25% of patients with osteosarcoma. Without manual palpation, the detection of these occult nodules is not possible with thoracoscopy. Despite this evidence that open surgery often allows for the detection and removal of viable nodules not detected on preoperative imaging, this has never been shown to produce a survival benefit. Therefore, while thoracoscopy is used less frequently for osteosarcoma and NRSTS compared to other tumour types, the MIS surgical approach is perfectly acceptable in select clinical scenarios. In fact, patients with osteosarcoma and oligometastatic pulmonary metastatic disease are currently eligible for a phase III randomised trial in the Children’s Oncology Group comparing outcomes with thoracoscopy versus thoracotomy (AOST2031).
- Nodule number: In general, thoracoscopy is reserved for patients with no more than 3–4 lesions per side. This is driven both by limitations in the ability to detect and remove more than a few nodules, as well as considerations related to the impact on pulmonary function. MIS stapled wedge resections generally require the removal of a larger cuff of normal lung tissue compared to open wedge resections. While well tolerated for a few resections, a larger number of MIS wedges can have negative implications on pulmonary reserve due to the removal of excessive normal lung.
- Nodule location and size: MIS approach for wedge resection, lobectomy and segmentectomy are all safe and feasible. Wedge resection is generally reserved for peripheral nodules, <3 cm in size. Larger and/or central nodules (located <2 cm from the bronchus or vessels) are best treated with segmentectomies or lobectomies, which can still be performed by MIS, depending on the team's experience. When tumour involves multiple lobes of the lung and includes one or more central nodules, anatomic lobectomy or segmentectomy may not be feasible. In these cases, thoracotomy may allow for an enucleation procedure that is not possible with thoracoscopy.

## MIS contraindications

Contraindications for MIS PM include both patient factors and available resources.

- Patient factors: MIS is contraindicated for patients presenting with more extensive pulmonary disease, hemodynamic instability or pulmonary insufficiency, limiting the ability to tolerate one lung ventilation. While long-term survival in children with extensive pulmonary disease is rare, surgery may prolong survival and produce some long-term cures. However, an open approach to surgery in these more extreme scenarios is recommended.
- Available resources: Successful MIS PM relies on the availability of appropriate resources, as well as the experience of the surgeon, anaesthesiologist and other team members. While larger nodules located very close to the pleural surface can often be identified by standard white light imaging and palpation, smaller and (even slightly) deeper nodules, often benefit from localisation. Adequate localisation can reduce the potential need for conversion to open surgery to facilitate nodule identification. A variety of localisation techniques, ranging from wire or coil placement under CT-guidance, fluorescence-guided surgery or radiotracers are possible, and the choice of technique is based on surgeon and institutional preference. Nonetheless, the availability of one or more reliable localisation techniques is essential.

Other resource considerations include the availability of standard MIS equipment, near-infrared imaging equipment if fluorescence-guided surgery is employed for nodule localisation, and intra-operative fluoroscopy if microcoils or fiducials are used for localisation.

Finally, experience with and equipment for one-lung ventilation are essential.

## Surgical approach

### Patient position

The choice of surgical approach depends on lesion characteristics: site, size and involvement of one or both lungs. The patient's position must allow for the greatest access to the areas of interest and uses gravity to aid in keeping the uninvolved lung or other tissue out of the field of view. The procedure can be done in the lateral position and in specific cases in the supine or prone position. The lateral position provides an excellent visualisation and access to all surfaces of the lung. For lateral position, an axillary roll may assist in positioning. The surgical team stands in front of the patient for an anterior approach. The monitor is behind the patient, but it is advantageous to have two monitors, one on either side of the table.

While some surgeons utilise supine positioning for bilateral thoracoscopy, exposure is optimised by placing the patient in a full decubitus position, even though that requires flipping the patient between the two sides for bilateral surgery. A break in the bed helps open the intercostal spaces, while an axillary roll helps avoid nerve pressure injury.

### Trocar sites

Trocar sites are triangulated based on the expected location of the pulmonary nodules. In general, for thoracoscopy, the surgeon works from the front (anterior side) of the patient. The 5th intercostal space, anterior axillary line, typically aligns with the major fissure and is often an optimal location for the camera port. However, the camera port can be inserted anywhere along the anterior axillary line from the 5–9th inter-costal space. Care must be taken to avoid diaphragm and sub-diaphragmatic injury when entering the chest below the 6th intercostal space. The principle of triangulating the lesion with the camera port at the apex applies.

For a robotic approach, trocar placement for an anterior approach can be similarly employed. However, for robotic surgery, placement of ports in a row in the 8–9th intercostal space (just above the diaphragm) can be effective for all types of nodule locations. An additional 4th arm can be placed 2–3 rib spaces higher, in the anterior axillary line when needed.

### Surgical technique

MIS PM procedures are performed under general endotracheal anaesthesia. Single lung ventilation may be helpful, but is not mandatory since thoracic insufflation provides adequate lung collapse for surgery. Double-lumen endotracheal tubes work well in patients over 30 kg. For smaller children, single lung ventilation is more challenging, but options include bronchial blockers or selective intubation of the contralateral bronchus.

Options for wedge resection with MIS PM include bipolar energy devices (e.g., Ligasure) or surgical stapling devices. Bipolar energy devices work best for smaller superficial lesions. They may allow for a smaller cuff of normal surrounding lung tissue, but they also have an increased risk of air leak. The pleural defect can be oversewn to reduce this risk. Surgical staples are preferred for larger and deeper lesions but can also be used even for small peripheral nodules.

Steps of the procedure for MIS pulmonary wedge resection after port placement include [1] takedown of the inferior pulmonary ligament and/or opening of the anterior and/or posterior pleura as needed for mobilisation [2], inspection to identify the known lesion(s) and to assess for an occult disease in the lung or pleural space [3], application of adjunctive measures including fluorescence visualisation and/or fluoroscopy for localisation coils [4], wedge resection using bipolar energy or stapling device as above [5], inspection, possible chest tube placement and closure. Chest tubes are used selectively after isolated pulmonary wedge resection and can often be avoided for straightforward solitary wedges.

Adjunctive localisation techniques are recommended for all nodules deeper than a few mm below the pleural surface [6]. Options including hookwires, dyes, microcoils, fiducial markers, contrast media and radiotracers are well described in the literature [7, 8]. Fluorescence-guided surgery with systemically administered fluorophores, including indocyanine green [9–12] and newer agents such as pafolacianine (Cytalux), is also gaining in popularity, but is restricted in utility to nodules within about 5–8 mm of the pleural surface due to the inherent limitations in the depth of penetration of near-infrared light.

When feasible, a wedge resection is preferred over larger anatomic resections, such as lobectomy, to maintain maximum pulmonary reserve, considering there is a significant chance of additional recurrence. When anatomic resection is required, collaboration with an experienced thoracic surgeon can often permit segmentectomy rather than lobectomy. Adequate lymphadenectomy is an essential component of anatomic resections. Robotic-assisted surgery can be particularly helpful for anatomic resections.

### Tips and pitfall

- Work with an experienced staff and anaesthesiologist
- Ensure availability of appropriate materials and equipment, including but not limited to resources for one-lung ventilation, nodule localisation, MIS equipment (including fluorescence-detection if desired), appropriate energy devices and staplers and so on.
- Work with a multidisciplinary team to optimise decision-making around indications, timing and approach for PM.
- Do not be afraid to convert the surgery to open when the MIS route is difficult.
- Try to preserve as much lung parenchyma as possible while ensuring negative margins.

## Conclusion

MIS is a good option in specific paediatric pulmonary metastatic disease cases. The number and position of the lesions and the histologic type of tumour must be considered. The material and experience of the surgical team are essential factors in the decision.

## Conflicts of interest

The authors declare no conflicts of interest regarding this manuscript.

## Funding

There is no specific funding for this work.

## References

[1] Liu X, Wu Z, Li X (2024). Thoracoscopic versus thoracotomy lobectomy in children with congenital lung lesions: a systematic review and meta-analysis. ANZ J Surg.

[2] Kuo C, Malvar J, Chi YY (2023). Survival outcomes and surgical morbidity based on surgical approach to pulmonary metastasectomy in pediatric, adolescent and young adult patients with osteosarcoma. Cancer Med.

[3] Lautz TB, Farooqui Z, Jenkins T (2021). Thoracoscopy vs thoracotomy for the management of metastatic osteosarcoma: a pediatric surgical oncology research collaborative study. Int J Cancer.

[4] Yamataka A, Koga H, Ochi T (2017). Pulmonary lobectomy techniques in infants and children. Pediatr Surg Int.

[5] Bonnard A (2020). Thoracoscopic lobectomy for congenital pulmonary airway malformation: where are we in 2019?. Eur J Pediatr Surg.

[6] Lai SW, Rothenberg SS (2019). Culture of safety and error traps in pediatric thoracoscopy. Semin Pediatr Surg.

[7] Rothenberg SS (2007). Thoracoscopic pulmonary surgery. Semin Pediatr Surg.

[8] Lin MW, Chen JS (2016). Image-guided techniques for localizing pulmonary nodules in thoracoscopic surgery. J Thorac Dis.

[9] Lake CM, Bondoc AJ, Dasgupta R (2021). Indocyanine green is a sensitive adjunct in the identification and surgical management of local and metastatic hepatoblastoma. Cancer Med.

[10] Yoshida M, Tanaka M, Kitagawa N (2022). Clinicopathological study of surgery for pulmonary metastases of hepatoblastoma with indocyanine green fluorescent imaging. Pediatr Blood Cancer.

[11] Delgado-Miguel C, Estefanía K, San Basilio M (2023). Indocyanine green navigation in minimally invasive resection of multiple metachronous pulmonary metastases of hepatoblastoma. Thorac Cancer.

[12] Abdelhafeez AH, Mothi SS, Pio L (2023). Feasibility of indocyanine green-guided localization of pulmonary nodules in children with solid tumors. Pediatr Blood Cancer.

# Other: Ovarian neoplasms — the role of minimally invasive surgery

**DOI:** 10.3332/ecancer.2025.2030

**Published:** 2025-10-23

**Authors:** Alyssa Stetson, Roshni Dasgupta

**Affiliations:** Division of Paediatric General and Thoracic Surgery, Cincinnati Children Hospital Medical Center, University of Cincinnati, Cincinnati OH 45229, USA

**Keywords:** ovarian neoplasm, paediatric surgery, paediatric gynecology, minimally invasive surgery

## Abstract

The role of minimally invasive surgery (MIS) for ovarian neoplasms in paediatric patients depends on multiple factors. First, it is important to consider the risk of malignancy, which can be difficult to assess, especially in the setting of torsion. Second, when possible ovarian sparing surgery (OSS) should be performed. In certain settings MIS to perform OSS may carry a higher risk of tumour or cyst spillage. MIS can also play a role in diagnosis, via staging or biopsy. When performed, MIS can offer improved visualization of the contralateral ovary and other abdominal structures. Overall, MIS for ovarian neoplasms offers improved visualization of pelvic structures and decreased risk of adhesions in addition to the traditional benefits of MIS. However, these advantages should not supersede the need to achieve complete oncologic resection and to minimize the risk of capsule rupture.

## Introduction

Ovarian neoplasms in paediatric patients present a unique challenge due to the need to balance ovarian preservation with complete resection of malignancy. Patients with benign ovarian masses should undergo ovarian sparing surgery (OSS), but failure to perform an oncologic resection of a malignant tumour can result in the need for additional surgery and adjuvant therapy, and an increased chance of recurrence [1, 2]. Adding to the challenge, it can be difficult to standardise care as a paediatric patient with an ovarian mass may be cared for by a variety of specialists (i.e., paediatric surgeons, paediatric and adolescent gynecologists and adult gynecologists) [3]. Finally, children with ovarian malignancies may present urgently or emergently with torsion, which can limit the extent of workup prior to surgery [4].

Choosing to take an open or minimally invasive surgery (MIS) approach depends on the likelihood of malignancy, yet unfortunately, this can be difficult to characterise. The vast majority (75%–97%) of ovarian masses in children are benign [5–9]. Various studies have sought to discover a method to identify those that are malignant. While malignant tumours are usually larger, the cutoff at which a mass is almost certainly malignant spans a wide range from 5 to 10 cm, and additionally, some studies have found no difference in size between benign and malignant masses [2, 10–12]. Malignancies are more likely to have large (≥ 2–3 mm) septations and nonhyperechoic solid nodular or papillary components, but these features are also not fully predictive, with up to 16% of cystic-appearing masses ultimately malignant [5, 10]. A panel of tumour markers is the most sensitive and specific manner to identify malignancy, but even still, tumour markers may be elevated in 20% of benign masses, especially in the setting of torsion. Additionally, if a patient presents with torsion, it may not be possible to wait for these laboratory tests to result [2, 11]. If time permits, pathology/molecular biology may be used to assist in diagnosis and guide decision-making strategies [13]. The technical approach also depends on the surgeon comfort, with some areas of the world having limited access to resources and training that are necessary to safely perform laparoscopic surgery [14]. This can be further compounded by delays in presentation secondary to long transportation distances and/or long wait times to see a surgeon [14].

OSS is recommended for benign ovarian masses due to its numerous benefits compared to oophorectomy: it decreases the risk of early menopause secondary to premature ovarian failure, offers improved sexual health and higher success with ovarian stimulation for assisted reproduction and avoids low bone density, neurologic disease and cardiac disease in adulthood [5, 15]. Laparoscopic OSS has low rates of conversion to open, and when compared to open surgery, the operative time, estimated blood loss, haemoglobin drop postoperatively, length of stay and complication rates are equivalent or superior [5, 7, 16, 17]. Furthermore, in addition to the other well-documented benefits of MIS surgery (improved cosmesis, decreased pain, shorter length of stay and reduced perioperative morbidity), MIS causes fewer pelvic adhesions which can contribute to improved fertility [16, 18].

## Indications for MIS

### Benign disease

Procedures for benign disease include cyst aspiration, fulguration and OSS. A laparoscopic approach is well established for cyst aspiration and fulguration [16]. More attention has been paid to the outcomes of laparoscopic OSS.

One main concern with laparoscopic OSS is tumour spillage, which can carry the risk for chemical peritonitis for dermoid tumours. While some studies have identified a higher risk of tumour spillage with MIS approach, others have found equivalent rates of spillage regardless of whether the surgery was performed via open or laparoscopy, with increased rates of spillage based only on whether the procedure was OSS versus oophorectomy [7]. Additionally, given the rarity of chemical peritonitis secondary to dermoid spillage, a laparoscopic approach remains reasonable [17, 19]. Conversion rates, which are approximately 5%–10%, do not indicate a high likelihood of failure to complete the procedure via an MIS approach for technical reasons [7, 20].

Another concern with a laparoscopic OSS approach is the risk of cyst or tumour recurrence. However, multiple studies have found either equivalent rates of cystic rupture between open and laparoscopic approaches or equivalent rates of recurrence, even if there is a higher rate of rupture with laparoscopic surgery [21, 22]. Likewise, there does not appear to be higher rates of solid tumour recurrence with MIS [19, 20]**.**

Finally, MIS does not affect ovarian viability, with equivalent blood flow and follicle presence seen after MIS as with open surgery [17, 19].

### Malignant disease

There are several indications for MIS for malignant ovarian neoplasms. Diagnostic laparoscopy can be used to identify the precise location and nature of the neoplasm, including taking a biopsy if indicated [1]. Diagnostic laparoscopy also provides an opportunity for staging, with superior visualisation of the sus-mesocolic and anterior parietal peritoneum as compared to open surgery, as well as easy inspection of the contralateral ovary and diaphragm [1, 23]. In adults, laparoscopy has also been used for debulking advanced cancer and assessing response to neoadjuvant chemotherapy [18, 24, 25]. However, literature is limited in the paediatric population and so there is currently no data on whether a laparoscopic approach should be taken for this indication in children.

### Emergency/unknown malignancy status

Ovarian torsion is uncommon in paediatric patients, occurring at a rate of approximately 4.9 per 100,000. Over half of patients have an associated adnexal mass, most commonly a benign cyst or teratoma [26]. Given that ovarian viability is at stake, there can be limited time for diagnostic work up. Laparoscopy is a reasonable approach for most cases of ovarian torsion [26]. However, in a mass that is at least partially solid, oncologic principles should be maintained, which may require open surgery [4].

## MIS contraindications

Laparoscopic surgery has become the standard of care for operating on benign ovarian masses [27]. However, a laparoscopic approach for dermoid cysts remains controversial due to the possibility of chemical peritonitis and upstaging with spillage [21, 28]. The risk of spillage appears to be more significant with the laparoscopic approach, bilateral cysts and larger masses. While the true risk of chemical peritonitis following cyst spillage remains unknown, some authors recommend an open approach for bilateral dermoid cysts and those larger than 5–8 cm [17, 21]. However, so long as the mass is mobile, others recommend continuing with laparoscopic surgery even for larger masses due to the improved visualisation [4].

Laparoscopic surgery is contraindicated for masses that are known to be malignant or have features highly suspicious for malignancy (≥8 cm, thick walls, loculations or solid components) [10]. This is for several reasons. One, proper staging requires palpation in addition to visual inspection of both ovaries, the omentum and peritoneum [10, 29]. Second, ensuring complete resection is essential to minimising risk for recurrence [30]. Finally, tumour rupture is a feared complication of ovarian surgery as it results in tumour seeding of the abdomen and upstaging of the cancer [31]. There is concern that laparoscopic surgery is associated with increased risk of rupture due to the fact that, unlike in open surgery, MIS requires manipulation of the ovary during the procedure and at the time of extraction [31, 32]. Patients with a ruptured capsule may require adjuvant chemotherapy and experience an estimated increased risk of rate of progression rate of 1.92% and increased rate of all-cause mortality by 1.4%–1.5% [31, 32]. However, literature in paediatric patients has not consistently shown the same association between an MIS approach and tumour rupture, nor inferior outcomes with tumour rupture [22]. Given this ongoing debate, the decision regarding approach should be made on a case-by-case decision with all patient factors considered.

## Surgical approach

### Patient position

Patients should be placed in the supine position or in the lithotomy position if access to the vagina will be required [33, 34]. Steep Trendelenburg is helpful for visualisation, but the patient can be tilted with the head and shoulders left supine if the patient cannot tolerate elevated intracranial pressure [33].

### Trocar sites

Entry via Palmer’s point is recommended if there are significant adhesions, abnormal anatomy or concern about rupture during trocar placement due to the size of the mass [35]. In general, three trocar sites are recommended: a 10–12 mm port through the inferior umbilical fold, a 5 mm suprapubic port located 2 cm above the pubic symphysis and the third port at the counter-McBurney point [16]. Additional ports can be placed in the upper abdomen if extensive debulking is required [18]. The lateral trocars should be inserted under direct visualisation to avoid damage to the inferior epigastric arteries [34].

Single incision laparoscopic surgery (SILS) is an alternative to conventional laparoscopy [36]. As suturing is rarely required, ovarian surgery can be highly suitable for SILS [4]. For cyst removal, SILS has been shown to be safe and effective in adults [37, 38]. Literature in children is limited, but SILS has been successfully used for paediatric ovarian cryopreservation [39].

### Surgical technique

The view of the surgical field differs in children due to the higher upper margin of the bladder, smaller uterus fundus, relatively elongated cervix and smaller ovarian volume [16]. The bowel, other than the sigmoid, should fall away once the patient is placed in Trendelenburg. If additional visualisation is needed, an atraumatic grasper can be used to flip away loops of small bowel [40].

For masses that are thought to be benign, it is still essential to inspect the contralateral ovary and abdominal cavity, including the pouch of Douglas, pelvic and abdominal parietal peritoneum, paracolic gutters, diaphragm, mesentery and small and large bowel [2, 18]. Biopsies and washings should be taken if there are any suspicious findings; however, if the contralateral ovary appears normal on imaging and intraoperatively, it should not be biopsied [18]. Any spillage should be lavaged and documented [41].

The surgical procedure depends on the nature of the mass. Cysts should be aspirated, then the cyst wall resected and removed via an Endo Catch bag [42]. For benign solid neoplasms, OSS is performed by incising the cortex, separating the tumour from the capsule using either blunt or sharp dissection while ensuring the capsule remains intact and removing the tumour [42, 43]. If there is an intraoperative concern for malignancy, a frozen biopsy should be sent in order to inform the decision as to the correct procedure [42]. For malignancies, oophorectomy is performed by incising the peritoneum between the infundibulopelvic (IP) ligament and ureter. Following this, the retroperitoneal space should be dissected to separate the IP ligament from the ureter. The IP ligament, mesovarium and utero-ovarium ligament can then be ligated, and the ovary removed [35, 40].

A surgical pouch should be used to remove the mass or the ovary [16]. If the mass is cystic, it should be placed in the pouch before it is punctured and aspirated [44]. Solid masses may require an additional trocar site or extending an incision. The opening of the bag should be externalised through the incision and the mass manually morcellated before removal [34].

Robotic surgery is increasingly used in adult patients and has been shown to have similar rates of complications and similar oncologic outcomes when compared to open or laparoscopic procedures[45, 18]. The robotic approach for ovarian tumours is less commonly employed in children, and therefore, data are limited [46–48]. One case series described 11 patients, with diagnoses including 6 dermoid tumours, 2 serous papillary cystadenofibromas of the fallopian tube and 3 cystadenomas. An 8 mm optic port and an additional 2–3 accessory ports were used. There were no recurrences or complications with a median follow up of 2.1 years [49]. Alternatively, given the potential for interference between the robotic arms in smaller paediatric abdominal cavities, a single port can be used [50]. One case series describes three patients who underwent robotic OSS for mature teratomas via a 2.5 cm single port site. There was no tumour recurrence during a follow up of 6–18 months [51].

## Tips and pitfalls

There are several key aspects of ovarian surgery. The first priority is ensuring the best chance at successful oncologic outcomes. Therefore, the contralateral ovary needs to be inspected for disease. This is especially true of ovarian teratomas, where contralateral disease occurs in 5%–10% of patients [1, 30, 44]. Additionally, great care must be taken to avoid tumour spillage. For cystic masses, perforation can occur during trocar placement [8]. If intraoperative spillage does occur, copious lavage in reverse Trendelenburg should be performed [8].

Oncologic outcomes need to be balanced against preserving fertility. For patients who present with torsion, the ovary should be given a chance to recover. Even an ovary that appears necrosed intraoperatively can demonstrate blood flow and follicles on future ultrasound [19]. Avoiding biopsy of the contralateral ovary without a clear indication can also help preserve fertility [2].

Additional fertility-preserving techniques should be considered at the time of surgery. For prepubertal children, ovarian stimulation techniques are ineffective, so ovarian tissue cryopreservation (OTC) is the only option [52, 53]. OTC involves the removal of a section of ovarian cortex, freezing and subsequent reimplantation [52]. Data are limited in paediatric patients, with a systematic review of 1,019 children who underwent OTC reporting 18 patients who underwent auto-transplantation and 10 live births [54]. Post-menarche adolescents can also undergo mature oocyte freezing [53].

Finally, there are many critical structures in the pelvis that need to be preserved. Mobilisation of the sigmoid colon can improve visualisation of the left ovary [34]. Ureteral injury occurs in 1%–2% of gynecologic surgeries in adults, so careful attention must be paid to these structures [34].

## Conclusion

Taking a minimally invasive approach for paediatric patients who present with ovarian neoplasms offers the traditional benefits of MIS, as well as fewer adhesions and improved visualisation of pelvic structures, including the contralateral ovary. However, these advantages must be weighed against the need to achieve complete oncologic resection and to minimise the risk of capsule rupture. Additionally, surgeon comfort level may vary with surgeons in some areas constrained by limited access to technical training in MIS. The decision regarding surgical approach should therefore be made on an individualised basis, accounting for all patient factors.

## Conflicts of interest

The authors have no conflicts of interest or funding to declare.

## Funding

There is no funding to disclose.

## References

[1] Sarnacki S, Brisse H (2011). Surgery of ovarian tumors in children. Horm Res Paediatr.

[2] Renaud EJ (2019). Ovarian masses in the child and adolescent: an American Pediatric Surgical Association outcomes and evidence-based practice committee systematic review. J Pediatr Surg.

[3] Trotman GE (2017). Rate of oophorectomy for benign indications in a Children's Hospital: influence of a gynecologist. J Pediatr Adolesc Gynecol.

[4] Fuchs J (2015). The role of minimally invasive surgery in pediatric solid tumors. Pediatr Surg Int.

[5] Seckin B (2011). Laparoscopic treatment of ovarian cysts in adolescents and young adults. J Pediatr Adolesc Gynecol.

[6] Grabowski A, Korlacki W, Pasierbek M (2014). Laparoscopy in elective and emergency management of ovarian pathology in children and adolescents. Wideochir Inne Tech Maloinwazyjne.

[7] Knaus ME (2022). Laparoscopy versus laparotomy for pediatric ovarian dermoids. J Pediatr Surg.

[8] Mosbah A, Nabiel Y, Megahed N (2016). Laparoscopic treatment of ovarian tumors in children: an experience of seven cases in Mansoura University Hospital. Gynecol Surg.

[9] Morowitz M, Huff D, Allmen D (2003). Epithelial ovarian tumors in children: a retrospective analysis. J Pediatr Surg.

[10] Phelps HM, Lovvorn HN (2018). Minimally invasive surgery in pediatric surgical oncology. Children (Basel).

[11] Spinelli C (2012). The role of tumor markers in the surgical approach of ovarian masses in pediatric age: a 10-year study and a literature review. Ann Surg Oncol.

[12] Al Jama FE (2011). Ovarian tumors in children and adolescents--a clinical study of 52 patients in a university hospital. J Pediatr Adolesc Gynecol.

[13] Ray-Coquard I (2018). Non-epithelial ovarian cancer: ESMO clinical practice guidelines for diagnosis, treatment and follow-up. Ann Oncol.

[14] Uzun M (2025). Challenges and innovations in minimally invasive surgery for pediatric patients in Africa: a comprehensive review. Health Sci Rep.

[15] Lawrence AE, Minneci PC, Deans KJ (2019). Ovary-sparing surgery for benign pediatric ovarian masses. Curr Opin Pediatr.

[16] Kim HB (2015). Laparoscopic ovarian surgery in children and adolescents. JSLS.

[17] Piotrowska-Gall A (2024). Laparoscopic ovarian-sparing surgery for the management of benign ovarian lesions in pediatric patients: a retrospective analysis. J Pediatr Surg.

[18] Nezhat FR (2013). Role of minimally invasive surgery in ovarian cancer. J Minim Invasive Gynecol.

[19] Light-Olson H (2023). Minimally invasive adnexa-sparing surgery for benign ovarian and paratubal masses in children. J Pediatr Surg.

[20] Abbas PI (2016). Ovarian-sparing surgery in pediatric benign ovarian tumors. J Pediatr Adolesc Gynecol.

[21] Childress KJ (2017). Intraoperative rupture of ovarian dermoid cysts in the pediatric and adolescent population: should this change your surgical management?. J Pediatr Adolesc Gynecol.

[22] Yousef Y, Pucci V, Emil S (2016). The relationship between intraoperative rupture and recurrence of pediatric ovarian neoplasms: preliminary observations. J Pediatr Adolesc Gynecol.

[23] Galazka P (2019). Minimally invasive surgery in pediatric oncology: proposal of guidelines. Anticancer Res.

[24] Corrado G (2015). Laparoscopic debulking surgery in the management of advanced ovarian cancer after neoadjuvant chemotherapy. Int J Gynecol Cancer.

[25] Tozzi R (2024). Feasibility of laparoscopic Visceral-Peritoneal Debulking (L-VPD) in patients with stage III-IV ovarian cancer: the ULTRA-LAP trial pilot study. J Gynecol Oncol.

[26] Nguyen KP (2022). Ovarian torsion: presentation and management in a pediatric patient. Case Rep Obstet Gynecol.

[27] Ergun-Longmire B, Greydanus DE (2024). Ovarian tumors in the pediatric population: an update. Dis Mon.

[28] von Allmen D (2005). Malignant lesions of the ovary in childhood. Semin Pediatr Surg.

[29] Oltmann SC (2010). Pediatric ovarian malignancies: how efficacious are current staging practices?. J Pediatr Surg.

[30] Terenziani M (2015). Mature and immature teratoma: a report from the second Italian pediatric study. Pediatr Blood Cancer.

[31] Dioun S (2021). Intraoperative rupture of the ovarian capsule in early-stage ovarian cancer: a meta-analysis. Obstet Gynecol.

[32] Matsuo K (2020). Minimally invasive surgery and risk of capsule rupture for women with early-stage ovarian cancer. JAMA Oncol.

[33] Xiao JS (2021). Laparoscopic gynaecological surgery in the context of maintaining normal intracranial pressure. BMJ Case Rep.

[34] Lozada Y, Bhagavath B (2017). A review of laparoscopic salpingo-oophorectomy: technique and perioperative considerations. J Minim Invasive Gynecol.

[35] Lawson AA (2022). Oophorectomy.

[36] Misawa A (2023). Successful use of single-port laparoscopic surgery for ovarian cyst removal during pregnancy: a case series of three cases. J Surg Case Rep.

[37] Wang X, Li Y (2021). Comparison of perioperative outcomes of single-port laparoscopy, three-port laparoscopy and conventional laparotomy in removing giant ovarian cysts larger than 15 cm. BMC Surg.

[38] Schmitt A (2024). The effects of a laparoscopy by single-port endoscopic access in benign adnexal surgery: a randomized controlled trial. J Minim Invasive Gynecol.

[39] Metcalfe K (2022). Conventional 3-port vs. single-incision laparoscopic oophorectomy for ovarian cryopreservation in paediatric surgery: a retrospective case-note review. Ann Pediatr Surg.

[40] Ellison E (2022). Zollinger’s Atlas of Surgical Operations.

[41] Braungart S (2022). Standardizing the surgical management of benign ovarian tumors in children and adolescents: a best practice Delphi consensus statement. Pediatr Blood Cancer.

[42] Raźnikiewicz A, Korlacki W, Grabowski A (2020). The role of laparoscopy in paediatric and adolescent gynaecology. Wideochir Inne Tech Maloinwazyjne.

[43] Khai T (2023). Characteristics and outcomes of pediatric ovarian germ cell tumors: a report of 162 cases. Biomed Res Ther.

[44] Tatekawa Y (2021). Surgical approach to three pediatric patients with ovarian tumors: laparoscopic excision using a specimen retrieval bag. J Surg Case Rep.

[45] Park J (2023). Robotic surgery in gynecology: the present and the future. Obstet Gynecol Sci.

[46] Gallotta V (2023). Robotic surgery in ovarian cancer. Best Pract Res Clin Obstet Gynaecol.

[47] Pelizzo G, Nakib G, Calcaterra V (2019). Pediatric and adolescent gynecology: treatment perspectives in minimally invasive surgery. Pediatr Rep.

[48] Esposito C (2024). Robotic-assisted surgery for gynecological indications in children and adolescents: European multicenter report. J Robot Surg.

[49] Vatta F (2021). Robotics-assisted pediatric oncology surgery-a preliminary single-center report and a systematic review of published studies. Front Pediatr.

[50] Xie XX (2019). Robotic-assisted resection of ovarian tumors in children: a case report and review of literature. World J Clin Cases.

[51] Xu D (2022). Ensuring safety and feasibility for resection of pediatric benign ovarian tumors by single-port robot-assisted laparoscopic surgery using the da Vinci Xi system. Front Surg.

[52] Dinikina Y (2021). Ovarian tissue cryopreservation in prepubertal patients with oncological diseases: multidisciplinary approach and outcomes. J Matern Fetal Neonatal Med.

[53] Dolmans MM (2021). Fertility preservation: how to preserve ovarian function in children, adolescents and adults. J Clin Med.

[54] Corkum KS (2019). Fertility and hormone preservation and restoration for female children and adolescents receiving gonadotoxic cancer treatments: a systematic review. J Pediatr Surg.

# Other: The role of minimally invasive surgery in paediatric mediastinal masses and thoracic tumours

**DOI:** 10.3332/ecancer.2025.2027

**Published:** 2025-10-23

**Authors:** Jaime Shalkow-Klincovstein, Cristian Puerta, Andrew M Davidoff

**Affiliations:** 1 ABC Cancer Center, Sur 136 No 116, Mexico City 01120, Mexico; 2 Department of Surgery, University of California, San Diego, 9300 Campus Point Drive, La Jolla, CA, 92093, USA; 3 Division of Surgery, St. Jude Children´s Research Hospital, 262 Danny Thomas Place, Memphis, TN 38105, USA; a https://orcid.org/0000-0003-0056-3455; b https://orcid.org/0000-0002-0953-0150; c https://orcid.org/0000-0001-7900-2794

**Keywords:** paediatric minimally invasive surgery, thoracic tumours, mediastinal masses, paediatric oncology, paediatric surgical oncology

## Abstract

The Role of Minimally Invasive Surgery (MIS) in Paediatric Mediastinal Masses and Thoracic Tumours. MIS has transformed paediatric surgical oncology. This chapter explores the pivotal role of MIS in managing thoracic and mediastinal tumours in children, emphasising diagnostic and therapeutic advancements. Video-assisted thoracoscopic surgery has demonstrated significant utility, allowing for precise tumour resections and reduced morbidity. The techniques' feasibility and efficacy are underscored across a range of tumour types, including thymic, neurogenic and germ cell tumours, with promising outcomes in both high-resource and low- and middle-income countries (LMICs). The chapter pretends to be a practical guide for surgeons treating children with mediastinal and thoracic tumours, describing types of neoplasms, diagnostic approaches and treatment principles and options, with particular focus on surgical nuances and intraoperative advice. Despite its benefits, the chapter highlights critical challenges, including the limitations posed by large, invasive tumours and resource constraints in LMICs. The role of MIS in pulmonary metastases, particularly in osteosarcoma, is also discussed, with a focus on the balance between minimally invasive approaches and open surgeries for complete resection. Key principles for patient selection, surgical planning and the integration of advanced imaging and technology are emphasised, aiming for optimal outcomes. The chapter also addresses contraindications, different surgical techniques, anaesthetic considerations and the importance of global collaboration to expand access to MIS. It concludes with a call for continued innovation and equitable distribution of minimally invasive technologies worldwide, ensuring that the benefits of these techniques are accessible to all children with cancer, irrespective of geographical and economic barriers.

## Introduction

Minimally invasive surgery (MIS) has become an essential component in paediatric surgical oncology, offering several advantages over open surgery, such as significant reductions in hospital length of stay, complication rates, intraoperative blood loss and transfusion requirements, without compromising oncological outcomes [1]. Among MIS techniques, video-assisted thoracoscopic surgery (VATS) stands out for its availability, versatility and utility in managing thoracic and mediastinal tumours in children. Robotic-assisted thoracoscopic surgery (RATS) is also gaining popularity. Diagnostic and therapeutic indications have expanded alongside a better understanding of cancer biology, enhanced imaging for staging, 3D modeling for preoperative planning, refinement of MIS technologies, fluorescence-guided surgery and improved paediatric surgical oncology expertise [2]. However, challenges remain in global implementation, particularly in low- and middle-income countries (LMICs), where access to care is often limited and high-technology solutions may not be available [3].

## MIS indications in paediatric solid tumours

MIS plays both a diagnostic and therapeutic role in the management of paediatric thoracic and mediastinal tumours.

### Diagnostic biopsies

VATS provides safe and minimally invasive access to thoracic tumours for tissue sampling, ensuring accurate histopathological diagnosis while reducing surgical morbidity [2]. It allows for safe, direct-vision biopsy of lung parenchyma and pleura, chest wall, diaphragm and mediastinal lesions. Diagnostic challenges often arise in distinguishing between benign (infectious or inflammatory) lesions and metastatic disease in the lung parenchyma, pleural surfaces and mediastinum. This is especially relevant in high-risk paediatric cancer patients, such as those with solid tumours of unfavourable biology that often metastasise to these sites. The ability to obtain adequate tissue samples for histological and immunophenotypic analyses has improved diagnostic accuracy and reduced the need for repeat procedures [4].

Patients undergoing hematopoietic stem cell transplantation are at significant risk of developing opportunistic infections, including fungal and parasitic lung infections, which may mimic malignant lesions on imaging [5]. Additionally, immunocompromised children receiving chemotherapy may develop atypical infections (tuberculosis, pneumocystis pneumonia or invasive aspergillosis), presenting as pulmonary nodules [5]. Prior lung infections can lead to granulomas or other structural changes that complicate imaging interpretation. These patients benefit greatly from VATS for diagnosis due to their poor healing capacity.

In patients with suspected or confirmed metastatic disease, MIS may also be used for staging and assessment of disease progression.

### Therapeutic resections

VATS is indicated for the resection of most localised tumours of the mediastinum, both anterior (e.g., germ cell tumours and thymomas) and posterior (e.g., neurogenic tumours), as well as selected primary early-stage tumours without significant invasion into adjacent structures. In select patients, MIS can be feasible for metastatic lung lesions as well [6]. For chest wall tumours, thoracoscopy can aid in tumour resection, guide incision planning and extent of resection.

## Feasibility and outcomes of MIS in mediastinal tumours in children

MIS, particularly VATS and RATS, has gained prominence in the management of mediastinal tumours in paediatric patients. These tumours, often located in anatomically complex regions, pose challenges for surgical access and safe resection. MIS has demonstrated advantages over traditional open approaches, including faster recovery, less pain and better cosmetic outcomes, with increasing evidence supporting its feasibility, safety and efficacy.

A recent meta-analysis, including 333 paediatric patients, found that VATS is successful in achieving complete tumour excision with low perioperative morbidity, as well as decreased length of stay, complication rate, intraoperative blood loss and transfusion requirements compared to open thoracotomy [2]. Complications occur in 2%–20% of cases, but are generally minor and amenable to non-operative management, such as transient pneumothorax or atelectasis [7]. For experienced surgeons, serious complications (vascular injury) occur in up to 10% of patients with neurogenic posterior mediastinal tumours and positive image-defined risk factors [8].

### Specific tumour types and outcomes

1. Thymic tumours: MIS is increasingly used for thymectomies in paediatric patients, especially in early-stage thymomas or thymic hyperplasia. Outcomes are excellent, with complete resection rates exceeding 95% in experienced centers [9].
2. Neurogenic tumours: Typically located at the posterior mediastinum, these are especially amenable to MIS due to their peripheral location, solid consistency, limited vascularity and frequent lack of involvement with vital structures (Figure 1a and b), especially in older children, since they tend to be well-differentiated. Of course, a large posterior mediastinal neuroblastoma encasing the aorta or with thoraco-abdominal extent is likely not a suitable candidate for an MIS approach. However, in well-selected cases, studies report VATS with excellent outcomes, low recurrence rates and minimal perioperative morbidity [10].
3. Teratomas and germ cell tumours: Mature teratomas and benign germ cell tumours in the anterior mediastinum are amenable to MIS resection [1]. Malignant or extensive germ cell tumours, involving adjacent vital structures [11] require careful selection, as incomplete resection will affect prognosis [11, 12].
4. Lymphomas: In addition to interventional radiology-guided core biopsies, VATS has become a useful technique for obtaining tissue samples in patients with suspected mediastinal lymphomas. The approach provides high diagnostic accuracy as more tissue can be obtained with minimal surgical trauma, facilitating timely initiation of systemic therapy [1, 13].

## Feasibility and outcomes of MIS in paediatric patients with lung metastases

Approximately 25% of children and adolescents with extracranial solid tumours present with metastases at diagnosis, and an additional 20% will develop metachronous pulmonary lesions during or after therapy [14]. The lungs are the most common site of metastatic spread across most paediatric solid tumours. While systemic therapy remains the cornerstone of treatment, long-term survival varies widely (20% to 70%) depending on tumour histology, biology and responsiveness to treatment [14]. Despite significant improvements in localised disease outcomes, prognosis remains poor for patients with metastatic tumours, particularly when lesions are refractory to chemotherapy or radiotherapy.

Surgical resection of pulmonary metastases serves both diagnostic and therapeutic purposes. It is most beneficial in tumours resistant to systemic therapies, where complete metastasectomy may offer a meaningful survival benefit. Conversely, in patients with disseminated, uncontrolled extra-pulmonary disease, the role of lung metastasectomy becomes less clear [15]. The decision to perform pulmonary metastasectomy depends on the tumour’s biology and its responsiveness to chemotherapy, radiation or targeted therapies.

### Surgical objectives for pulmonary metastasectomy

Surgical planning should distinguish between diagnostic and therapeutic intent. Diagnostic purposes aim to obtain one or two representative nodules using the least invasive approach. For therapeutic resections, the goal should be to achieve an R0 resection through localised, non-anatomic excisions (e.g., wedge resection or enucleation with a small margin of healthy tissue), aiming to maximise preservation of lung parenchyma. Segmentectomies, lobectomies or pneumonectomies are feasible through MIS, but are rarely necessary and reserved for specific circumstances (e.g., large, central lesions adjacent to the hilum, in patients with systemic disease control and no other metastatic sites) (Table 1).

A wide range of techniques has been described to localise deeper lesions and preserve maximal lung parenchyma. These include pre-operative marking with wires, coils or dyes and the use of intraoperative fluoroscopy, ultrasound or near infrared light [14] (Figure 2a–d).

Small, solitary, peripheral lung nodules (<1–2 cm), easy to visualise and resect, are particularly well-suited for thoracoscopic resection [16]. The ability to achieve negative surgical margins is a critical factor in determining the suitability of MIS. For solitary, peripheral lesions, recurrence rates and overall survival following MIS metastasectomy are similar to those achieved with open surgery, provided that negative margins are obtained [17].

For patients with bilateral disease, staged or simultaneous bilateral VATS with alternating one-lung ventilation can reduce the morbidity of formal thoracotomies [18] and allow for faster resumption of systemic chemotherapy. This approach is feasible when lesions are accessible via VATS and clear margins can be achieved. For patients with recurrent pulmonary metastases, VATS may offer a less invasive option for repeat resections, reducing postoperative pain and preserving pulmonary function [17].

## Particularities of MIS in metastatic osteosarcoma

Osteosarcoma is the most common solid tumour in children for which thoracic metastasectomy is considered. The role of thoracoscopic resection of lung metastases in patients with osteosarcoma is controversial. Complete resection of these nodules is critical for long-term survival. However, computed tomography (CT) scanning consistently underestimates the number of lung nodules in these patients and thoracoscopy, by the loss of tactile feedback, may not identify all lesions. One exception may be patients who completed chemotherapy and, during follow-up, present with solitary lung nodules on imaging. It has been shown that these particular patients are unlikely to have additional metastatic sites [19].

## Open versus thoracoscopic approach for pulmonary lesions in osteosarcoma

Patients diagnosed with osteosarcoma benefit from complete resection of all pulmonary metastases, as those achieving complete surgical remission may become long-term survivors [12]. To date, this is more feasible through open approaches, which allow for palpation and tactile feedback for detecting otherwise unidentifiable lesions by tomography.

Thoracoscopic and robot-assisted resections lack tactile feedback, making it challenging to intraoperatively locate small or intraparenchymal lesions. Currently, the role of minimally invasive techniques is limited to diagnostic biopsies of superficial nodules. The Children’s Oncology Group is conducting a protocol to validate the utility of thoracoscopic surgery in patients with metastatic osteosarcoma to the lung, limited to no more than four superficial lesions accessible via this approach [20] (Table 2).

Minimally invasive techniques may be considered for subsequent relapses of solitary pulmonary nodules diagnosed after completing therapy, as ipsilateral disease is unlikely to be found [19].

Patients diagnosed with osteosarcoma benefit from complete surgical resection of pulmonary metastases. This is more feasible through open approaches. However, additional studies on the utility of minimally invasive techniques are well justified. Surgery should be performed by an experienced surgical team, familiar with the surgical principles of osteosarcoma and must be effective, feasible and safe.

Non-surgical options such as stereotactic radiotherapy, radiofrequency ablation or cryotherapy may be considered as alternative options for patients who are not surgical candidates [21, 22].

## MIS contraindications

Tumour characteristics: Large or invasive tumours encasing vital structures such as the great vessels, esophagus or trachea, represent a relative contraindication for MIS, depending on the surgeon’s expertise. These may not be suitable for MIS due to technical difficulties in achieving a safe and complete resection [23]. Complex mediastinal tumours may take longer to excise during a minimally invasive approach when compared to open surgery, especially in centers with limited experience. The steep learning curve associated with MIS in mediastinal tumours underscores the need for specialised training and high-volume centers to optimise outcomes.

Regarding centrally located pulmonary metastases near critical structures, such as the pulmonary hilum or major vessels, are not ideal candidates for MIS due to the risk of incomplete resection or significant complications. A high level of expertise is required to take on this scenario safely.

Severe adhesions: Dense pleural or mediastinal adhesions from prior surgeries or infections can hinder the safe use of VATS [24].

Patient-specific factors: Severe cardiopulmonary compromise, which may preclude the use of single-lung ventilation, is a relative contraindication for thoracoscopic surgery [25]. Single-lung ventilation may be difficult to achieve in patients too small for a double-lumen endotracheal tube, although main-stem bronchus intubation with a single-lumen tube may suffice in these patients.

Individualised evaluation using imaging and multidisciplinary input is crucial for determining suitability for MIS.

## Surgical approach

### Types of minimally invasive approaches to the thorax

#### Thoracoscopy

One view port and two working ports are typical. Best for ≤4 superficial lesions. Preoperative lesion marking is useful for this approach since there is no tactile feedback. Specimens are extracted in a bag [16]. It is generally used for diagnosis, obtaining pleural fluid or performing incisional pleural or lung biopsies (Figure 3).

#### Video-assisted thoracoscopic surgery

A 30-degree 5 mm camera is placed through a thoraco-port at the 7th intercostal space, at the mid-axillary line. After visual inspection of the pleural cavity and detailed identification of the lesion to be resected, a small incision will allow for effective instrumentation. The mini-thoracotomy extends 3–4 cm long from the anterior axillary line towards the sternum, at the inferior crease of the pectoralis major muscle. The thorax is accessed at the 4th or 5th intercostal space, depending on the particular tumour location. Incisions can be extended anteriorly or posteriorly if needed for vascular control, tactile feedback or *en-block* specimen extraction. A small Alexis wound retractor lubricated with sterile gel facilitates instrument retraction of the lung, smooth instrumentation and extraction of the specimen in a retrieval bag (Figure 4). Pulmonary lesions can be resected using a stapler (Endo-GIA). Liberation of the inferior pulmonary ligament allows for lung mobilisation and extra-thoracic resection of lower, peripheral lesions [18].

#### Robotic-assisted thoracoscopic surgery

Similar to VATS but utilising a robotic system. It allows for enhanced dexterity and visualisation in confined spaces. However, the cost is high and its availability is very limited in LMICs. For surgeons comfortable performing VATS, robotics facilitates the procedure. For those without previous VATS experience, there may be a significant learning curve [26].

### Anaesthetic considerations

Anaesthesia management is a critical component of MIS in paediatric patients with thoracic tumours and mediastinal masses. The unique challenges posed by these tumours, combined with the physiological impacts of MIS techniques, require careful planning and expertise.

Airway compression and cardiopulmonary function must be assessed preoperatively. Patients with clinical or radiological evidence of airway compression require pulmonary function tests (VEF1). Both pulmonary and cardiac function play a separate role in anaesthetic risk. For patients with airway compression and risk of ventilatory collapse during induction anaesthesia, it is critical to maintain spontaneous ventilation and avoid the use of muscle relaxants until the airway is secured (Figure 5). Flexible bronchoscopy may be a useful adjunct for difficult airway management or to confirm airway patency. On the other end of the spectrum, patients at high risk of cardiovascular collapse may require extracorporeal membrane oxygenation to tolerate the procedure.

### Patient positioning

Patient positioning is dictated by tumour location. Rolls paced under the thorax for extension are useful and a mild head elevation benefits the cardiopulmonary function.

- Lateral decubitus: commonly used for unilateral thoracic tumours.
- Supine positioning: preferred for anterior mediastinal mass resection because it provides optimal surgical access, facilitates airway management and allows for safe cardiopulmonary monitoring and support.
- 45º angled decubitus: useful for posterior mediastinal lesions.

### Incision and trocar placement

Trocar placement varies based on tumour location, patient size and body habitus. It is important to consider that in thoracoscopy, the classic optimal triangulation used in abdominal MIS does not necessarily apply.

- For visualisation of the entire thoracic cavity, a 5 mm camera port placed at the 6th or 7th intercostal space, midaxillary line is convenient.
- Placement of the rest of the ports (or incisions) is determined by the initial thoracoscopic findings and preoperative images.
- For anterior mediastinal tumours, trocars are positioned at the subxiphoid area and the lateral chest wall to facilitate access and visualisation. The intercostal space for working ports is selected under direct visualisation, depending on the particular tumour anatomy.
- For localised posterior mediastinal tumours, VATS offers excellent visualisation and direct access. This approach uses a camera in the midaxillary port and a 4 cm antero-lateral thoracotomy at the pectoral crease, where access is feasible through the 4th or 5th intercostal space.

### Surgical technique

Key steps include:

- Ideally, single lung ventilation should be used as a collapsed lung aids in visualisation and tissue handling. However, dual lumen endotracheal tubes are not available in smaller sizes for younger patients. Alternatively, a bronchoscopy-guided selective intubation or bronchial blocker is effective.
- CO_2_ insufflation into the chest cavity to aid in lung compression can be used in fully thoracoscopic procedures. Close communication between the surgery and anaesthesia teams is essential.
- Using advanced instrumentation with surgical sealing energy devices and staplers are optimal to minimise blood loss.
- The oncologic principle of *en bloc* tumour resection for certain tumour histologies cannot be overstated, particularly in MIS.

### Tumour resection

Panoramic and close-up visualisation of the operative field to guide precise tumour dissection. As mentioned, an Alexis wound retractor on the anterolateral thoracotomy incision (slightly lubricated with sterile gel) allows for smoother instrumentation and direct views. The incision can be extended for critical structure control, tactile feedback and versatility if needed. Ensure adequate hemostasis during every step of the procedure.

Image and fluorescence-guided surgery may be used for helping to help identify inconspicuous lesions and ensure free margin resections. A hook wire can be placed preoperatively by interventional radiology under CT-guidance to mark intraparenchymal lesions but it tend to dislodge easily. Indocyanine green (ICG) and other fluorescent dyes are promising adjuncts currently under investigation. ICG can be injected IV (24–72 hours prior to the procedure, depending on histology) or directly intralesional, by interventional radiology or the surgeon immediately before the procedure. Tissue must be exposed to near infrared light for the ICG to fluoresce. Methylene blue is used intralesional, but in the author’s experience (JS), it tends to disperse through the tissues and avoid proper visualisation of nodules.

The specimen is extracted in a bag through a small thoracotomy incision and should be properly handled, marked and referenced.

A chest tube can be placed at the surgeons´ discretion. If used, it can be delivered through the camera port site.

Intercostal nerve blocks or radiofrequency rhizolysis reduce postoperative opioid consumption, rapid resumption of activity and shorten hospital length of stay.

### Tips and pitfalls

Tips:

- Careful patient selection and preoperative planning.
- Delicate tissue handling.
- Employing operative technology (proper visual capacity, energy devices and staplers to minimise blood loss) and visual aids (ICG, fluoroscopy or ultrasound) enhances tumour localisation.

Pitfalls:

- Know when to convert.
- Be prepared to recognise the distorted anatomy as the procedure advances. Inadequate exposure or excessive manipulation of tumours may lead to bleeding or tumour spillage. Bleeding can be very sudden and aggressive.
- Plan for prompt conversion to open thoracotomy and have the appropriate instruments available.

## Outcomes in LMICs

In LMICs, MIS for mediastinal and lung tumours remains underutilised due to limited access to advanced surgical tools and expertise. Additionally, patients tend to present with more advanced disease, often requiring open thoracotomy. However, as training programs and infrastructure improve, MIS is increasingly being adopted, showing promising outcomes even in resource-constrained settings. Recent studies highlight the importance of international collaboration to expand access to MIS for paediatric mediastinal tumours globally [26].

## MIS in global paediatric surgical oncology

MIS has the potential to bridge gaps in paediatric oncology outcomes worldwide. However, LMICs face several barriers:

- Limited access to advanced MIS equipment and trained personnel [3].
- High costs associated with procuring and maintaining MIS technologies.
- Challenges in perioperative care due to resource constraints.

Collaborative efforts, such as international training programs and the donation of MIS tools, are crucial to improving global equity in paediatric oncology care.

## Conclusion

The role of MIS in managing mediastinal and thoracic tumours is well-established in paediatric surgical oncology. Benefits include high resection rates (>90%), low complication rates and favourable oncological outcomes [8]. As technology evolves, indications continue to broaden. However, efforts are needed to ensure that these benefits are accessible globally, particularly in LMICs. Future research should focus on refining techniques, developing cost-effective solutions and evaluating long-term outcomes. Finally, careful patient selection is paramount, considering tumour size and histology, location, the patient's habitus and cardiopulmonary reserve.

## Conflicts of interest

All the authors declare that they have no conflicts of interest.

## Funding

All the authors declare no funding for this research.

## Figures and Tables

**Figure 1.** (a): Thoracoscopic view posterior mediastinal tumour. Thoracoscopic image depicting a neuroblastic posterior mediastinal tumour, at the right para-vertebral sulcus. A spherical lesion that extends over two vertebral bodies is evident. The lung is retracted medially (bottom right of the screen). The azygous vein can be seen cephalad and medial to the tumour, draining in the superior vena cava. Ribs and intercostal bundles are shown lateral of the lesion (left of the screen). (b): The lesion is resected with the use of an energy device (harmonic scalpel). VATS magnification aids in meticulous dissection.

**Figure 2.** (a): Preoperative marking. A paediatric patient with osteosarcoma and a small right-sided lung nodule is marked under CT guidance. Just before surgery, interventional radiology injects 0.1 mL of lipiodol into the nodule to allow fluoroscopic visualisation during thoracoscopy. (b): Post marking CT image of the patient demonstrates the contrast-enhanced nodule. (c): During surgery, fluoroscopy aids in localising the lung nodule for full thoracoscopic resection. (d): Intraoperative fluoroscopy image identifies the small, intra-parenchymal nodule. (a–d) are courtesy of Gloria Gonzalez, MD. Paediatric Transplant and Oncology Surgeon, Cleveland Clinic.

**Figure 3.** Surgical team setting. Surgical team positioning for effective minimally invasive access to the thorax and mediastinum

**Figure 4.** VATS access setting. Operative view of access in VATS. A camera is placed through a thoraco-port on the 7th intercostal space, at the mid-axillary line. The mini-thoracotomy extends 3–4 cm long from the anterior axillary line towards the sternum, usually at the fourth, fifth or sixth intercostal space, depending on the anatomic location of the mass. An Alexis wound retractor lubricated with sterile gel is placed into the mini thoracotomy in order to facilitate retraction of the lung using smooth instrumentation, and specimen extraction in a retrieval bag.

**Figure 5.** Tracheal compression. Contrast-enhanced axial CT image of a 6-year-old girl with a large tumour at the upper right posterior mediastinum. The lesion crosses the midline over the anterior surface of the vertebral body, at the level of the manubrium. Both, arterial and venous mediastinal great vessels are selectively enhanced with contrast. The trachea is severely compressed by the tumour (Blue arrow). This patient is at high risk of ventilatory collapse during the induction of anaesthesia.

**Table 1.** 

Principles for pulmonary metastasectomy
Consider tumour histology and biological behaviour. Accurate diagnosis of pulmonary lesions may avoid unnecessary toxic therapies. The number of metastases or disease-free interval are not contraindications for metastasectomy. Bilateral synchronous or staged resections are well tolerated. Negative margins are essential; localised resections should be performed to preserve as much adequate lung volume as possible [16].

**Table 2.** 

Basic principles for osteosarcoma surgery.
a) Primary tumour control has been achieved (or is achievable). b) There is no clinical or radiological evidence of unresectable extra thoracic metastases. c) Postoperative pulmonary function is anticipated to be adequate. d) The patient’s clinical condition and functional status are acceptable. e) Clinical and radiological findings indicate that complete resection is feasible.

## References

[1] Fukuta A, Kawakubo N, Souzaki R (2021). Endoscopic surgical approach for pediatric solid tumors that permits complete curability and exhibits cosmetic advantages. J Laparoendosc Adv Surg Tech A.

[2] Noleto da Nobrega Oliveira RE, Salvador ICMC (2025). Comparison of video-assisted surgery and open surgery for mediastinal tumor resection in pediatric population: a systematic review and meta-analysis. J Pediatr Surg.

[3] Lukish JR, Ellis-Davy J (2022). A novel low cost minimally invasive surgical system allows for the translation of modern pediatric surgical technology to low- and middle-income nations. J Pediatr Surg.

[4] Rakislova N, Fernandes F, Lovane L (2019). Standardization of minimally invasive tissue sampling specimen collection and pathology training for the child health and mortality prevention surveillance network. Clin Infect Dis.

[5] Otto WR, Green AM (2020). Fungal infections in children with haematologic malignancies and stem cell transplant recipients. Br J Haematol.

[6] Abdelhafeez A, Ortega-Laureano L, Murphy AJ (2019). Minimally invasive surgery in pediatric surgical oncology: practice evolution at a contemporary single-center institution and a guideline proposal for a randomized controlled study. J Laparoendosc Adv Surg Tech A.

[7] Svetanoff WJ, Carter M, Diefenbach KA (2024). Robotic-assisted pediatric thoracic and abdominal tumor resection: an initial multi-center review. J Pediatr Surg.

[8] Burt BM, Yao X, Shrager J (2017). Determinants of complete resection of thymoma by minimally invasive and open thymectomy: analysis of an international registry. J Thorac Oncol.

[9] Irtan S, Brisse HJ, Minard-Colin V (2015). Minimally invasive surgery of neuroblastic tumors in children: indications depend on anatomical location and image-defined risk factors. Pediatr Blood Cancer.

[10] Saikia J, Deo SVS, Bhoriwal S (2020). Video assisted thoracoscopic surgery in paediatric mediastinal tumors. Mediastinum.

[11] Tan Z, Zhang J, Shu Q (2023). Robotic-assisted resection for mediastinal tumors. Pediatric Robotic Surgery.

[12] Fuchs J (2015). The role of minimally invasive surgery in pediatric solid tumors. Pediatr Surg Int.

[13] Massone PP, Lequaglie C, Magnani B (2003). The real impact and usefulness of video-assisted thoracoscopic surgery in the diagnosis and therapy of clinical lymphadenopathies of the mediastinum. Ann Surg Oncol.

[14] Rodriguez M, Milla L, Wee JO (2022). The role of minimally invasive surgery in the management of giant mediastinal tumors: a narrative review. Mediastinum.

[15] Petrella F, Diotti C, Rimessi A (2017). Pulmonary metastasectomy: an overview. J Thorac Dis.

[16] Croteau NJ, Heaton TE (2019). Pulmonary metastasectomy in pediatric solid tumors. Children (Basel).

[17] Heaton TE, Davidoff AM (2016). Surgical treatment of pulmonary metastases in pediatric solid tumors. Semin Pediatr Surg.

[18] Phelps HM, Lovvorn HN (2018). Minimally invasive surgery in pediatric surgical oncology. Children (Basel).

[19] Fernandez-Pineda I, Daw NC, McCarville B (2012). Patients with osteosarcoma with a single pulmonary nodule on computed tomography: a single-institution experience. J Pediatr Surg.

[20] Aljubran AH, Griffin A, Pintilie M (2009). Osteosarcoma in adolescents and adults: survival analysis with and without lung metastases. Ann Oncol.

[21] Teke ME, Saif A, Sarvestani AL (2022). A phase III, randomized, controlled trial comparing open versus thoracoscopic management of pulmonary metastases in patients with osteosarcoma. Ann Surg Oncol.

[22] Longhi A, Marrari A, Tetta C (2024). The critical role of stereotactic body radiation therapy in multimodal treatment of lung metastasis from bone and soft tissue sarcomas. Cancers (Basel).

[23] Yevich S, Gaspar N, Tselikas L (2016). Percutaneous computed tomography-guided thermal ablation of pulmonary osteosarcoma metastases in children. Ann Surg Oncol.

[24] Li SJ, Zhou K, Wu YM (2018). Presence of pleural adhesions can predict conversion to thoracotomy and postoperative surgical complications in patients undergoing video-assisted thoracoscopic lung cancer lobectomy. J Thorac Dis.

[25] Hanna JM, Berry MF, D'Amico TA (2013). Contraindications of video-assisted thoracoscopic surgical lobectomy and determinants of conversion to open. J Thorac Dis.

[26] Navarrete Arellano M, Garibay González F (2019). Robot-assisted laparoscopic and thoracoscopic surgery: prospective series of 186 pediatric surgeries. Front Pediatr.

# Other: Minimally invasive surgery guidelines in paediatric surgical oncology - role of MIS in fertility preservation

**DOI:** 10.3332/ecancer.2025.2028

**Published:** 2025-10-23

**Authors:** Marianna Cornet, Julien Grosman, Aurore Pire, Sabine Sarnacki

**Affiliations:** Department of Paediatric Surgery, Urology and Transplantation, Necker Enfants-Malades Hospital, AP-HP, University Paris Cité, Paris 75015, France

**Keywords:** fertility preservation, minimally invasive surgery, gonadotoxicity, ovarian sparing surgery, ovarian transposition, uterine transposition, cryopreservation

## Abstract

Childhood cancers represent approximately 1% of all malignancies, with improved therapeutic strategies leading to an 80% long-term survival rate. However, these advancements come with potential long-term sequelae, among which fertility impairment is a major concern. Gonadotoxic treatments, including chemotherapy, radiotherapy and mutilating surgery, significantly impact reproductive potential, necessitating fertility preservation strategies. Minimally invasive surgery (MIS) plays a crucial role in preserving fertility in paediatric patients. Ovarian and genital tract-sparing surgery should be prioritised for benign ovarian tumours, which constitute 90% of childhood ovarian lesions, to avoid unnecessary loss of ovarian reserve. Ovarian transposition is recommended for patients requiring pelvic radiotherapy, relocating the ovaries outside the radiation field to mitigate ovarian damage. Additionally, uterine transposition has been explored to protect reproductive organs from radiation exposure. Ovarian tissue cryopreservation remains a promising option, particularly for prepubertal patients undergoing gonadotoxic treatments. Cryopreserved ovarian fragments can later be used for autografting or *in vitro* maturation, though the risk of malignant cell transmission remains a challenge. MIS contraindications are limited, primarily related to tumour size and the risk of rupture during laparoscopic procedures. A multidisciplinary approach involving oncologists, surgeons, radiotherapists and fertility specialists is essential for optimising outcomes. This chapter discusses the indications, techniques and challenges associated with MIS in fertility preservation, emphasising its role in ensuring reproductive potential while maintaining oncological safety in paediatric cancer patients.

## Introduction

Childhood cancers currently account for approximately 1% of all malignancies. Advances in the understanding of paediatric cancer biology have significantly improved therapeutic approaches, leading to an 80% long-term overall survival rate, primarily through intensified targeted chemotherapy and/or radiotherapy [1]. However, these advances come at the cost of potential long-term adverse effects [2].

Among these, fertility impairment is a major concern, representing one of the most significant long-term sequelae of paediatric cancer treatment. It carries the risk of reduced reproductive potential and can have profound psychological repercussions. Fertility disorders may result from gonadotoxic treatments, such as chemotherapy and radiotherapy, as well as from mutilating surgical procedures affecting reproductive organs [3].

The paediatric oncologic surgeon, therefore, plays a crucial role in fertility preservation for patients undergoing cancer treatment. The goal is to minimise long-term sequelae in these young patients who have their whole lives ahead of them. In this context, minimally invasive surgery (MIS) is essential, as it helps reduce visible scarring and ensures a faster recovery, which is particularly important to prevent delays in treatment initiation. Ensuring timely access to treatment is critical for optimising outcomes, allowing patients to start their therapies without unnecessary postponements.

### Mechanisms of induced infertility

#### In Females

Alkylating agents, such as cyclophosphamide, which are widely used—particularly in high-dose conditioning chemotherapy—are among the most gonadotoxic and frequently lead to ovarian failure.

Ionising radiation affects both dividing and non-dividing cells. As with chemotherapy, the impact of radiotherapy on ovarian function depends on patient age, radiation field, daily dose and cumulative dose received. Permanent ovarian failure has been associated with radiation doses of 10–20 Gy in children and 4–6 Gy in adults. Moreover, uterine irradiation during childhood is known to increase the risk of preterm birth and low birth weight offspring [1, 4, 5].

Dose fractionation and intensity-modulated radiation therapy in external beam radiation therapy (EBRT) have been associated with reduced ovarian damage due to the potential for cellular repair between fractions [6, 7]. Pelvic brachytherapy, which is used mostly for urogenital rhabdomyosarcomas (RMS) and other sarcomas, is expected to be less toxic than EBRT but may lead to vaginal stenosis, which can impair fertility [8]. Additionally, irradiation of the hypothalamic-pituitary-adrenal (HPA) axis at doses ≥30 Gy can result in gonadotropic failure, although ovarian reserve is generally preserved [9].

Finally, mutilating surgery, partial resection of the genital tract and/or ovaries or even any kind of pelvic surgery, especially since it is associated with irradiation, may impair fertility potential.

#### In Males

Prepubertal gonads are highly susceptible to the gonadotoxic effects of chemotherapy administered during childhood. Alkylating agents can impair spermatogenesis, though testosterone secretion is usually preserved.

In contrast, radiation therapy affects both spermatogenesis and testosterone production. Spermatogonia are sensitive to doses as low as 0.15 Gy and are completely eradicated at doses ≥2 Gy. Prepubertal males are more vulnerable to the effects of irradiation on spermatogenesis and Leydig cell function compared to adolescents and adults. Notably, unlike females, fractionated radiation doses induce higher gonadal toxicity than a single exposure.

Irradiation of the HPA axis at doses ≥25–30 Gy can cause hypogonadotropic hypogonadism, leading to pubertal delay, sexual dysfunction and infertility, with a dose-dependent effect [10]. Childhood cancer survivors show a 50% decrease in testicular descent compared to the general population [11]. Additionally, long-term follow-up studies have reported a significant increase in fertility disorders (46% in survivors versus 17.5% in siblings; RR = 2.64, 95% CI 1.88–3.70, *p* < 0.001) with documented sperm abnormalities in a cohort of 1,600 childhood cancer survivors [12].

Regarding fertility preservation in males, MIS does not have a role; indeed, testicular cryopreservation, testicular transposition or testicular sparing surgery are performed exclusively through an open surgical approach. In this chapter, we will present the various aspects of MIS for fertility preservation in females paediatric patients undergoing cancer treatment.

## MIS indications

### Ovarian and genital tract sparing surgery

In girls, ovarian sparing surgery is mandatory to preserve fertility in case of benign ovarian tumours, which represent around 90% of childhood ovarian lesions (benign germ cell or benign epithelial tumours). Indeed, 13% of ovarian teratomas are bilateral (either synchronous or metachronous) [13] and unilateral ovariectomy leads to depletion of the total number of primordial follicles and, therefore, might increase the risk of premature or early menopause, although not clearly demonstrated in children. If in adults evidence indicates that having a single ovary does not reduce the fertility potential (with normal live birth rates, spontaneously or after assisted reproductive techniques), evidence from long-term studies in children is missing [14, 15]. In the absence of evidence in the paediatric population, ovarian sparing surgery should always be considered.

In everyday practice, deciding whether to spare the ovary because the lesion seems benign or to perform a total ovariectomy because signs of malignancy are present is not always straightforward [16]. Two different situations should be considered:

1. In asymptomatic (painless) girls, the surgeon has enough time to perform reliable imaging (ensuring that the tumour does not show any sign of malignancy) and to dose tumour markers alpha-fetoprotein, human chorionic gonadotrophin, Inhibin B, anti-Mullerian hormone and Calcemia. In these situations, laparoscopic exploration is recommended as the first step of the procedure. The primary goal of laparoscopy is to better appreciate the location and nature of the lesion, and in malignant cases, to precisely stage the disease. However, laparoscopic ovarian tumour resection is not recommended by the present authors and laparotomy via a supra-pubic approach remains the preferred method. The rationale for open surgery in paediatric ovarian tumours includes the potential presence of malignancy even in cystic lesions (e.g., rare cases of juvenile granulosa cell tumours) or those appearing as mature teratomas, which may contain both mature and immature components, where intraoperative rupture would significantly worsen prognosis. Additionally, the open approach remains the safer option for maximising the preservation of healthy ovarian tissue when ovarian-sparing surgery is chosen for a tumour with all the characteristics of a benign lesion. The wish to favour aesthetic considerations in the treatment of ovarian masses in children could then lead to a loss of oncological control, which seems unacceptable considering that the classic treatment proposes a supra-pubic approach that results in the same post-operative course.
2. When the patient is complaining of acute pain due to adnexal torsion or ovarian rupture, the best approach is to perform a laparoscopic exploration in an emergency. If ovarian rupture is diagnosed, peritoneal inspection, sampling of ascites are the rule. Complete ovariectomy or adnexectomy in the same operative time should be discussed only if the ovary and/or the peritoneum indicate malignant features. If adnexal torsion is seen, detorsion is recommended as it allows to approach the potential underlying diagnosis (benign or malignant tumour) with imaging and markers dosage in the post-operative period.

Preservation of the genital tract should be considered when planning surgical interventions for tumours involving these anatomical structures. The various pathological entities encountered include: RMS of the vagina, cervix or uterus; malignant germ cell tumours (GCT) of the vagina; and clear cell adenocarcinoma, which is typically diagnosed later in life. The prognosis of these tumours depends on achieving local tumour control, which is now managed with less radical surgical approaches compared to those employed in previous decades. For urogenital RMS, treatment may be accomplished without surgical intervention when complete remission is achieved following chemotherapy or it may be supplemented with localised brachytherapy to avoid mutilating surgery, the latter of which is now exceptionally rare [17, 18]. In cases of vaginal GCT, surgical resection of the primary tumour site remains essential; however, the efficacy of neoadjuvant chemotherapy often precludes the need for mutilating surgery, enabling a more conservative approach such as partial trachelectomy.

### Ovarian transposition

Ovarian transposition was the first procedure proposed to preserve fertility in girls with cancer and is indicated for patients with tumours requiring pelvic radiation of 42–58.4 Gy, much higher doses than those that can induce loss of ovarian function (4–20 Gy) [5]. The ovaries are very radiosensitive organs. The extent of damage depends on the dose of radiotherapy that reaches the ovaries, the age of the girl at the time of treatment and the associated drugs used for chemotherapy. Doses of 10–20 Gy in children and 4–6 Gy in adults are associated with permanent ovarian failure [19].

Preliminary ovarian transposition should be considered for all children with tumours requiring radiotherapy implicating part of the pelvis. RMS of the bladder, vagina or uterus or soft tissue or pelvic bone sarcomas, such as Ewing’s sarcoma, are the main indications for ovarian transposition in children [5, 6, 20].

Ovarian transposition is designed to place the ovaries outside of the irradiation field. Detailed analysis of the initial pelvic MRI should be done to ensure that the tumour does not involve the ovarian region.

The planning target volume should be precisely defined by the surgeon and the radiotherapist before ovarian transposition.

### Uterine transposition

In some cases, radiation is an important complementary treatment. But the area can involve the pelvic region and result in a deleterious effect to fertility, which may affect the uterus and ovaries. The uterine and the adnexal, transposition to an upper abdominal region during the radiation therapy may protect these organs with security. Some cases are described in adult women [21, 22], and a case in a pre-pubertal girl was described with successful [23].

### Ovarian tissue cryopreservation

Main indications nowadays are represented by myeloablation before bone marrow or stem cell transplantation, total body irradiation and high-dose chemotherapy with alkylating agents [24]. There is some controversy about the amount of ovarian tissue to retrieve, as some teams propose to do a partial ovariectomy or to harvest only the cortex. Since follicular loss can reach up to 65% only due to the ischemia hit [25], it seems for many groups essential to collect an entire ovary in younger children in order to get enough tissue or cells for the future pregnancy project. It has to be noted that in most of the team the ovarian tissue is more used for fertility than for puberty restauration [26, 27].

Cryopreserved ovarian fragments might further be used for either autografting or *in vitro* maturation of primordial follicles. Since Donnez *et al* [28, 29] and Meirow *et al* [30] published the first 2 pregnancies obtained after autograft of ovarian cortex, more than 200 livebirths have been documented in the literature after autologous grafting of previously cryopreserved adult ovarian tissue. The ovarian function is restored after 4 months following grafting and 23%–37% of these women will have pregnancies. In prepubertal patients, results are not assessable, as most of these patients have not achieved the age of parental desire. Poirot *et al* [31] confirmed that induction of puberty was efficient by heterotopic autografts of ovarian fragments. In 2015, Demeestere *et al* [32] reported the first pregnancy after cryopreservation at a paediatric age. The patient, who required myeloablation before bone marrow transplantation for sickle cell anemia, was 14 years old but premenarchal at the age of cryopreservation. She gave birth 13 years later to a normal child, 2 years after bilateral autografting of ovarian fragments and natural conception. Besides this publication, no cohort studies or even isolated cases of livebirths after prepuberty ovarian tissue cryopreservation for malignancy have been reported [33].

A major problem raised by autografting of previously cryopreserved ovary is the risk of reintroduction of the primary disease in the patient’s organism, especially when the primary disease is likely to give metastasis to the gonads, such as leukaemia or lymphoma. Shaw *et al* [34] managed to prove, in mice models, that cryopreserved ovarian tissue samples from donors with lymphoma can transmit cancer to grafted recipients. Recent studies showed that the risk is highest in leukaemia patients, moderate in gastrointestinal cancers and low in breast cancer, sarcomas, gynecological cancers, Hodgkin’s and non-Hodgkin’s lymphomas [35, 36]. A way to avoid this problem will be the *in vitro* culture of primordial follicles harvested from the cryopreserved tissue, a technique which is currently still experimental [37].

## MIS contraindications

There are very few contraindications to MIS concerning fertility preservation. In the case of a large ovarian tumour extending beyond the umbilicus, initiating surgery with an open laparoscopy through the umbilical approach is contraindicated due to the risk of tumour rupture during trocar insertion. It is recommended to perform lesion resection via laparotomy as the first step.

For other fertility preservation techniques, the only contraindication is the presence of extensive adhesions preventing the feasibility of laparoscopy.

## Surgical approach

### Preoperative workup

- Blood tests: Complete blood count and coagulation profile are required. If cryopreservation is planned, viral serologies (HIV, hepatitis B, hepatitis C and TPHA/VDRL) should be obtained.
- Imaging: Pelvic MRI is requested to assess ovarian tumours. In cases of gonadal transposition for radiation protection, collaboration with the radiotherapy team is essential to ensure optimal gonadal repositioning outside the radiation field. For ovarian tissue preservation, imaging is not required.

Given the broad range of fertility preservation strategies, which depend on age, tumour type and treatment plan, multidisciplinary board discussions are highly recommended. These should include medical oncologists, radiation oncologists and fertility preservation specialists to determine the most appropriate approach.

A detailed informed consent should be provided and discussed with the patient and their parents to ensure a comprehensive understanding and shared decision-making.

### Patient position

The patient is positioned supine and in Trendelenburg position, which causes the intestine to fall into the upper part of the abdomen to free the pelvis and enable visualisation of the genital tract. The arms are placed alongside the body. The bladder is emptied with a transurethral Foley catheter [5, 38].

### Trocar sites

An umbilical trocar is inserted either via an infra-umbilical incision or directly through the umbilicus. Carbon dioxide is insufflated at a pressure of 8 to 12 mm Hg, dependent on the patient’s age and weight. Two working trocars (3–5 mm) are inserted under direct vision into the right and left flank, almost as the same level as the umbilicus, depending on the child’s age. The smaller the child, the higher the trocars should be placed on the abdomen to ensure a better working space. A 0° or 30° angled camera can be used.

### Surgical technique

#### Ovarian tumours

As described above, the goal of laparoscopy is to better assess the location and nature of the lesion. The supramesocolic area and anterior parietal peritoneum should be visualised with biopsies taken from any suspicious areas. A sample of ascitic fluid or peritoneal washings should be collected for cytological analysis if no ascites is present. Examination of abdominal organs, particularly the diaphragmatic domes, is essential. Omentectomy is recommended if the omentum, or any part of it, appears abnormal. Pelvic and retroperitoneal lymph nodes should be carefully evaluated, with biopsies of any abnormal nodes. The contralateral ovary should be assessed.

The second step of the procedure involves laparotomy.

If the tumour is large and extends beyond the umbilicus, peritoneal exploration via laparoscopy should be performed after open surgery.

## Ovarian transposition

Various sites for ovarian transposition can be considered. In case of a midline irradiation field (urogenital tumours or medulloblastoma), both ovaries are usually placed away from the midline, laterally in the paracolic gutters or laterally and anteriorly near the inguinal ring (bilateral ovarian transposition). In case of a lateral tumour (RMS or Ewing’s sarcoma), the compromised ovary is placed on the opposite site of the tumour (unilateral ovarian transposition). In some cases of Hodgkin’s lymphoma, when the irradiation field implicates the bilateral iliac chains and inguinal regions, the ovaries are placed in line with the iliac crests (bilateral ovarian transposition) [39].

In most of the cases, it is not necessary to section any ovarian ligament. In prepubertal girls, the ovaries are located higher in the pelvis and the ligaments are more stretchable than in adult women [38]. The blood supply to the ovaries should be carefully preserved, especially the ovarian vessels should be examined to ensure the absence of any kinking or direct injuries [40]. The ovaries are grasped with atraumatic forceps and mobilised above the iliac crest level (in the case of midline irradiation field) as high as possible without any dissection or division of the ovarian ligaments or of the fallopian tube. The ovaries are sutured to the peritoneum with resorbable or non-resorbable suture material and are marked by metallic clips to make them visible on imaging before the initiation of EBRT.

In the case of pelvic brachytherapy, ovarian transposition is only needed for a short time. The ovaries can then be sutured to the anterior abdominal wall by a transfixing stitch of non-resorbable suture material, knotted on the outside of the patient on a pledget. The stitch knotted on the outside holding the ovary to the abdominal wall is removed at the end of brachytherapy, to return the ovaries to their normal position without the need for reoperation [38].

Whatever technique is used, the surgical procedure should be performed as close to the time of radiation treatment as possible, due to the risk of remigration of the ovaries [41].

## Ovarian harvesting for cryopreservation

The ovary is grasped with atraumatic forceps. Hemostasis is performed with bipolar hemostatic forceps, and the mesovarium is cut with scissors. The ovary is placed within a bag and removed via the umbilical trocar. The ovary is then immediately placed in a transport medium and sent to the reproductive biology unit to be cryopreserved.

After isolation and fragmentation, ovarian cortex fragments are slowly frozen in an automated freezer down to the temperature of liquid nitrogen, in which they are stored. Histological analysis of some fragments is mandatory as it allows searching for malignant cells in both cortex and medulla, particularly in tumours with potential ovarian spread (hematological cancer and neuroblastoma) and also to estimate the follicular wealth of the tissue, notably in case of previous gonadotoxic treatment.

### Tips and pitfalls

1. For ovarian tumour surgery, a thorough preoperative assessment is essential. In the case of a malignant tumour, ovarian preservation is not possible. In the case of a benign tumour, caution is required if the tumour is large to avoid rupture during exploratory laparoscopy or during detorsion. To minimise the risk of tumour rupture as much as possible, the authors of this chapter recommend performing laparotomy for ovarian sparing surgery. In large cystic tumours, in cases where a benign tumour is highly suspected, the cyst is first covered with a sterilised surgical sheet applied with quick-drying glue [42] and then punctured using a 5 mm laparoscopic trocar connected to suction. Once the cyst(s) are drained, the ovary can be exteriorised and a suture is placed as the trocar is removed. The cyst resection surgery can then be performed with protection of the operative field.
2. If the tumour is large and extends beyond the umbilicus, peritoneal exploration via laparoscopy should be performed after open surgery.
3. Proper patient positioning is crucial, with the placement of a urinary catheter to ensure that the bladder remains empty throughout the procedure.
4. Regarding ovarian tissue cryopreservation, it is important in prepubertal girls to remove an entire ovary to avoid damaging the ovarian tissue. Atraumatic forceps should be used and the use of energy-based instruments should be minimised to avoid damaging the ovarian tissue.

## Conclusion

Fertility preservation in paediatric oncology has become a crucial aspect of patient care, given the significant gonadotoxic effects of chemotherapy, radiotherapy and certain surgical interventions. MIS plays an essential role in reducing treatment-related morbidity while optimising reproductive outcomes. Techniques such as ovarian and uterine transposition, ovarian sparing surgery and ovarian tissue cryopreservation have shown promising results in protecting future fertility, particularly when applied in a timely and well-coordinated manner.

A multidisciplinary approach involving oncologists, surgeons, reproductive specialists and radiotherapists is essential to tailor fertility preservation strategies to each patient's needs.

Future research should focus on improving fertility preservation techniques, optimising cryopreservation protocols and enhancing post-treatment fertility restoration options. As survival rates in childhood cancer continue to improve, ensuring the quality of life of survivors—including their reproductive potential—should remain a top priority in paediatric oncology.

## Conflicts of interest

No conflicts of interest.

## Funding

No funding declaration.

## References

[1] Anderson RA, Mitchell RT, Kelsey TW (2015). Cancer treatment and gonadal function: experimental and established strategies for fertility preservation in children and young adults. Lancet Diabetes Endocrinol.

[2] Phillips SM, Padgett LS, Leisenring WM (2015). Survivors of childhood cancer in the United States: prevalence and burden of morbidity. Cancer Epidemiol Prev Biomark.

[3] Raffoul L, Capito C, Sarnacki S (2016). Fertility considerations and the pediatric oncology patient. Seminars in Pediatric Surgery.

[4] Isachenko V, Todorov P, Isachenko E (2016). Cryopreservation and xenografting of human ovarian fragments: medulla decreases the phosphatidylserine translocation rate. Reprod Biol Endocrinol.

[5] Irtan S, Orbach D, Helfre S (2013). Ovarian transposition in prepubescent and adolescent girls with cancer. Lancet Oncol.

[6] Thibaud E, Rodriguez-Macias K, Trivin C (1998). Ovarian function after bone marrow transplantation during childhood. Bone Marrow Transplant.

[7] Presti AL, Ruvolo G, Gancitano RA (2004). Ovarian function following radiation and chemotherapy for cancer. Eur J Obstet Gynecol Reprod Biol.

[8] Levy A, Martelli H, Fayech C (2015). Late toxicity of brachytherapy after female genital tract tumors treated during childhood: prospective evaluation with a long-term follow-up. Radiother Oncol.

[9] Sudour-Bonnange H, Tabone MD, Thomas-Teinturier C (2013). Fertility preservation in children and teenagers with cancer. Bull Cancer (Paris).

[10] Sathyapalan T, Dixit S (2012). Radiotherapy-induced hypopituitarism: a review. Expert Rev Anticancer Ther.

[11] Green DM, Kawashima T, Stovall M (2010). Fertility of male survivors of childhood cancer: a report from the childhood cancer survivor study. J Clin Oncol.

[12] Wasilewski-Masker K, Seidel KD, Leisenring W (2014). Male infertility in long-term survivors of pediatric cancer: a report from the childhood cancer survivor study. J Cancer Surviv.

[13] Taskinen S, Urtane A, Fagerholm R (2014). Metachronous benign ovarian tumors are not uncommon in children. J Pediatr Surg.

[14] Bellati F, Ruscito I, Gasparri ML (2014). Effects of unilateral ovariectomy on female fertility outcome. Arch Gynecol Obstet.

[15] Zhai A, Axt J, Hamilton EC (2012). Assessing gonadal function after childhood ovarian surgery. J Pediatr Surg.

[16] Sarnacki S, Brisse H (2011). Surgery of ovarian tumors in children. Horm Res Paediatr.

[17] Martelli H, Oberlin O, Rey A (1999). Conservative treatment for girls with nonmetastatic rhabdomyosarcoma of the genital tract: a report from the Study Committee of the International Society of Pediatric Oncology. J Clin Oncol.

[18] Magné N, Oberlin O, Martelli H (2008). Vulval and vaginal rhabdomyosarcoma in children: update and reappraisal of Institut Gustave Roussy brachytherapy experience. Int J Radiat Oncol Biol Phys.

[19] Chemaitilly W, Mertens AC, Mitby P (2006). Acute ovarian failure in the childhood cancer survivor study. J Clin Endocrinol Metab.

[20] Potratz J, Dirksen U, Jürgens H (2012). Ewing sarcoma: clinical state-of-the-art. Pediatr Hematol Oncol.

[21] Ribeiro R, Rebolho JC, Tsumanuma FK (2017). Uterine transposition: technique and a case report. Fertil Steril.

[22] Ribeiro R, Anselmi MC, Schneider GA (2023). First live birth after uterine transposition. Fertil Steril.

[23] Vieira MA, Vieira AGS, Fonseca DSL (2021). Uterine transposition in a pre-pubertal patient. Int J Gynecol Cancer.

[24] Wallace WHB, Anderson RA, Irvine DS (2005). Fertility preservation for young patients with cancer: who is at risk and what can be offered?. Lancet Oncol.

[25] Sauvat F, Bouilly J, Capito C (2013). Ovarian function is restored after grafting of cryopreserved immature ovary in ewes. FASEB J.

[26] Poirot C, Brugieres L, Yakouben K (2019). Ovarian tissue cryopreservation for fertility preservation in 418 girls and adolescents up to 15 years of age facing highly gonadotoxic treatment. Twenty years of experience at a single center. Acta Obstet Gynecol Scand.

[27] Poirot C, Schubert B (2011). Fertility preservation in prepubertal children. Méd Reprod.

[28] Donnez J, Dolmans MM, Demylle D (2004). Livebirth after orthotopic transplantation of cryopreserved ovarian tissue. Lancet.

[29] Donnez J, Dolmans MM, Pellicer A (2015). Fertility preservation for age-related fertility decline. Lancet.

[30] Meirow D, Levron J, Eldar-Geva T (2005). Pregnancy after transplantation of cryopreserved ovarian tissue in a patient with ovarian failure after chemotherapy. N Engl J Med.

[31] Poirot C, Abirached F, Prades M (2012). Induction of puberty by autograft of cryopreserved ovarian tissue. Lancet.

[32] Demeestere I, Simon P, Dedeken L (2015). Live birth after autograft of ovarian tissue cryopreserved during childhood. Hum Reprod.

[33] Mulder RL, Font-Gonzalez A, Hudson MM (2021). Fertility preservation for female patients with childhood, adolescent, and young adult cancer: recommendations from the PanCareLIFE consortium and the International Late Effects of Childhood Cancer Guideline Harmonization Group. Lancet Oncol.

[34] Shaw JM, Bowles J, Koopman P (1996). Ovary and ovulation: fresh and cryopreserved ovarian tissue samples from donors with lymphoma transmit the cancer to graft recipients. Hum Reprod.

[35] Rosendahl M, Greve T, Andersen CY (2013). The safety of transplanting cryopreserved ovarian tissue in cancer patients: a review of the literature. J Assist Reprod Genet.

[36] Greve T, Wielenga VT, Grauslund M (2013). Ovarian tissue cryopreserved for fertility preservation from patients with Ewing or other sarcomas appear to have no tumour cell contamination. Eur J Cancer.

[37] Soares M, Saussoy P, Maskens M (2017). Eliminating malignant cells from cryopreserved ovarian tissue is possible in leukaemia patients. Br J Haematol.

[38] de Lambert G, Haie-Meder C, Guérin F (2014). A new surgical approach of temporary ovarian transposition for children undergoing brachytherapy: technical assessment and dose evaluation. J Pediatr Surg.

[39] Scott SM, Schlaff W (2005). Laparoscopic medial oophoropexy prior to radiation therapy in an adolescent with Hodgkin’s disease. J Pediatr Adolesc Gynecol.

[40] Hwang JH, Yoo HJ, Park SH (2012). Association between the location of transposed ovary and ovarian function in patients with uterine cervical cancer treated with (postoperative or primary) pelvic radiotherapy. Fertil Steril.

[41] Oktay K, Harvey BE, Partridge AH (2018). Fertility preservation in patients with cancer: ASCO clinical practice guideline update. J Clin Oncol.

[42] Watanabe E, Tanaka K, Takeda N (2013). Surgical technique to prevent spillage of cyst fluid during operation for cystic ovarian tumors. Pediatr Surg Int.

# Other: New technologies in minimally invasive surgery in childhood cancer surgery

**DOI:** 10.3332/ecancer.2025.2029

**Published:** 2025-10-23

**Authors:** Abdelhafeez H Abdelhafeez, Sabine Sarnacki, Blanc Thomas

**Affiliations:** 1 University of Rochester Medical Center, Golisano Children's Hospital, Department of Surgery, Division of Pediatric Surgery, Rochester, NY 14642, USA; 2 Department of Paediatric Surgery, Urology, and Transplantation, Necker Enfants Malades Hospital, APHP and Paris Cité University, Paris 75015, France

**Keywords:** minimally invasive surgery, paediatric cancer, robotics, single-site surgery, image-guided surgery, surgical precision, tumour resection

## Abstract

**Objective:**

The aim of this guidance is to discuss the advantages of utilising adjunct technologies in minimally invasive surgery and to mitigate risks associated with these technologies in paediatric cancer surgery.

**Methods:**

A literature search was conducted, focusing on robotics, single-site and image-guided surgical approaches in paediatric cancer.

**Results:**

The findings indicate significant improvements in surgical precision, reduced morbidity and enhanced recovery times. Technologies such as robotics, single-site and image-guided surgical approaches have shown promising results in improving the precision of tumour resection.

**Conclusion:**

Integrating advanced technologies into paediatric cancer surgery offers the potential for improved surgical outcomes and quality of life for patients. However, ongoing research and careful implementation are necessary to ensure safety and efficacy.

## Introduction

Surgical resection is a cornerstone of the management of childhood solid tumours due to its critical role in achieving local control and improving survival outcomes [1–3]. Advances in treatment have highlighted the importance of minimally invasive surgical techniques, which aim to reduce the physical and psychological burdens of surgery [4].

Conventional laparoscopic surgery has been widely utilised in paediatric oncology for procedures such as tumour biopsy and resection [5, 6]. Laparoscopic approach typically involves multiple small ports, allowing for improved visualisation and reduced recovery times compared to open surgery. Despite these benefits, traditional laparoscopic approaches can have limitations, including instrument maneuverability, reduced depth perception and increased surgeon fatigue due to ergonomics. Additionally, the complexity of paediatric anatomy poses unique challenges in accessing tumours, particularly in the retroperitoneum of young children [5, 6]. Moreover, incomplete tumour resection remained a challenge since, for decades, intraoperative tumour identification relied on white light visualisation and tactile feedback.

The aim of this review is to discuss new technologies applications in paediatric cancer surgery, aiding more precise maneuverability, less tissue trauma and advanced tumour identification technologies such as robotic, single-site retroperitoneoscopic and image-guided surgical approaches, respectively.

## Robotic-assisted surgery

The application of robotic surgical systems in paediatric surgery has been limited by various intrinsic and extrinsic factors. However, their integration into the surgical management of solid tumours in children presents both unique challenges and significant opportunities. The gradual adoption of robotic technology in paediatric centers is poised to bring about a paradigm shift in surgical care, improving precision of movement and ergonomics [7].

A recent experience from a tertiary paediatric center demonstrated that oncology accounted for 23% of the total robotic surgical procedures since the program's inception. Within the oncology workload, 25% of tumours were treated using a robotic platform [8]. The median age of patients undergoing robotic-assisted surgery (RAS) was 7 years, with one-third of patients aged <5 years, one-third between 5 and 11 years and one-third >11 years. The most common tumour types included endocrine tumours (31%), neuroplastic tumours (29%) and renal tumours (19%). Notably, complication and conversion rates were minimal, underscoring the safety and efficacy of the robotic approach [8].

RAS offers enhanced precision, particularly in the delicate dissection of tumours and in situations where bleeding control is critical. However, the decision to use the robotic platform should be guided by factors such as tumour characteristics, preoperative imaging, tumour extent and the surgeon’s experience. Further research and the development of clearer, evidence-based guidelines are needed to refine its application across a broader range of paediatric solid tumours [8].

Contraindications for RAS in paediatric patients include neurogenic tumours with midline major vessels encasement, extension to the middle mediastinum (such as the pericardium, esophagus or trachea), as well as those involving more than two International Neuroblastoma Risk Factor Classification risk factors or involving unpaired vessels (e.g., the celiac artery or superior mesenteric artery) and/or both renal pedicles. Other contraindications include renal tumours crossing the median sagittal plane or tumours that invade the liver. Robotic surgery is also contraindicated for adrenocortical carcinomas and solid pseudopapillary tumours. Each indication of RAS must be validated by the multidisciplinary tumour board (oncologist, surgeon and radiologist) [8, 9].

Three factors that may influence morbidity in robotic surgery have been analysed [10]. First, small patients (≤15 kg) have been identified as a potential challenge for robotic-assisted laparoscopic surgery due to limited working space [11]. Second, an American Society of Anesthesiologists (ASA) score of ≥3 is used as a marker of patient vulnerability, as it has been associated with increased perioperative morbidity from both surgical and anaesthetic factors [12, 13]. Third, surgical oncology was examined as a potential contributor to patient morbidity, given the complexity of procedures involving limited tumour exposure, the impact of preoperative chemotherapy on tissue dissection, potential multiorgan involvement, the risk of significant intraoperative bleeding and the oncological risks associated with tumour spillage or insufficient surgical margins [14]. In a recent study, none of these factors were linked to postoperative complications, although major complications (Clavien-Dindo ≥ III) may be more common in patients with a higher ASA score [10]. Figure 1 illustrates the locations of the robotic camera and ports for various tumour anatomies.

### Single-site surgery

Single-site surgery has gained attention as a minimally invasive technique in paediatric oncology, particularly for the resection of tumours located in the retroperitoneum. This approach involves making a single small incision through which multiple ports provide access to the retroperitoneal, intraperitoneal or thoracic spaces for tumour removal [15–18] (Figure 2). The main advantage of single-site retroperitoneoscopic surgery is its ability to offer enhanced precision and maneuverability in the confined retroperitoneal space, allowing for careful and meticulous dissection [15–18]. Additionally, the single incision provides a relatively larger working port, which can also be used for specimen retrieval [15–18].

One key benefit of single-site retroperitoneoscopic surgery is faster access to control bleeding and potentially easier conversion into an open procedure if necessary [15–17]. Because the single-site incision is typically larger than those used in multiport access and is more directly aligned with the tumour site, it allows for a more straightforward transition to an open approach if complications occur.

For paediatric patients, this technique offers significant advantages, including reduced scarring, less postoperative pain and faster recovery times; factors that are especially important in children undergoing cancer treatment who benefit from quicker resumption of chemotherapy postoperatively [15–17]. Research has shown that single-site thoracoscopic surgery can be comparable to multiport thoracoscopic surgery in terms of postoperative pain and hospital stay [19]. Interestingly, the advantage of single-site retroperitoneoscopic surgery over conventional laparoscopic surgery may stem more from the retroperitoneal approach itself—avoiding entry into the peritoneal cavity and the need to mobilise bowel—rather than the use of a single site per se [15–17].

However, despite these benefits, the application of single-site surgery in paediatric oncology presents certain challenges. The learning curve to master the retroperitoneal approach, the complexity of retroperitoneal tumour resections, especially for large tumours or those involving critical structures like the renal vasculature, can limit the technique's applicability. Moreover, the process of creating the retroperitoneal space can sometimes result in unintentional breaches of the peritoneal cavity, causing it to collapse and reducing the available working space [20]. In such cases, conversion to laparoscopic access may be required. While peritoneal breaches can be repaired, this is technically challenging during retroperitoneoscopic surgery.

The success of this technique also depends heavily on the surgeon's experience with single-site retroperitoneoscopy and the availability of specialised equipment. Given these challenges, while the technique holds promise, further research and clinical experience are necessary to refine its application and establish comprehensive guidelines for its use in paediatric cancer surgery. Currently, applications for a single-site approach in paediatric cancers include resection of retroperitoneal primary and metastatic tumours, retroperitoneal staging template lymphatic dissection, nephron-sparing resection, resection of mediastinal tumours and resection of pulmonary metastatic nodules.

The launch of the single-port robotic system is a new frontier combining two advanced surgical approaches that would further enhance the precision of childhood cancer surgery [21].

### Image-guidance surgery

Image-guided surgery is increasingly recognised as a transformative tool in the treatment of paediatric cancers, potentially offering greater precision in primary tumour and metastatic deposits identification [22–32]. This technique includes several imaging modalities, with fluorescence-guided surgery and navigation imaging standing out as two of the most promising [22–34]. Fluorescence-guided surgery relies on the use of fluorescent agents that either passively or actively target tumour tissues [25–30]. Passive targeting agents, such as indocyanine green (ICG), are highly sensitive and can highlight tumour areas effectively. However, their specificity is often limited, which can sometimes result in false positives or difficulty distinguishing tumour from surrounding healthy tissue [25–30]. On the other hand, active targeting agents, often coupled with tumour-specific antibodies or receptors, are being explored in paediatric cancers but are still in the early stages of development. Their efficiency and clinical applicability remain to be fully evaluated [22, 24–30].

Augmented reality (AR) and navigation systems are also playing an increasingly important role in enhancing tumour localisation and improving surgeons’ understanding of tumour anatomy before (during the planning phase) and during surgery [22–24]. These technologies allow for the overlay of preoperative imaging data onto the surgical field in real time, creating an intuitive visualisation of tumour structures. Together, AR and navigation systems provide an additional layer of precision, potentially improving the likelihood of complete tumour resection while preserving healthy tissues, which is especially critical in paediatric patients.

In the context of paediatric cancers, ultrasound-guided tumour resection is emerging as a particularly promising tool. High-resolution ultrasound has shown great potential in liver surgeries and nephron-sparing procedures for bilateral Wilms tumour, allowing for localisation of deep tumour nodules [25, 26]. The application of such high-resolution ultrasound could be extended to other paediatric tumours, particularly in soft tissue tumours and sarcomas, where real-time visualisation of tumour boundaries is critical.

While many of these technologies are still evolving and require further refinement and validation, their combined use in paediatric cancer surgeries is paving the way for less invasive, more effective treatments, with the potential for better outcomes and reduced long-term effects for young patients.

## Discussion

The advent of advanced technologies such as robotic surgery, single-site surgery and image-guided surgical approaches is potentially transforming the landscape of paediatric cancer surgery. These technologies may offer improvements in surgical precision, which is a crucial consideration when treating paediatric cancer patients. Minimally invasive techniques, bolstered by these adjunct technologies, present promising opportunities to improve surgical outcomes and minimise the long-term physical and psychological burdens traditionally associated with open surgery.

Robotic surgery offers advantages in terms of dexterity, precision and visualisation. The ability to perform complex dissection with 3D imaging and articulated instruments enables surgeons to navigate complex anatomical structures with greater ease, which is particularly beneficial in the paediatric population, where working space is limited and vascular involvement is not uncommon. The enhanced visualisation and fine motor control provided by robotic systems are invaluable in paediatric oncology, where precision is critical, especially for tumours located near vital structures and major blood vessels.

However, the integration of robotic surgery into paediatric cancer treatment is not without challenges. The limited availability of robotic systems and the high cost of these technologies remain significant barriers to widespread adoption, particularly in resource-limited settings. Additionally, robotic surgery requires specialised training and it may not be suitable for all tumour types or locations, as certain tumours may pose challenges in terms of access or require more extensive resection than robotic systems can currently accommodate. Despite these challenges, the potential benefits in terms of precision and reduced morbidity make robotic surgery an exciting option for paediatric oncologic procedures and warren outcome comparative studies.

Single-site surgery, which involves performing procedures through a single small incision, is slowly gaining traction as a minimally invasive technique in paediatric oncology. One of its key advantages is the reduced scarring compared to traditional multiport laparoscopy, which can improve cosmetic outcomes, an important consideration in paediatric patients. Additionally, the use of a single incision can potentially minimise postoperative pain and expedite recovery, allowing for faster resumption of chemotherapy or other treatments [15–17].

Single-site retroperitoneoscopic surgery has shown promise for tumour resections in the retroperitoneal space, such as neuroblastomas, other neurogenic tumours, metastatic lymphadenopathy and staging lymph nodes sampling [15–17]. The main advantage of single-site retroperitoneoscopic surgery is the ability to offer greater precision and maneuverability in confined anatomical spaces. Moreover, the relatively larger working port provided by the single incision facilitates specimen retrieval and bleeding control, which can be more challenging with multiport access. This approach can also allow for easier conversion to an open procedure in retroperitoneal surgery if complications arise, since the incision is generally more directly oriented toward the tumour site.

However, the applicability of single-site surgery is not without limitations. The complexity of paediatric retroperitoneal surgeries, especially when dealing with large tumours or tumours involving critical structures such as vessels, can make this approach challenging. The risk of unintentional breaches into the peritoneal cavity during the creation of the retroperitoneal space is another concern, as it can reduce the available working space and necessitate conversion to laparoscopic or open surgery [19]. As with robotic surgery, the surgeon’s experience and expertise play a critical role in determining whether single-site surgery is a viable option for a given patient. More research is needed to refine this technique and establish best practice guidelines for its use in paediatric oncology.

Image-guided surgery is becoming increasingly important in paediatric oncology, offering the potential for preoperative planning, real-time tumour localisation and enhanced surgical precision. Fluorescence-guided surgery, for instance, has shown promise in localisation of tumour and metastatic deposits, which is particularly valuable in cases where traditional white light visualisation may be insufficient [26]. Fluorescent agents like ICG can highlight tumour areas with high sensitivity, though their specificity remains limited, making it challenging to clearly distinguish between tumour and surrounding healthy tissue [26]. The development of active targeting agents, such as those coupled with tumour-specific antibodies, holds promise for increasing the specificity and accuracy of fluorescence-guided surgery, though these agents are still in the early stages of clinical evaluation in paediatric cancers [22].

In addition to fluorescence guidance, other image-guided techniques such as AR and intraoperative navigation are increasingly being incorporated into paediatric cancer surgery. These technologies allow surgeons to overlay preoperative imaging data onto the surgical field in real time, potentially enhancing tumour localisation and improving the accuracy of resection.

Ultrasound-guided surgery has been particularly useful in liver surgeries and nephron-sparing procedures for bilateral Wilms tumour. Ultrasound provides real-time visualisation of the tumour boundaries, enabling the surgeon to precisely delineate tumour anatomy during resection. The integration of intraoperative cross-sectional imaging into the surgical workflow, however, remains complex due to challenges in maintaining real-time dynamic imaging without disrupting the surgical process. Nevertheless, high-resolution ultrasound and other imaging technologies have the potential to expand the scope and effectiveness of minimally invasive paediatric cancer surgery.

While the application of these advanced technologies in paediatric cancer surgery is promising, several challenges remain. The integration of new technologies into clinical practice requires substantial investment in equipment and training. Additionally, long-term studies are needed to establish the safety, efficacy and cost-effectiveness of these techniques. Robotic surgery, single-site approaches and image-guided surgery can potentially reduce morbidity and improve surgical outcomes; however, evidence supporting these advantages yet remains of very low quality. Moreover, evidence examining the long-term impact of these techniques on overall survival and recurrence rates in paediatric cancer patients is lacking.

As these technologies continue to evolve, it will be essential to ensure that they are accessible to all paediatric cancer patients, regardless of socioeconomic status. Ethical considerations, including cost, access and informed consent, must also be addressed as these technologies become more integrated into clinical practice.

## Conclusion

The integration of robotic surgery, single-site techniques and advanced image guidance into minimally invasive paediatric cancer surgery marks a significant advancement in the field. These technologies hold the potential to enhance surgical precision, reduce patient morbidity and improve the overall quality of life for patients. However, to fully realise their benefits, ongoing research, collaboration and innovation among clinicians, researchers and technologists are essential. This will ensure that these techniques are refined, standardised and implemented safely across paediatric oncology surgery. As the field continues to evolve, the combination of cutting-edge technology with expert surgical care will pave the way for a new era of precision surgery—one that offers more effective, less invasive treatments with better outcomes for paediatric cancer patients.

## Conflicts of interest

The authors declare that there are no conflicts of interest related to this work.

## Funding

This research received no specific grant from any funding agency in the public, commercial or not-for-profit sectors.

## Figures and Tables

**Figure 1.** Anatomical sites of tumours and corresponding robotic resection camera and port approaches.

**Figure 2.** Single-site retroperitoneoscopic approach.

## References

[1] Polites SF, Rhee DS, Seitz G (2024). Contemporary surgical management of pediatric non-rhabdomyosarcoma soft tissue sarcoma. Pediatr Blood Cancer.

[2] Fischer J, Pohl A, Volland R (2017). Complete surgical resection improves outcome in INRG high-risk patients with localized neuroblastoma older than 18 months. BMC Cancer.

[3] Gow KW, Lautz TB, Malek MM (2024). Children's oncology group's 2023 blueprint for research: surgery. Pediatr Blood Cancer.

[4] Dieffenbach BV, Murphy AJ, Liu Q (2023). Cumulative burden of late, major surgical intervention in survivors of childhood cancer: a report from the childhood cancer survivor study (CCSS) cohort. Lancet Oncol.

[5] Acker SN, Bruny JL, Garrington TP (2015). Minimally invasive surgical techniques are safe in the diagnosis and treatment of pediatric malignancies. Surg Endosc.

[6] Abdelhafeez (2019). Minimally invasive surgery in pediatric surgical oncology: practice evolution at a contemporary single-center institution and a guideline proposal for a randomized controlled study. J Laparoendosc Adv Surg Tech A.

[7] Svetanoff WJ, Carter M, Diefenbach KA (2024). Robotic-assisted pediatric thoracic and abdominal tumor resection: an initial multicenter review. J Pediatr Surg.

[8] Blanc T (2024). Robotic surgery in paediatric oncology: expanding boundaries and defining relevant indications. J Pediatr Surg.

[9] Li P, Zhou H, Cao H (2021). Robot-assisted laparoscopic management of bladder/prostate rhabdomyosarcoma in children: initial series and 1-year outcomes. J Endourol.

[10] Vinit N (2023). Adverse events and morbidity in a multidisciplinary pediatric robotic surgery program. A prospective, observational study. Ann Surg.

[11] Molinaro F (2019). Low weight child: can it be considered a limit of robotic surgery? Experience of two centers. J Laparoendosc Adv Surg Tech A.

[12] Fantola G, Brunaud L, Nguyen-Thi PL (2017). Risk factors for postoperative complications in robotic general surgery. Updates Surg.

[13] Murat I, Constant I, Maud’Huy H (2004). Perioperative anaesthetic morbidity in children: a database of 24 165 anaesthetics over a 30-month period. Paediatr Anaesth.

[14] Blanc T, Meignan P, Vinit N (2022). Robotic surgery in pediatric oncology: lessons learned from the first 100 tumors—a nationwide experience. Ann Surg Oncol.

[15] Mansfield SA, Murphy AJ, Talbot L (2020). Alternative approaches to retroperitoneal lymph node dissection for paratesticular rhabdomyosarcoma. J Pediatr Surg.

[16] Pio L, Melero Pardo AL, Zaghloul T (2023). Retroperitoneoscopic or transperitoneal approach for neurogenic and adrenal tumors in children? A comparison on the way to enhanced recovery in pediatric surgical oncology. J Pediatr Surg.

[17] El-Gohary Y, Mansfield S, Talbot L (2020). Single-site retroperitoneoscopy in pediatric metastatic lymphadenopathy. J Pediatr Surg.

[18] Pio L, Zaghloul T, Zaghloul T (2023). Indocyanine green fluorescence-guided lymphadenectomy with single site retroperitoneoscopy in children. J Pediatr Urol.

[19] Fernandez-Pineda I, Seims AD, VanHouwelingen L (2019). Modified uniportal video-assisted thoracic surgery versus three-port approach for lung nodule biopsy in pediatric cancer patients. J Laparoendosc Adv Surg Tech A.

[20] Theilen TM, Paran TS, Rutigliano D (2011). Experience with retroperitoneoscopy in pediatric surgical oncology. Surg Endosc.

[21] Marshall MB, Wee JO, Soukiasian HJ (2024). Initial evaluation of the safety and performance of single-port robotic-assisted thymectomy via subxiphoid incision. Ann Thorac Surg.

[22] Fusco JC, Abdelhafeez AH, Krauel L (2024). Imaging adjuvants in pediatric surgical oncology. Pediatr Blood Cancer.

[23] Valls-Esteve A, Adell-Gómez N, Pasten A (2023). Exploring the potential of three-dimensional imaging, printing, and modeling in pediatric surgical oncology: a new era of precision surgery. Children (Basel, Switzerland).

[24] Shah NR, Weadock WJ, Williams KM (2024). Use of modern three dimensional imaging models to guide surgical planning for local control of pediatric extracranial solid tumors. Pediatr Blood Cancer.

[25] Pio L, Wijnen MHWA, Giuliani S (2023). Identification of pediatric tumors intraoperatively using indocyanine green (ICG). Ann Surg Oncol.

[26] Abdelhafeez A, Talbot L, Murphy AJ (2021). Indocyanine green-guided pediatric tumor resection: approach, utility, and challenges. Front Pediatr.

[27] Abdelhafeez AH, Murphy AJ, Brennan R (2022). Indocyanine green-guided nephron-sparing surgery for pediatric renal tumors. J Pediatr Surg.

[28] Goldstein SD, Heaton TE, Bondoc A (2021). Evolving applications of fluorescence guided surgery in pediatric surgical oncology: a practical guide for surgeons. J Pediatr Surg.

[29] Abdelhafeez AH, Mothi SS, Pio L (2023). Feasibility of indocyanine green guided localization of pulmonary nodules in children with solid tumors. Pediatr Blood Cancer.

[30] Richard C, White S, Williams R (2023). Indocyanine green near infrared-guided surgery in children, adolescents, and young adults with otolaryngologic malignancies. Auris Nasus Larynx.

[31] Abdelhafeez AH, Davidoff AM, Murphy AJ (2022). Fluorescence-guided lymph node sampling is feasible during upfront or delayed nephrectomy for Wilms tumor. J Pediatr Surg.

[32] Pio L, Richard C, Zaghloul T (2024). Sentinel lymph node mapping with indocyanine green fluorescence (ICG) for pediatric and adolescent tumors: a prospective observational study. Sci Rep.

[33] Johnston ME, Farooqui ZA, Nagarajan R (2023). Fluorescent-guided surgery and the use of indocyanine green sentinel lymph node mapping in the pediatric and young adult oncology population. Cancer.

[34] Jeremiasse B, Scheltinga CEJT, Smeele LE (2023). Sentinel lymph node procedure in pediatric patients with melanoma, squamous cell carcinoma, or sarcoma using near-infrared fluorescence imaging with indocyanine green: a feasibility trial. Ann Surg Oncol.

[35] Felsted AE, Shi Y, Masand PM (2015). Intraoperative ultrasound for liver tumor resection in children. J Surg Res.

[36] Aldrink JH, Cost NG, Mcleod DJ (2018). Technical considerations for nephron-sparing surgery in children: what is needed to preserve renal units?. J Surg Res.
